# Endonuclease G preferentially cleaves 5-hydroxymethylcytosine-modified DNA creating a substrate for recombination

**DOI:** 10.1093/nar/gku1032

**Published:** 2014-10-29

**Authors:** Adam B. Robertson, Julia Robertson, Markus Fusser, Arne Klungland

**Affiliations:** 1 Institute of Medical Microbiology, Oslo University Hospital, Rikshospitalet, Norway; 2 Institute of Basic Medical Sciences, University of Oslo, PO Box 1018 Blindern, N-0315 Oslo, Norway

## Abstract

5-hydroxymethylcytosine (5hmC) has been suggested to be involved in various nucleic acid transactions and cellular processes, including transcriptional regulation, demethylation of 5-methylcytosine and stem cell pluripotency. We have identified an activity that preferentially catalyzes the cleavage of double-stranded 5hmC-modified DNA. Using biochemical methods we purified this activity from mouse liver extracts and demonstrate that the enzyme responsible for the cleavage of 5hmC-modified DNA is Endonuclease G (EndoG). We show that recombinant EndoG preferentially recognizes and cleaves a core sequence when one specific cytosine within that core sequence is hydroxymethylated. Additionally, we provide *in vivo* evidence that EndoG catalyzes the formation of double-stranded DNA breaks and that this cleavage is dependent upon the core sequence, EndoG and 5hmC. Finally, we demonstrate that the 5hmC modification can promote conservative recombination in an EndoG-dependent manner.

## INTRODUCTION

The existence of 5-hydroxymethylcytosine (5hmC) in mammalian DNA was shown in 1972 (1). At that time the function of 5hmC was relegated to the status of a non-mutagenic DNA lesion (2). The significance of the 5hmC modification was realized more than 30 years later, when 5hmC was identified in specialized Purkinje neurons (3) and this modification was implicated in the oxidative demethylation of 5-methylcytosine (5meC) (4).

Tet1 was described as the founding member of a class of iron-dependent dioxygenases that catalyzed the formation of 5hmC from 5meC (4). Later it was shown that all three known Tet enzymes—Tet1, Tet2 and Tet3—could catalyze this reaction (5). Further research demonstrated the importance of 5hmC and the Tet enzymes in cancer development when it was shown that deficiencies in Tet2 cause aberrant genomic 5hmC patterns; this led to certain myeloid cancers (6–8). Other research has shown that 5hmC is present in stem cells (9) and has suggested that 5hmC is necessary for stem cell renewal (10), transcriptional regulation (11–14) and is an intermediate in the oxidative demethylation of 5meC. This demethylation is accomplished either by using the base excision repair machinery (15,16) or by successive oxidation steps leading to 5-carboxycytosine (17). 5-carboxycytosine can be removed by the thymine DNA glycosylase (18,19) or an, as yet, unidentified 5-carboxycytosine decarboxylase (20).

Recent genome-wide mapping of the positions of 5hmC have demonstrated that 5hmC is present at the highest amounts in gene bodies and to a lesser extent at the promoters of genes (21–25). The functional significance of the positioning of 5hmC remains unclear. Early models suggest that 5hmC at gene promoters could alleviate transcriptional repression by 5meC (26); however, this model is not consistent with several current studies that demonstrate that 5hmC at gene promoters reduces gene expression (11–13,22). 5hmC within gene bodies has a limited effect on gene expression (12) and it has been suggested that 5hmC within gene bodies may be involved in RNA splicing.

Endonuclease G (EndoG) was initially purified from the mitochondria of bovine heart (27) and calf thymus nuclear extracts (28). It gained its name for its ability to preferentially cleave guanosine (G)-rich DNA regions (29). Later it was reported that EndoG localizes primarily to the mitochondria (30); however, more recent reports suggest that EndoG also resides in the nucleus (31–33). EndoG was shown to be involved in the removal of RNA primers created during mitochondrial DNA replication (30). After this finding, it was also demonstrated that EndoG could be a nuclease involved in the execution of apoptosis (34). However, two EndoG knockout mice failed to show any apoptotic defects or mtDNA replication errors (35,36). Several recent reports implicate EndoG in specialized DNA recombination events (31–33,37).

In this report, we identify and purify an activity in mouse liver extracts that preferentially cleaves 5hmC-modified DNA. We show that EndoG is required for this 5hmC cleavage activity. We characterize a preferred sequence of EndoG-mediated cleavage and demonstrate that this cleavage is greatly increased in the presence of cytosine hydroxymethylation. We show that EndoG is necessary and sufficient for 5hmC-mediated cleavage both *in vitro* and *in vivo*. Finally, we demonstrate that 5hmC-modified DNA undergoes conservative recombination more frequently than unmodified DNA and that this increase in recombination is dependent on EndoG.

## MATERIALS AND METHODS

### Ethics statement

The mouse studies were approved by Folkehelseinstituttet; and as such comply with Norwegian National and Local Regulations. Mice were euthanized by cervical dislocation under isoflurane anesthesia.

### Antibodies

The following antibodies were used for this study: β-actin (western blot 1:1000, Enzo Life Science, ADI-905–733), CoxIV (western blot 1 μg/ml, Invitrogen, 459600), EndoG (western blot 1:200, Santa Cruz Biotechnology, sc-26923), Histone H3 (western blot 1:250, Diagenode, cs-135–100), γ-H2AX (immunofluorescence 0.2 μg/ml, Millipore, 05–636), 53BP1 (immunoflourescence 1:100, Abcam, ab36823).

### Organ nuclear extract preparation

Organs were harvested from euthanized 3-month-old C57bl/6J or C57bl/6J *Endog^−/−^* mice. *Endog^−/−^* mice were a gift from Esther Dupont-Versteegden; their creation was described previously (36). Organ extracts were prepared as previously described (3,38). The purity of liver nuclear extracts (LiNE) was evaluated by western analysis followed by probing with mitochondrial complex IV, Histone H3 and EndoG antiserum (Supplementary Figure S1). Wild-type and *EndoG^−/−^* extracts were blotted and probed with actin antiserum to show equal loading (Supplementary Figure S2A). Adenosine triphosphate (ATP) hydrolysis assays were used to demonstrate enzymatically active extracts (Supplementary Figure S2B).

### ATP hydrolysis reactions

Reactions (20 μl) containing 1.2 μg of wild-type or *Endog^−/−^* LiNE, 3 mM γ-[^32^P]-ATP (10 mCi/mmol), 25 mM Tris (pH 7.5), 3 mM MgCl_2_, 20 mM NaCl and 5 mM β-mercaptoethanol were incubated at 37°C for 120 min. Reactions were terminated by the addition of 10 μl of a solution containing 33 mM ethylenediaminetetraacetic acid (EDTA) (pH 8.0), 6 mM ATP and 6 mM K_2_PO_4_. Two microliters of each reaction was spotted on PEI cellulose F plates (Millipore) and allowed to dry. The plates were resolved in a TLC chamber containing 1 M formic acid and 0.8 M LiCl. The plates were dried and placed on a phosphor screen (Molecular Dynamics) overnight. The phosphor screen was imaged with a Typhoon Scanner (Molecular Dynamics) and quantified using ImageQuant Software (GE Healthcare).

### Stable cell lines

Note that 1.5 × 10^5^ HeLa cells were plated in a 6-well dish and allowed to attach for 24 h. Cells were then transfected with 1 μg EndoG shRNA (Santa Cruz) or pcDNA3 Tet2 catalytic domain (Tet2 CD) (a generous gift from Yi Zhang, construction described in previously (5)). After 48 h, cells were split and selected with 2 μg/ml Puromycin for cells transfected with EndoG shRNA, or the relevant control empty vector or 200 μg/ml Neomycin for cells transfected with pcDNA3 Tet2 CD. After selection for 10–14 days 40 individual colonies were picked and evaluated for expression. Cells expressing the lowest EndoG and/or the highest Tet2 CD, as determined by quantitative polymerase chain reaction (qPCR), were used for future experiments. The doubly transfected cell lines were created as above except the HeLa cells expressing the EndoG shRNA or the empty vector shRNA were seeded and pcDNA3 Tet2 CD was transfected into these cell lines.

### siRNA

Note that 15 000 HeLa cells were transfected using Lipofectamine RNAiMax (Invitrogen) with either control (Invitrogen cat nr. AM4611), EndoG siRNA 1 (Invitrogen cat nr. 4390824 id s707) or EndoG siRNA 2 (Invitrogen cat nr. 4390824 id s708) at a concentration of 30 nM. At the appropriate time point total RNA was harvested and reverse transcribed using a High capacity cDNA synthesis kit (Applied Biosystems). Alternatively, cells were subject to immunofluorescence (described below). EndoG and Tet2 CD-specific RNAs were quantified using a Taqman probe designed to detect EndoG or Tet2 CD. Knockdown efficiency was calculated as the specific transcript in the treated sample divided by the RNA present in the control sample multiplied by 100%.

### 5hmC quantification

DNA was purified from each HeLa cell line using organic extraction, followed by an ethanol precipitation. The amount of 5hmC present in each DNA sample was quantified using a Quest 5hmC detection kit (Zymo Research) according to the manufacturer's instructions.

### DNA substrates

Note that 2.7 kbp substrates were created by PCR amplifying the derivatives of the pUC19 vector (Sequence and PCR primers in Supplementary Tables S1 and S2). Substrates containing 5hmC were amplified in the presence of d5hmCTP (Bioline) instead of dCTP.

The short substrates were created by annealing oligonucleotides (Supplementary Table S2). The annealed oligonucleotides were cloned into pCR2.1-Topo according to the manufacturer's instructions. The short substrates were PCR amplified using primers that amplify the region of interest (Supplemental Table S1). Substrates containing all 5meC were synthesized using d5mCTP (Fermentas) instead of dCTP and Phusion Polymerase (Finnzymes) instead of Pfu Turbo Polymerase. Substrates containing 5hmC were amplified in the presence of d5hmCTP (Bioline) instead of dCTP. The *in vivo* cut and control substrates were created by PCR amplification of the entire pCR2.1-Topo vector with the cut or control substrate cloned into the vector.

Recombination substrates were created by PCR amplification of Substrate A and Substrate B (Supplementary Table S1). Substrates containing 5hmC were amplified in the presence of d5hmCTP (Bioline) instead of dCTP.

Oligonucleotide annealing reactions (50 μl) containing 200 pmol of each oligonucleotide were annealed by heating to 95°C for 5 min and cooled slowly to room temperature over 3 h. dsOligonucleotides were resolved on 15% non-denaturing polyacrylamide gel electrophoresis (PAGE), identified using ultraviolet shadowing and extracted.

Substrates were labeled using γ-[^32^P]ATP (20 μCi) and T4 polynucleotide kinase (NEB) according to the manufacturer's instructions. Unincorporated ATP was removed using a nucleotide clean kit (Qiagen).

### EndoG and dEndoGI cloning and purification

Full-length *EndoG* was reverse transcribed from *Mus musculus* liver RNA, PCR amplified and cloned into pET28a (pET28a-EndoG). pET28a-EndoG/H128A was created using Quickchange (Strategene) site directed mutagenesis. dEndoGI was reverse transcribed from *Drosophila melanogaster* total RNA, PCR amplified and cloned into pET28a. Rossetta(DE3) ((F^−^ ompT hsdS_B_ (R_B_^−^ m_B_^−^) gal dcm λ (DE3 [lacI lacUV5-T7 gene 1 ind1 sam7 nin5]) pLysSRARE (Cam^R^)) harboring pET28a-EndoG, pET28a-EndoG/H128A, pET28a-dEndoGI were grown in Studier Autoinducing Media (39) at 37°C until the culture reached an A_600_ of 0.8. The culture was then shifted to 18°C for 16 h. The cells were harvested by centrifugation and suspended in 10 ml EndoG wash buffer (25 mM Hepes pH 7.9, 500 mM NaCl, 5 mM Imidazole and 10% (v/v) glycerol) for every 500 ml cells grown. All further steps were carried out at 4°C unless otherwise stated. The cells were incubated for 60 min with lysozyme added to a final concentration of 200 ng/μl. TritionX-100 was added to a final concentration of 0.1% (v/v) and the cells were heated briefly to 20°C. The cell lysate was then sonicated three times for 30 s with a 2-min rest on ice between each sonication. The cell lysate was cleared by centrifugation at 10 000 × *g* for 30 min. The supernatant was applied to a 2.5 ml talon resin (BD Biosciences) and washed with EndoG wash buffer until the flow-through contained no detectable proteins. EndoG was eluted from the talon column using EndoG elution buffer (25 mM Hepes pH 7.9, 500 mM NaCl, 200 mM Imidazole and 10% (v/v) glycerol). The resulting purified EndoG or EndoGI was dialyzed against 100 volumes of EndoG storage buffer (250 mM NaCl, 25 mM Hepes pH 7.9, 1 mM EDTA pH 8.0 and 50% (v/v) glycerol).

### Cleavage assays

Reactions (20 μl) containing 4.5 ng DNA substrate or the indicated amount of dsoligonucleotide, 20 mM Hepes (pH 7.9), 1 mM EDTA pH 8.0, 3 mM MgCl_2_, 10 mM 2-mercaptoethanol, 4% (v/v) glycerol, 1% (v/v) Triton X-100, 0.2 ng poly(dIdC) (unless otherwise stated) and a titration of the nuclear extracts from 25 to 100 ng or the indicated amount of purified EndoG or EndoG/H128A were incubated at 37°C for 30 min. Reactions were stopped by the addition of 4 μl stop solution (50 mM Tris (pH 8.0), 2% (w/v) sodium dodecyl sulphate (SDS), 100 mM EDTA (pH 8.0) and 1 μg Proteinase K). The stop solution was allowed to incubate with the reaction at 42°C for at least 30 min. After incubation the entire reaction mixtures were resolved on a 0.8% or a 1.2% agarose or 15% non-denaturing PAGE. The agarose gels were incubated with 40% (v/v) methanol, 10% (v/v) acetic acid and 3% (v/v) glycerol for at least 60 min. The gels were then dried and were imaged on a phosphor screen.

### Kinetic parameters

Cleavage assays were performed as described above with a decreasing titration of the indicated dsDNA substrate (19, 6.3, 2.1 and 0.70 nM) with 1.3 pmol EndoG. Reaction velocities were plotted against substrate concentration. *K*_m_ and *V*_max_ were computed using a Lineweaver–Burk plot. *K*_cat_ values were computed as *V*_max_ divided by the EndoG concentration. The specificity constant (*K*_cat_/*K*_m_) was computed by dividing the *K*_cat_ by the *K*_m_.

### Competition assays

Reactions (20 μl) containing 4.5 ng DNA substrate, 40 ng LiNE, 20 mM HEPES (pH 7.9), 1 mM EDTA (pH 8.0), 3 mM MgCl_2_, 10 mM 2-mercaptoethanol, 4% (v/v) glycerol, 1% (v/v) Triton X-100 and a titration of poly(dIdC) from 0.1-fold molar excess to a 10-fold molar excess was incubated at 37°C for 30 min. Reactions were stopped by the addition of 4 μl stop solution (50 mM Tris (pH 8.0), 2% (w/v) SDS, 100 mM EDTA (pH 8.0) and 1 μg Proteinase K). The stop solution was allowed to incubate with the reaction at 42°C for at least 30 min. After incubation the entire reaction mixtures were resolved on either a 0.8% or a 1.2% agarose gel. The agarose gel was incubated with 40% (v/v) methanol, 10% (v/v) acetic acid and 3% (v/v) glycerol for at least 60 min. The gels were then dried and were imaged on a phosphor screen.

### Gel shift assays

Reactions (10 μl) containing 4.5 ng each short (130 bp) cytosine, fully 5meC and fully 5hmC substrates (5hmC), 20 mM HEPES (pH 7.9), 5 mM EDTA pH 8.0, 10 mM 2-mercaptolethanol, 4% (v/v) glycerol, 1% (v/v) Triton X-100, the indicated concentration of EndoG were incubated on ice (4°C). After 5 min, 5 μl of 75% glycerol was added to each reaction. Reactions were resolved on 6% Native-PAGE at 4°C for 16 h. Gels were incubated with 40% (v/v) methanol, 10% (v/v) acetic acid and 3% (v/v) glycerol for at least 60 min. The gels were then dried and were imaged on a phosphor screen.

### Cleavage activity purification

Mouse LiNE were prepared from 54 mice as described above. All steps were carried out at 4°C. Pooled nuclear extracts were dialyzed twice against 100 volumes of Buffer A (40 mM KCl, 0.2 mM EDTA (pH 8.0), 20 mM Hepes (pH 7.9) and 20% (v/v) glycerol). The dialyzed fraction was centrifuged at 13 000 × *g* for 15 min and the soluble fraction was applied to a 4 ml heparin agarose column equilibrated with buffer A. The heparin column was eluted with a 40 ml linear gradient from 0.04 to 1.0 M KCl. Fractions were assayed for 5hmC cleavage activity and relevant fractions (eluting at 50–120 mM KCl) were pooled and dialyzed twice against 100 volumes of Buffer A. The dialyzed fraction was applied to 4 × 1 ml HiTrapQ columns (Amersham Biosciences) equilibrated with buffer A. The HiTrapQ column was eluted with a 40 ml linear gradient from 0.04 to 0.75 M KCl. Fractions were assayed for 5hmC cleavage activity and relevant fractions (eluting at 50–100 mM KCl) were pooled and dialyzed twice against 100 volumes of Buffer A. The dialyzed fraction was applied to 3 × 1 ml HiTrap SP columns (Amersham Biosciences) equilibrated with buffer A. The HiTrap SP column was eluted with a 30 ml linear gradient from 0.04 to 1.0 M KCl. Fractions were assayed for 5hmC cleavage activity and relevant fractions (eluting at 60–80 mM KCl) were pooled and dialyzed twice against 100 volumes of Buffer B (250 mM KCl, 0.2 mM EDTA (pH 8.0), 20 mM Hepes (pH 7.9) and 20% (v/v) glycerol). The pool was concentrated to 500 μl using a centricon MWCO 10 000 (Millipore) and applied to a Superdex 75 column equilibrated in Buffer B. The Superdex 75 column was eluted with 35 ml buffer B. Fractions containing the highest 5hmC-specific cleavage activity (between 80 and 100 kDa) were resolved using 4–16% SDS-PAGE. The entire gel lane was subjected to nanoLC-ESI-MS/MS analysis.

### Protein identification

Protein identification using nanoLC-ESI-MS/MS was performed by Proteome Factory (Proteome Factory AG, Berlin, Germany). The mass spectrometry (MS) system consisted of an Agilent 1100 nanoLC system (Agilent, Waldbronn, Germany), NanoMate 100 (Advion, Ithaca, USA) and a Finnigan LTQ-FT mass spectrometer (ThermoFisher, Bremen, Germany). Proteins were in-gel digested by trypsin (Promega, Mannheim, Germany) and applied to nanoLC-ESI-MS/MS. Peptides were trapped and desalted on an enrichment column (Zorbax SB C18, 0.3 mm × 5 mm, Agilent) for 5 min using 1% acetonitrile/0.5% formic acid as eluent. The peptides were separated on a Zorbax 300 SB C18, 75 μm × 150 mm column (Agilent) using an acetonitrile/0.1% formic acid gradient from 5% to 40% acetonitrile over 40 min. MS spectra were automatically recorded by the mass spectrometer according to manufacturer's instrument settings for nanoLC-ESI-MS/MS analyses. Proteins were identified using MS/MS ion search of the Mascot search engine (Matrix Science, London, England) and nr protein database (National Center for Biotechnology Information, Bethesda, USA).

### *In vivo* cleavage assays

HeLa cells were transfected with the appropriate siRNA as described above. After incubation for 48 h HeLa cells were transfected using FuGene (Invitrogen) with 500 ng of the appropriate substrate. After a 20-h incubation the total RNA and DNA was isolated from the HeLa cells. qPCR was performed on 5 ng DNA using the cut primer set and the control region primer set. The fraction cut is represented as the quantity of the cut primer set as a fraction of the control primer set.

### γ-H2AX and 53BP1 foci assays

Twenty thousand cells of each stably transfected HeLa cell line or transiently siRNA knocked down cells were plated in a single well of a Lab-Tek II 8-well chamber slide (Thermo Scientific) and allowed to settle overnight at 37°C in a humidified 5% CO_2_ incubator. Cells were washed with phosphate-buffered saline and fixed with ice cold 100% (v/v) methanol and placed at −20°C for 10 min. Cells were washed once with 300 μl Tris buffered saline (TBS). Cells were permeabilized with 300 ml TBST (TBS and 0.5% (v/v) TritonX-100) for 10 min at 25°C. Slides were blocked with blocking solution (300 μl TBS, 0.05% (v/v) Tween20 and 5% (w/v) bovine serum albumin) for 1 h at 25°C. One hundred microliters γ-H2AX antiserum in blocking solution was added to the slides and incubated at 25°C for 1 h. Slides were washed three times in TBST for 10 min. One hundred microliters of Alexa Fluor 594 goat anti-mouse immunoglobulin G (IgG; Invitrogen) at a titer of 1:500 in blocking solution was added to the cells for 1 h at 25°C. When relevant one hundred microliters of 53BP1 antiserum in blocking solution was added to the slides and incubated at 25°C for 1 h. Slides were washed three times in TBST for 10 min. One hundred microliters of Alexa Fluor 488 goat anti-rabbit IgG (Invitrogen) at a titer of 1:500 in blocking solution was added to the cells for 1 h at 25°C. Slides were washed with 300 μl TBST for 10 min, 300 μl TBST and 5 mg/ml DAPI for 10 min and 300 μl TBST for 10 min. Slides were mounted with Mowiol (Calbiochem) and dried at 25°C in the dark for at least 2 h. Slides were visualized with an Axio Observer.Z1 Axio inverted microscope (Carl Zeiss) and quantified using AxioVision Software Release 4.8 (Carl Zeiss). γ-H2AX foci were counted from 180 to 220 cells of each cell line in duplicate. γ-H2AX and 53BP1 foci were counted in 40–60 cells of EndoG siRNA knockdowns.

### Irradiation of HeLa cells

Twenty thousand HeLa cells were plated in each well of a Lab-Tek II 8-well chamber slide (Thermo Scientific) and allowed to settle overnight at 37°C in a humidified 5% CO_2_ incubator. Cells were irradiated in a Gammacell 3000 Elan (MDS Nordicon) with a dose of either 1 or 3 Gy. Cells were shifted to a 37°C humidified 5% CO_2_ incubator for 30 min. Cells were washed with phosphate-buffered saline and fixed with ice cold 100% (v/v) methanol and placed at −20°C for 10 min. Cells were washed once with 300 μl TBS. Cells were permeabilized with 300 ml TBST (TBS and 0.5% (v/v) TritonX-100) for 10 min at 25°C. Slides were blocked with blocking solution (300 μl TBS, 0.05% (v/v) Tween20 and 5% (w/v) bovine serum albumin) for 1 h at 25°C. One hundred microliters γ-H2AX antiserum and 53BP1 antiserum in blocking solution was added to the slides and incubated at 25°C for 1 h. Slides were washed three times in TBST for 10 min. One hundred microliters of Alexa Fluor 594 goat anti-mouse IgG (Invitrogen) and Alexa Fluor 488 donkey anti-rabbit IgG, both at a titer of 1:500, in blocking solution was added to the cells for 1 h at 25°C. Slides were washed with 300 μl TBST for 10 min, 300 μl TBST and 5 mg/ml DAPI for 10 min and 300 μl TBST for 10 min. Slides were mounted with Mowiol (Calbiochem) and dried at 25°C in the dark for at least 2 h. Slides were visualized with an Axio Observer.Z1 Axio inverted microscope (Carl Zeiss) and quantified using AxioVision Software Release 4.8 (Carl Zeiss).

### Growth curves and viability measurements of stable cell lines

Each cell line was plated in a 6-well dish at a density of 10^5^ cells per well. At the time point indicated cells were harvested and mixed with an equivalent volume of 0.4% Trypan blue. Cell counts and viability was measured using a countess cell counter (Invitrogen).

### Apoptosis assays

Twenty thousand cells of each stably transfected HeLa cell line per well were plated in an Lab-Tek II 8-well chamber slide (Thermo Scientific) and allowed to attach overnight at 37°C in a humidified 5% CO_2_ incubator. The Click-iT TUNEL Alexa Fluor 594 Imaging Assay Kit (Life Technologies) was used according to the manufacturer's instructions. Slides were mounted with Mowiol (Calbiochem) and dried at 25°C in the dark for at least 2 h. Slides were visualized with an Axio Observer.Z1 Axio inverted microscope (Carl Zeiss) and quantified using AxioVision Software Release 4.8 (Carl Zeiss). Data presented are the results of counting between 200 and 250 cells from each cell line in duplicate.

### Recombination assays

Reactions (50 μl) containing 100 ng of Substrate A and 100 ng Substrate B, either unmodified or fully 5hmC-modified, 15.4 μg LiNE, 20 mM HEPES (pH 7.9), 1 mM EDTA pH 8.0, 3 mM MgCl_2_, 10 mM KCl, 10 mM 2-mercaptoethanol, 4% (v/v) glycerol, were incubated for 15 min at 37°C. Reactions were terminated by the addition of 10 μl stop solution (50 mM Tris (pH 8.0), 2% (w/v) SDS, 100 mM EDTA (pH 8.0) and 1 μg Proteinase K) and incubated at 42°C for at least 30 min. DNA was purified using a Qiagen PCR clean kit. Two qPCR reactions were run on each reaction: control region and recombined region (primers in Supplementary Table S2) were completed as previously described (40). The percent recombinant was computed using the formula: ((recombined region quantity)/(control region recovered)) × 100%. The value recorded for the reaction incubated in the absence of LiNE was subtracted from the result of this calculation.

### Sequencing of recombinant qPCR reactions

qPCR amplification reactions were resolved on 1.4% agarose gel. The only band present in the gel at 420 bp was excised and the agarose was removed using a gel extraction kit (Qiagen). This band was PCR amplified and cloned into pCR4-Topo (Invitrogen) according to the manufacture's instructions. Twelve colonies from each cloning reaction (48 in total) were grown and the resulting plasmids were purified using a miniprep kit (Qiagen). Isolated plasmid clones were sequenced using Sanger sequencing.

### Statistical analysis

A two-tailed Student's *t*-test was used to evaluate statistical significance. *P*-values less than 0.05 were considered statistically different.

## RESULTS

### Identification of an activity from mammalian nuclear extracts that produces specific cleavage products from 5hmC-modified DNA

**Figure 1.**
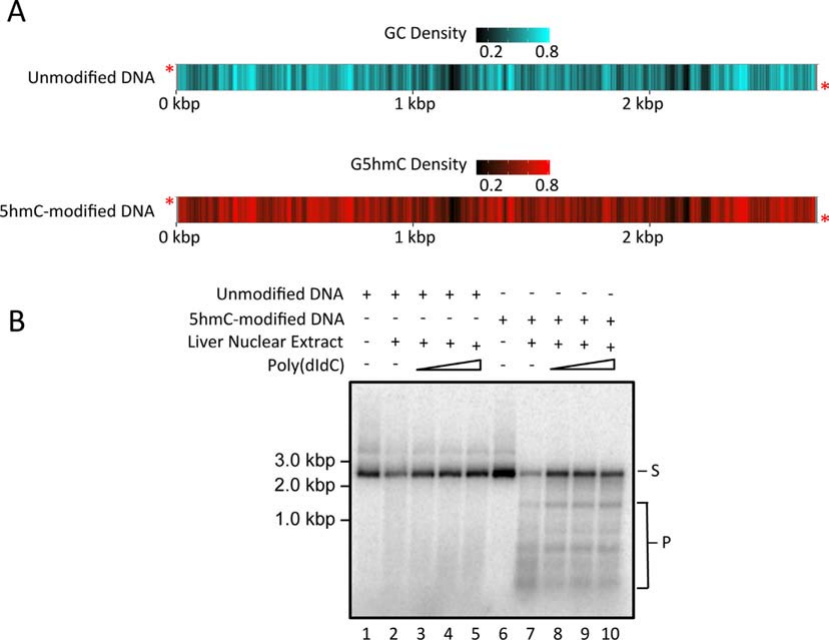
A component of LiNE preferentially cleaves 5hmC-modified DNA. 4.5 ng (2.6 fmol DNA molecules) of either cytosine or the fully 5hmC-modified 2.7 kbp substrates (A) were incubated with 25 ng LiNE with an increasing molar excess (0.1- to 10-fold) of poly(dIdC) (B). *, radioactive label; S, substrate; P, products.

We were interested in determining the effect that mouse LiNE would have on 5hmC-modified DNA. With this idea in mind, we designed two substrates that were PCR products derived from pUC19 (see Supplementary Tables S1 and S2 for substrate and primer sequences; the GC and G5hmC densities of the substrates, averaged over a 20-bp window, are shown in Figure 1A). The first substrate contained unmodified cytosines and the other substrate contained 5hmC in place of all the cytosines. Each of these substrates was incubated with increasing concentrations of purified LiNE (Supplementary Figure S1) and the DNA products were resolved on an agarose gel (Supplementary Figure S3). Surprisingly, in the lanes that contained the 5hmC-modified substrate we observed the appearance of several distinct bands after the incubation with LiNE (Supplementary Figure S3, lanes 6–8). These distinct bands were not present in the reactions that contained the unmodified cytosine substrate incubated with LiNE (Supplementary Figure S3, lanes 2–4).

In addition to the products liberated when the 5hmC-modified substrate was incubated with LiNE, we noted an apparent non-specific nuclease activity present in LiNEs that degraded both cytosine and 5hmC substrates (Supplementary Figure S3, lanes 2 and 6). We titrated increasing concentrations of poly(dIdC) into the cleavage reactions to demonstrate that the endonuclease activity observed preferentially cleaved 5hmC-modified DNA (Figure 1B). We observed that as the amount of poly(dIdC) increased, the non-specific nuclease activity decreased in the cleavage reactions involving both the cytosine and 5hmC substrates. The specific cleavage products created by incubating LiNE with the 5hmC-modified substrate appeared to increase with incrementing amounts of poly(dIdC) (Figure 1B, lanes 8–10). This result suggests that an activity from the LiNE was preferentially catalyzing the formation of sequence-specific double-stranded DNA breaks provided the DNA was cytosine hydroxymethylated.

### Identification of the enzyme that catalyzes the formation of specific cleavage products from 5hmC-modified DNA

**Figure 2.**
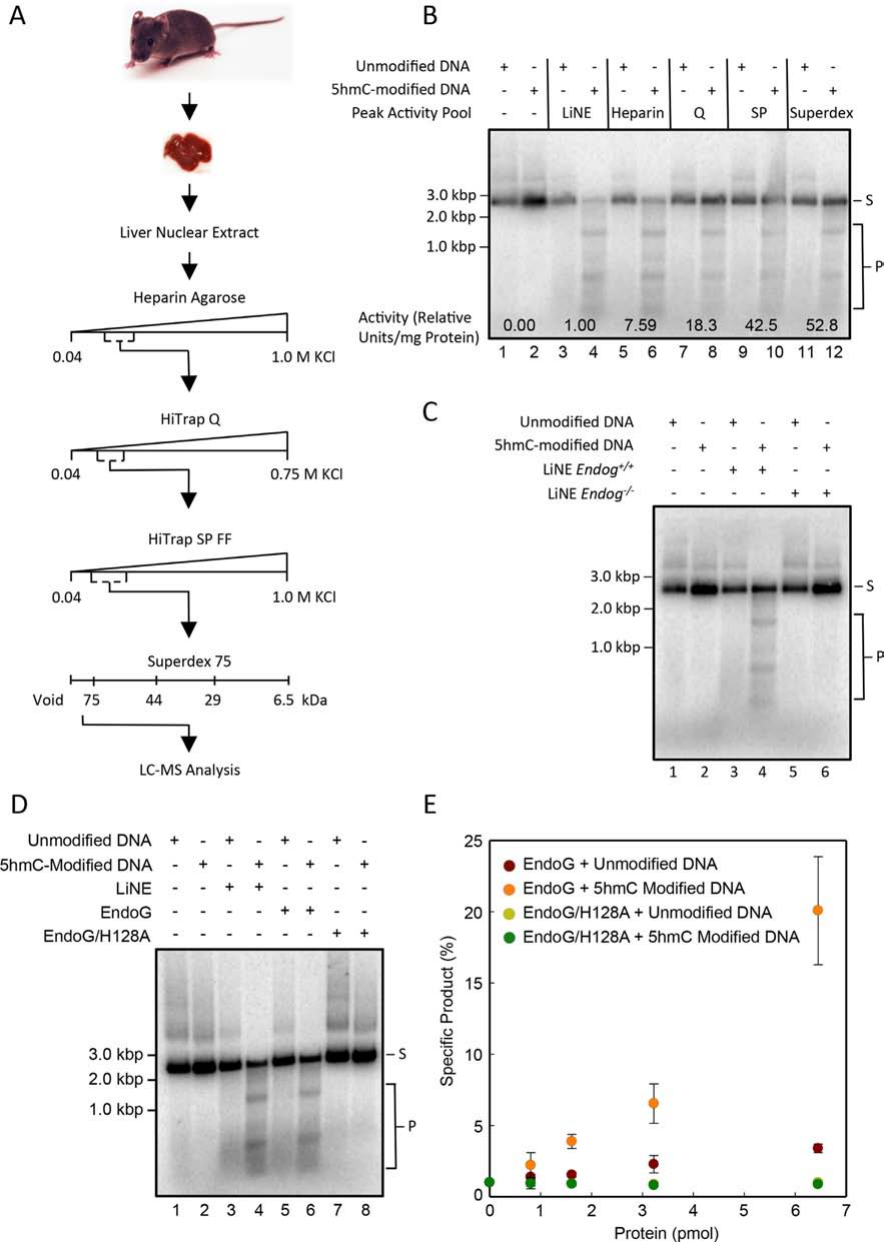
Purification and identification of EndoG that preferentially cleaves 5hmC-modified DNA. Purification scheme, using mouse livers as a starting material, for the endonuclease that preferentially cleaves 5hmC-modified DNA (A). 4.5 ng (2.6 fmol DNA molecules) of either unmodified or 5hmC-modified DNA substrates were incubated with each successive column pool (B). Full column activity fractions are shown in Supplementary Figures S4–S7. 4.5 ng (2.6 fmol DNA molecules) of either cytosine or 5hmC-modified substrates were incubated with 50 ng LiNE from wild-type mice or 50 ng LiNE derived from *Endog^−/−^* mice (C). 50 ng LiNE, 6.5 nM Purified EndoG or 6.5 nM Purified EndoG/H128A were incubated with 4.5 ng (2.6 fmol DNA molecules) unmodified or 5hmC-modified 2.7 kbp DNA substrates (D). Quantification of the specific cleavage products resulting from a titration of EndoG and EndoG/H128A (E). S, substrate; P, products.

We endeavored to identify the protein or protein complex responsible for the cleavage activity observed in Figure 1B. Nuclear extracts from the livers of 54 mice were prepared and fractionated according to the scheme shown in Figure 2A. After each purification step the fractions were assayed for double-stranded 5hmC-modified DNA cleavage activity (Supplementary Figures S4–S7). Fractions from each purification step that contained the greatest double-stranded 5hmC-specific cleavage activity were pooled and subjected to further fractionation (Figure 2B). During purification of the cleavage activity we noted a reduced cleavage specificity; however, we note that at high enzyme concentrations many endonucleases display reduced specificity (41), indeed at the end of the purification we were able to recover the specific cleavage products after incubation with 5hmC-modified DNA (Figure 2B). After the final purification step, proteins were resolved using SDS-PAGE. The entire gel lane was subjected to nanoLC-ESI-MS/MS analysis to identify proteins of interest. The nanoLC-ESI-MS/MS analysis identified 259 proteins (Supplementary Table S3 and Supplementary Figure S8). Several of these proteins contained putative nuclease domains; however, EndoG became a primary candidate because EndoG was the only protein that has been previously demonstrated to function as an endonuclease (42). Therefore, we tested LiNE from *Endog*^−/−^ mice for cleavage activity on both the cytosine and 5hmC-modified substrates. Supplementary Figure S2A and B demonstrate that wild-type and *Endog*^−/−^ LiNE both contain equivalent amounts of protein, measured by western blot, and are equally active, as measured by ATP hydrolysis assays. Interestingly, LiNE lacking EndoG, when incubated with a 5hmC-modified substrate, did not catalyze the formation of specific cleavage products (Figure 2C). This result strongly supports the hypothesis that EndoG is necessary for the observed 5hmC cleavage activity.

### Recombinant EndoG preferentially cleaves 5hmC-modified DNA

We expressed and partially purified recombinant mouse EndoG and a catalytically inactive mutant of EndoG–EndoG/H128A—from *Escherichia coli* (Supplementary Figure S9). The cytosine or the 5hmC-modified substrate was incubated with LiNE, recombinant EndoG or EndoG/H128A. Intriguingly, we noted that recombinant EndoG produced the same cleavage pattern as the LiNE (Figure 2D, lanes 4 and 6 and Supplementary Figure S10). Furthermore, the catalytically inactive EndoG/H128A was unable to cleave the cytosine or the 5hmC-modified substrate (Figure 2D, lanes 7 and 8). The production of specific cleavage products was dependent on EndoG concentration (Figure 2E and Supplementary Figure S10). Elevated EndoG concentrations and long incubation times resulted in non-specific DNA degradation (data not shown). We show that recombinant EndoG does not preferentially shift 5hmC-modified DNA (Supplementary Figure S11). While we cannot rule out the possibility that proteins copurifying with EndoG can shift DNA, this result indicates that EndoG recognizes and binds equally well to the DNA backbone; the catalytic activity is dependent on the recognition of 5hmC-modified DNA. This data, taken together, strongly suggests that EndoG is responsible for the preferential cleavage of 5hmC-modified DNA seen in our LiNE cleavage assays.

### EndoG preferentially cleaves 5hmC-modified DNA within the core sequence 5′-GGGG^5hm^CCAG-3′

**Figure 3.**
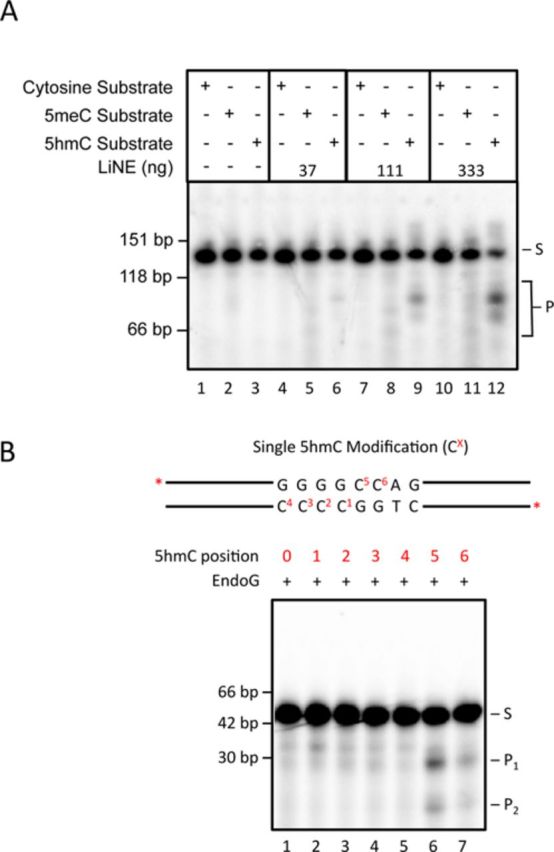
The 5hmC-cleavage activity cannot cleave 5meC modified DNA and EndoG preferentially cleaves the sequence 5′-GGGG^5hm^CCAG-3′. 4.5 ng each short (130 bp) cytosine, fully 5meC and fully 5hmC substrates (5hmC), ^32^P labeled at both ends, were incubated with a titration of LiNE ranging from 37.0 to 333 ng (A). dsOligonucleotides (45 bp) that were singly 5hmC-modified at the indicated position were incubated with purified 12.5 nM EndoG (B). S, substrate; P, products; P_1_, product 1; P_2_, product 2.

Next, we attempted to develop a better understanding of the cleavage specificity of EndoG for 5hmC-modified DNA. We hypothesized that the bands produced from the 5hmC cleavage activity observed in Figure 1B were the result of differently sized bands produced from the cleavage of the 2.7 kbp substrate. Therefore, we cloned and sequenced the larger, ∼1.3 kbp band. The resulting sequence suggested that the preferred EndoG cleavage sequence was 5′-GGGGCCAG-3′/5′-CTGGCCCC-3′. Indeed, mutating all cytosines to thymines in this core sequence resulted in a substrate that could not be cleaved in this region by EndoG (data not shown). Additionally, we were interested in determining if 5meC in the same context would be a substrate for this specific cleavage activity. Therefore, we created shorter unmodified, fully 5meC-modified (100% of cytosines are 5meC) or fully 5hmC-modified (100% of cytosines are 5hmC) substrates and performed an EndoG cleavage assay (Supplementary Figure S12; sequences in Supplementary Table S1). After incubation with LiNE, substrates that were unmodified or fully cytosine methylated did not produce the specific cleavage products that were observed in the fully cytosine hydroxymethylated substrates (Figure 3A, compare lanes 5, 8 and 11 with lanes 6, 9 and 12). This result suggests that the DNA sequence 5′-GGGGCCAG-3′/5′-CTGGCCCC-3′ is preferred by EndoG for cleavage if the cytosines are hydroxymethylated and that cytosine methylation in the same sequence is not preferentially cleaved by EndoG.

We endeavored to determine if a single cytosine within the 5′-GGGGCCAG-3′/5′-CTGGCCCC-3′ sequence, if hydroxymethylated, would be sufficient for preferential cleavage by EndoG. Therefore, we produced six double-stranded oligonucleotide substrates that contained a single 5hmC modification at each of the cytosines in the 5′-CTGGC^1^C^2^C^3^C^4–^3′/5′-GGGGC^5^C^6^AG-3′ sequence, where the superscripted number represents the cytosine that is cytosine hydroxymethylated, and a negative control substrate that contained no cytosine hydroxymethylation (see Figure 3B for schematic). After incubating each of these substrates with EndoG, we observed that the presence of 5hmC in either the fifth or the sixth position is sufficient for recognition and cleavage by EndoG. This EndoG-mediated cleavage produces two specific products (P_1_ and P_2_) of the expected size if the cleavage occurs at the 5′-GGGGCCAG-3′/5′-CTGGCCCC-3′ (Figure 3B, lanes 6 and 7). The substrate that contains a 5hmC at the fifth position appears to be most efficiently cleaved when incubated with EndoG. Indeed, the kinetics of the EndoG- and 5hmC-mediated cleavage suggest that 5hmC at position 5 is strongly preferred as compared with an unmodified substrate (Table 1). Interestingly, when a 5hmC is added at both positions 1 and 5 the cleavage specificity returns to that of unmodified DNA. We then evaluated the cleavage products to determine the type of end produced by EndoG-mediated cleavage—blunt, 5′ overhang or 3′ overhang. This was accomplished by resolving a portion of EndoG cleavage reactions 5 and 6 on denaturing PAGE (Supplementary Figure S13). This resulted in product sizes that are consistent with a 5′-GGCC overhang.

**Table 1.** Kinetic parameters of EndoG for various unmodified and 5hmC modified dsDNA oligonucleotide substrates. Error represents the standard deviation from the mean in three independent experiments. See Figure 3B for substrate schematics.

Substrate	*K*_m_ (nM)	*V*_max_ (nmol * s^−1^)	*K*_cat_ (s^−1^)	*K*_cat_/*K*_m_ (nM^−1^ s^−1^)
Cytosine	11.2 ± 2.29	0.53 ± 0.08	409.9 ± 64.55	36.9 ± 3.17
5hmC pos 5	2.95 ± 1.14	1.99 ± 0.95	1539 ± 731.3	566 ± 263
5hmC pos 1 and 5	10.6 ± 1.41	0.52 ± 0.19	399.6 ± 143.3	38.1 ± 13.8

### EndoG catalyzes cleavage at 5′-GGGGCCAG-3′ sequences in HeLa cells only if the cytosines are hydroxymethylated

**Figure 4.**
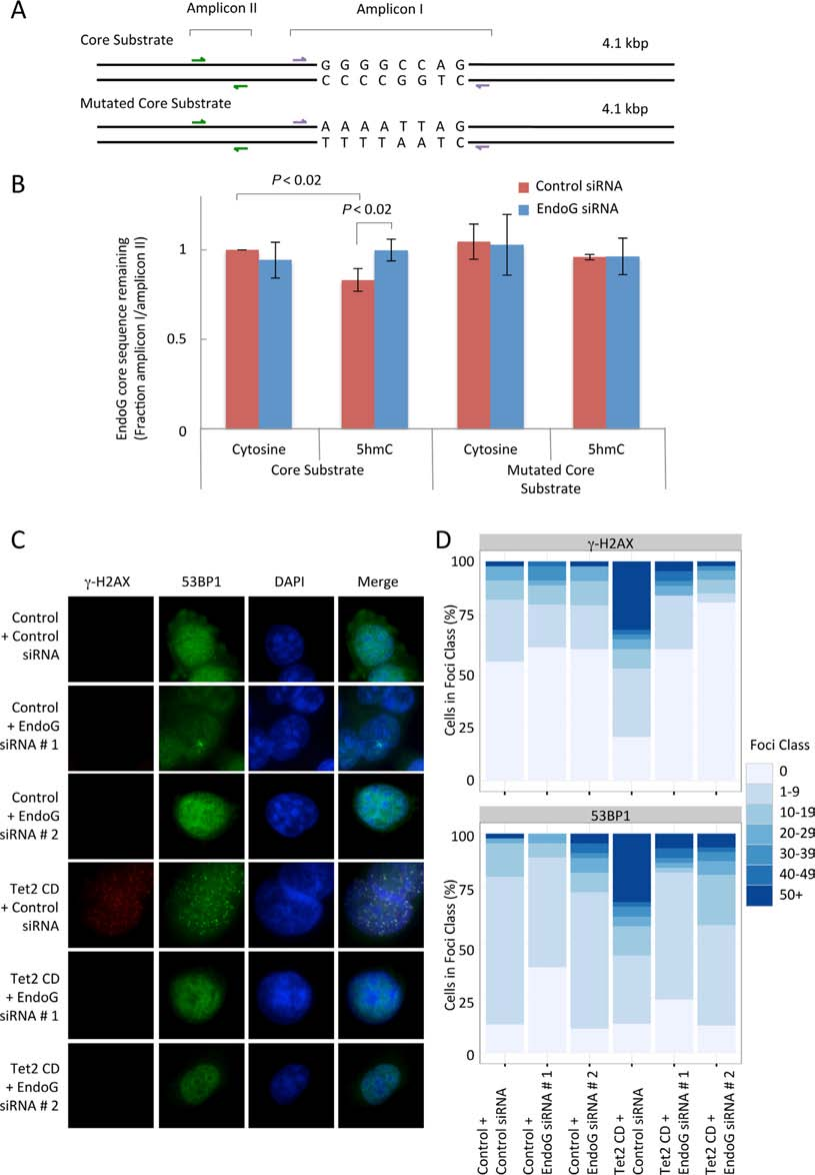
EndoG both cleaves cytosine hydroxymethylated DNA within the sequence context 5′-GGGGCCAG-3′ and promotes the formation of γ-H2AX foci *in vivo*. Schematic (not to scale) of the substrate design for the assay, each 4.1 kbp substrate contained either cytosine or 5hmC in place of all the cytosines. The core substrate contains the EndoG recognition sequence while the core mutated substrate has the EndoG recognition sequence mutated (A). 0.5 μg of each of the four DNA substrates was transfected into HeLa cells that were treated with a control or EndoG siRNA 1. The total DNA was recovered 20 h after transfection. Bars on the graph represent the total amount of the core EndoG recognition region remaining (amplicon I) as a fraction of DNA transfected (amplicon II) quantified by qPCR (B). Data are presented as the mean ± standard deviation, *n* = 7; statistical significance was assigned using a two-tailed Student's *t*-test. Representative images of γ-H2AX foci and 53BP1 foci observed in HeLa cells stably transfected with either a control plasmid or a plasmid that overexpresses the Tet2 CD. Two different siRNAs targeting EndoG were transfected into both the control and the Tet2 CD cell line and incubated for 48 h (C). γ-H2AX foci were counted and grouped according to the number of foci per cell in each of the six combinations of cell lines evaluated (D).

We wanted to demonstrate that EndoG could catalyze the cleavage of substrates containing 5hmC within the core sequence context 5′-GGGGCCAG-3′/5′-CTGGCCCC-3′ *in vivo*. Therefore, we created two 4.1 kbp substrates to be transfected into HeLa cells (Figure 4A; sequences in Supplementary Table S1): one substrate contained the core EndoG recognition sequence—5′-GGGGCCAG-3′/5′-CTGGCCCC-3′ (core substrate)—and the other substrate (mutated core substrate) contained a mutated variant of the core sequence—5′-AAAATTAG-3′/5′-CTAATTTT-3′—that could not be cleaved efficiently by EndoG *in vitro*. These substrates contained two amplicons: amplicon I stretches across the core EndoG cleavage sequence, or the mutated variant of this core sequence; amplicon II is located outside the EndoG core sequence. These substrates were transfected into HeLa cells, cellular DNA was recovered and the DNA cleaved in amplicon I was computed as a function of transfection efficiency (amplicon II). In HeLa cells, the core substrate containing 5hmC showed significantly less signal than the unmodified core substrate at amplicon I (*P* < 0.02), indicating that more cleavage had occurred at the 5hmC-modified substrate (Figure 4B). After EndoG knockdown with siRNA 1 (Supplementary Figure S14) the amount of the 5hmC-modified amplicon I from the core substrate that remains after transfection into HeLa cells is not statistically different from amount of amplicon I remaining in the mutated core substrates. We were unable to detect any reduction in amplicon I when HeLa cells were transfected with the mutated core substrates, which do not contain the EndoG recognition sequence. This data suggests that the 5hmC cleavage activity observed *in vivo* is dependent on EndoG, cytosine hydroxymethylation and the sequence 5′-GGGGCCAG-3′/5′-CTGGCCCC-3′.

### Tet2 catalytic domain overexpression results in the formation of EndoG-dependent γ-H2AX and 53BP1 foci

We were interested in determining if the synthesis of 5hmC would result in an increase in double-stranded breaks *in vivo*, and importantly to determine if this postulated increase in DNA breaks was dependent on EndoG. Therefore, we created four stably transfected HeLa cell lines: a HeLa cell line containing empty vectors (control), HeLa cells expressing the Tet2 CD, HeLa cells expressing a stable shRNA to EndoG and a HeLa cell line overexpressing both the Tet2 CD and a stable shRNA to EndoG. Tet2 CD was chosen because it had been previously described as the most efficient of the Tet enzymes in catalyzing the conversion of 5meC to 5hmC *in vivo* (5). The relevant gene expression characteristics of these cell lines are shown in Supplementary Figure S15A. It is well established that histone H2AX phosphorylated at Serine 139 (γ-H2AX) accumulates at double-stranded DNA breaks (43–45). Therefore, to measure DNA breaks we analyzed the γ-H2AX foci content in each of these modified HeLa cell lines. The control cell line showed γ-H2AX foci (Supplementary Figure S16A and B) levels similar to previous reports (46,47). Irradiated HeLa cells also showed γ-H2AX and 53BP1 foci that were similar to already published data (48) (Supplementary Figure S17). The cell line overexpressing the Tet2 CD displays a dramatic increase in the number of cells with γ-H2AX foci and the number of foci per cell (Supplementary Figure S16A and B). Indicating that these foci are *bona fide* double-stranded DNA breaks, in an independent experiment these γ-H2AX foci co-localize with 53BP1 foci (Figure 4C and D, cell properties shown in Supplementary Figure S18A–C). Interestingly, the number of cells with γ-H2AX foci and the number of foci per cell returned to the control level when the cells are additionally deficient for EndoG (Figure 4C and D and Supplementary Figure S16A and B). These modified stable cell lines grew at similar rates (Supplementary Figure S15B) and similar doubling times (Supplementary Figure S15C), with the Tet2 CD overexpressing HeLa cell growing slightly more slowly and therefore have the longest doubling time. The stable cell lines had similar levels of apoptotic cells (Supplementary Figure S19A and B) and the viability of each of the cell lines was consistently above 95% (data not shown), indicating that these EndoG- and 5hmC-dependent DNA breaks do not kill the cells but are repaired at the expense of doubling time. These results, taken together, suggest that an increase in 5hmC *in vivo* results in dsDNA breaks that are formed in an EndoG-dependent manner.

### EndoG promotes conservative recombination

**Figure 5.**
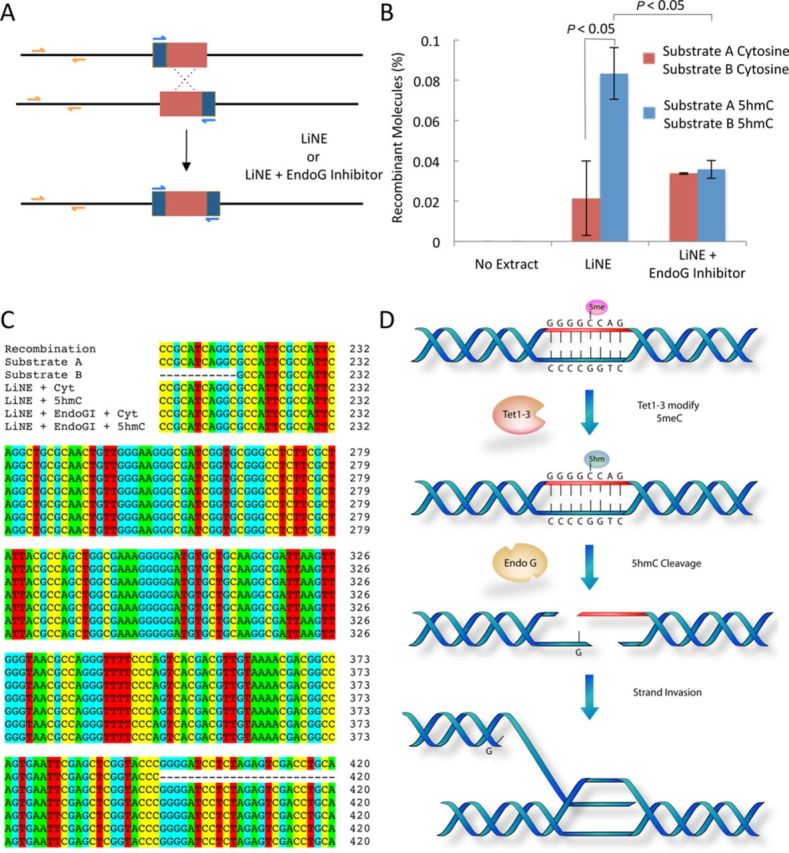
EndoG promotes conservative recombination when the DNA is 5hmC-modified. Substrate A lacks the 3′ region of an amplicon containing the EndoG recognition sequence (blue primers) and Substrate B lacks the 5′ region of this same amplicon. If recombination occurs the complete amplicon is restored resulting in a quantifiable product. Recombination is quantified as the recombination amplicon quantity (blue primers) as a percent of the total DNA quantity recovered (orange amplicon). Substrates A and B were either unmodified or 5hmC-modified at every cytosine residues (A). 100 ng of Substrate A and 100 ng of Substrate B were incubated with 15.3 μg LiNE or μg LiNE and 3.2 nM of a specific inhibitor of EndoG (EndoGI) from *Drosophila melanogaster* as described in the ‘Materials and Methods’ section (B). Data are presented as the mean ± standard deviation, *n* = 3; statistical significance was assigned using a two-tailed Student's *t*-test. Recombinant amplicons from the recombination assays in panel B were cloned and sequenced, representative sequences are shown aligned to the expected sequence for a conservative recombinant molecule (top sequence), the relevant sequence of substrate A (sequence second from top) and the relevant sequence of substrate B (sequence third from top) (C). Full sequences are shown in Supplementary Figure S21. Model for the initiation of recombination at 5′-GGGGCCAG-3′ sequences mediated by EndoG and cytosine hydroxymethylation (D).

We observed that the *in vivo* and *in vitro* cleavage of 5hmC-modified DNA catalyzed by EndoG resulted in the formation of double-stranded DNA breaks. We speculated that these double-stranded DNA breaks would be processed by recombination. We tested this hypothesis by modifying a previously described recombination assay (40). Briefly, two recombination substrates were created. These two substrates are identical, except that Substrate A lacks the 3′ region of a well-defined amplicon and Substrate B lacks the 5′ region of this same amplicon (see Figure 5A for Schematic; Supplementary Table S1 for sequences). The substrates when combined in a qPCR reaction will not produce a product; however, if recombination occurs a qPCR product will be produced (40). We modified this assay such that the substrates were either unmodified or each substrate had every cytosine replaced with 5hmC (100% of cytosines were 5hmC). We incubated substrate A and substrate B with LiNE and in the presence or absence of a specific inhibitor of EndoG (dEndoGI) from *Drosophila melanogaster.* Inhibition of EndoG-mediated cleavage activity is shown in Supplementary Figure S20 and as previously described (49). Unmodified DNA substrates incubated with LiNE produce a low level of recombinant molecules as a percentage of total DNA (Figure 5B). In contrast, 5hmC-modified DNA substrates incubated with LiNE produced 4-fold more recombinant molecules under the same conditions, a statistically significant increase (*P* < 0.05) over the unmodified DNA. Both unmodified and 5hmC-modified DNA incubated with LiNE and dEndoGI result in the formation of a level of recombinant molecules that is not statistically different from the cytosine substrates incubated with LiNE alone. This result shows that (i) the 5hmC-modified DNA is more prone to recombination under the conditions evaluated and (ii) this increase in recombination is dependent on EndoG. Finally, we were interested in determining if the recombinant molecules formed from this assay were conservative recombinant molecules or if these molecules were the result of non-conservative recombination. To address this question we cloned and sequenced each of the qPCR amplicons from the recombination assay performed in Figure 5B. Twelve replicates from each recombination reaction were cloned and sequenced, representative sequences are shown in Figure 5C and in Supplementary Figure S21. In all cases, we observed that the sequence of all 12 replicates resulting recombinant molecules were identical to the expected sequence for a conservative recombinant molecule. These results, taken together, suggest that the LiNE supports conservative recombination and EndoG promotes this type of recombination if the DNA is 5hmC-modified.

## DISCUSSION

Several studies have implicated cytosine hyper-methylation as a driving force for the initiation of endogenous double-stranded breaks (50,51). Interestingly, the techniques used to identify cytosine methylation in these reports—bisulfite sequencing—cannot distinguish between cytosine methylation and cytosine hydroxymethylation (52). Therefore, it is plausible that the endogenous double-stranded breaks correlating with hyper-methylation are to some extent endogenous double-stranded breaks induced by hyper-hydroxymethylation. The presence of an endonuclease that preferentially cleaves 5hmC-modified DNA provides a rationale for the increased amount of double-stranded breaks with increasing cytosine methylation or hydroxymethylation seen previously (32).

When the Tet2 CD is overexpressed in HeLa cells we observed a significant increase in the number of γ-H2AX positive cells and the quantity of γ-H2AX foci per cell, signifying an increase in the number of dsDNA breaks. Importantly, the quantity of γ-H2AX foci returns to near control levels when EndoG is knocked down. The Tet2 CD overexpressing HeLa cell line shows a reduction in growth rate, which was overcome by the additional knockdown of EndoG. This slowed growth rate may be a response to the significant increase in dsDNA breaks. We suggest these breaks must be repaired as apoptosis is not increased and cell viability is not reduced.

Our results suggest that the 5′-GGGG^5hm^CCAG-3′ sequence is preferentially cleaved by EndoG. Although we show that the 5′-GGGG^5hm^CCAG-3′ sequence can be cleaved by EndoG both *in vitro* and *in vivo*, we cannot rule out the possibility that EndoG can catalyze the cleavage of other 5hmC-modified sequences. The characterization of all the EndoG cleavage sequences will certainly be of importance in future studies given that this modification and EndoG appears to initiate recombination. Additionally, this hydroxymethylated cytosine is not in a CpG context; however, we note that this 5hmC at this position is within the CHH (H = A, C or T) sequence context, which is a known target of cytosine methylation (21,53). The cytosine in this sequence context is likely to be hydroxymethylated at some developmental stage *in vivo* and is, therefore, a biologically relevant sequence context. Interestingly, the positioning and number of 5hmC modifications within the 5′-GGGGCCAG-3′ sequence context appear to be important for cleavage by EndoG; substrates modified at the 5 position are cleaved over 15 times more specifically than unmodified substrates, while substrates modified at positions 1 and 5 are cleaved similar to cytosine alone. Our recombination assay substrates shown in Figure 5A contain a fully 5hmC-modified 5′-GGGGCCAG-3′ sequence outside the recombination amplicon. Therefore, the recombination observed within this region is likely the result of (i) cleavage mediated by EndoG at an alternative sequence or (ii) cleavage catalyzed by EndoG at the 5′-GGGGCCAG-3′ sequence outside the recombination amplicon followed by branch migration into and across the recombination amplicon resulting in the conservative recombination products observed.

We (11,12) and others (13,22) have envisaged a role for 5hmC in transcriptional regulation. Others have demonstrated that 5hmC has a role in stem cell pluripotency (54) and in the oxidative demethylation of 5meC (15–17). We do not believe that these studies conflict with the presence of an endonuclease that preferentially cleaves 5hmC-modified DNA. Indeed, cytosine methylation has been demonstrated to have several cellular functions, likewise cytosine hydroxymethylation potentially has multiple functions, including the initiation of recombination.

Previous reports (28,32,34) demonstrate that EndoG creates double-stranded breaks at CG-rich regions; however, cytosine hydroxymethylation has not been implicated as a requirement for this cleavage. While we see EndoG-mediated cleavage at CG-rich regions, EndoG cleaves 5hmC-modified DNA much more efficiently. EndoG resides primarily in the mitochondrial inner membrane space (30) and in the nucleus at a lower concentration (37). Importantly, the LiNE used for this study were free of any detectable mitochondrial contaminants (Supplementary Figure S1). EndoG is thought to induce apoptosis in a caspase-independent manner (34,55–56). It has also been shown that EndoG can generate primers necessary for mitochondrial DNA replication (57). Nevertheless, an *Endog*-deficient (*Endog^−/−^*) mouse did not show apoptotic defects and did not show any deficiencies consistent with ineffective or inefficient mitochondrial DNA replication (35,36). The absence of these defects in the *Endog^−/-^* mice suggests an alternative role for EndoG in nucleic acid metabolism. Indeed, several studies have shown that EndoG is necessary for the initiation of recombination by the creation of double-stranded breaks (31,32). Interestingly, in addition to initiating homologous recombination, EndoG appears to initiate class switch recombination in specialized immune cells (37). A more recent report indicates that nuclear EndoG initiates recombination at the MLL breakpoint (33). Taken together, these reports and the data in our study, showing that EndoG prefers to cleave 5hmC-modified DNA and can promote recombination, strongly suggest that EndoG can initiate recombination at hydroxymethylated cytosine residues. Furthermore, a recent report demonstrates that 5hmC is located at recombinational hotspots, suggesting a role for 5hmC-modified DNA in recombination (23). Several reports also suggest that regions of high GC content are recombinational hotspots (25,58). Therefore, we suggest that EndoG may be responsible for the initiation of an uncharacterized mode of recombination (Figure 5D). In this model EndoG would preferentially initiate recombination by creating double-stranded breaks at specific DNA sites that are cytosine hydroxymethylated. The 5hmC-specific endonuclease activity catalyzed by EndoG is likely to have at least two levels of regulation preventing unintended double-stranded breaks within cells. The first level of EndoG regulation would be accomplished by regulating the cytosine hydroxymethylation status of the sequence to be cleaved, regulated by the Tet1–3 enzymes. The second level of regulation, by a protein or protein complex or by exclusion from the nucleus, would directly modulate the ability of EndoG to access 5hmC-modified DNA.

## SUPPLEMENTARY DATA

Supplementary Data are available at NAR Online.

SUPPLEMENTARY DATA
