# Supplementary material for: Endonuclease G preferentially cleaves 5-hydroxymethylcytosine-modified DNA creating a substrate for recombination
Source: Nucleic Acids Res. 2014 Oct 29;42(21):13280–93. doi: 10.1093/nar/gku1032 (PMC4245937; doi:10.1093/nar/gku1032)
Supplement: SUPPLEMENTARY DATA [file supp_42_21_13280__index.html]

Endonuclease G preferentially cleaves 5-hydroxymethylcytosine-modified DNA creating a substrate for recombination — SUPPLEMENTARY DATA 

# Endonuclease G preferentially cleaves 5-hydroxymethylcytosine-modified DNA creating a substrate for recombination

## SUPPLEMENTARY DATA

**Files in this Data Supplement:**

- SUPPLEMENTARY DATA
