# Supplementary material for: Endonuclease G preferentially cleaves 5-hydroxymethylcytosine-modified DNA creating a substrate for recombination
Source: Nucleic Acids Res. 2014 Oct 29;42(21):13280–93. doi: 10.1093/nar/gku1032 (PMC4245937; doi:10.1093/nar/gku1032)
Supplement: SUPPLEMENTARY DATA [file supp_gku1032_nar-02470-f-2014-File008.pdf]

Supporting Material

Supplemental Figure Legends

Supplemental Figure S1. Liver nuclear extracts are not contaminated with detectable levels of mitochondrial proteins. An increasing titration of 25, 50 or 100 μg of whole liver extract or liver nuclear extracts were resolved on SDS-PAGE followed by Western blotting. Blots were probed with antibodies directed against Mitochondrial Complex IV, Endonuclease G or histone H3.

Supplemental Figure S2. Loading control and activity assay of *Endog*$^{+/+}$ and *Endog*$^{-/-}$ LiNE. 5 ug of LiNE derived from *Endog*$^{+/+}$ or *Endog*$^{-/-}$ were resolved on 12% SDS-PAGE followed by Western blotting. Blots were probed with anti α-Actin Antiserum (A). Quantification of ATP hydrolysis activity assay of *Endog*$^{+/+}$ and *Endog*$^{-/-}$ LiNE, showing that both extracts are equally active (B).

Supplemental Figure S3. An activity from liver nuclear extracts preferentially cleaves 5hmC-modified DNA. 4.5 ng of either unmodified or fully 5hmC-modified 2.7 kbp substrates DNA were incubated with increasing amounts of liver nuclear extracts ranging from 25 ng to 100 ng.

Supplemental Figure S4. Heparin agarose chromatography. The nuclear extracts from 54 mice were processed and applied to a heparin agarose column. Fractions were assayed for their ability to cleave cytosine and 5-hydroxymethylcytosine containing DNA. Fractions containing the greatest amount of cleavage activity (fractions eluting between 50 and 120 mM KCl) were pooled and subjected to further purification (see also Figure 2B and Supplemental Figures S5, S6 and S7). Abbreviations: load (L) and flow-through (FT).

Supplemental Figure S5. Anion exchange chromatography. The heparin agarose pool from Supplemental Figure S4 was applied to a HiTrap Q column. Fractions were assayed for their ability to cleave cytosine and 5-hydroxymethylcytosine containing DNA. Fractions containing the greatest amount of cleavage activity (fractions eluting between 50 and 100 mM KCl) were pooled and subjected to further purification (See also Figure 2B and Supplemental Figures S4, S6 and S7). Abbreviations: load (L) and flow-through (FT).

Supplemental Figure S6. Cation exchange chromatography. The HiTrap Q pool from Supplemental figure S5 was applied to a HiTrap SP column. Fractions were assayed for their ability to cleave cytosine and 5-hydroxymethylcytosine containing DNA. Fractions containing the greatest amount of cleavage activity (fractions eluting between 60 and 80 mM KCl) were pooled and subjected to further purification (see also Figure 2B and Supplemental Figures S4, S5 and S7). Abbreviations: load (L) and flow-through (FT).

Supplemental Figure S7. Size exclusion chromatography. The HiTrap SP pool from Supplemental figure S6 was applied to a Superdex 75 column. Fractions were assayed for their ability to cleave cytosine and 5-hydroxymethylcytosine containing DNA. Fractions containing the greatest amount of cleavage activity (fractions eluting at the apparent molecular weight between 80 and 100 kDa) were pooled and subjected to further purification (see also Figure 2B and Supplemental Figures S4, S5 and S6). Abbreviations: load (L) and flow-through (FT).

Supplemental Figure S8. Identification of endonuclease G using nanoLC-ESI-MS/MS. The full length primary sequence of Endonuclease G is shown. Red indicates peptides identified using LC-MS/MS that map uniquely to the Endonuclease G primary sequence.

Supplemental Figure S9. Partial purifications of EndoG and EndoG/H128A. 6xhis tagged recombinant EndoG and EndoG/H128A were resolved on SDS-PAGE and either Coomassie stained or Western blotted and probed with 6xHis antiserum. 3 μg load for Coomassie staining 500 ng load for Western Analysis.

Supplemental Figure S10. Endonuclease G preferentially catalyzes the cleavage of 5-hydroxymethylcytosine containing DNA. Cytosine and 5-hydroxymethylcytosine containing substrates were incubated with 10 ng liver nuclear extracts or a titration from 0.4 to 12.9 nM of recombinant Endonuclease G.

Supplemental Figure S11. Endonuclease G does not bind preferentially to 5hmC-modified DNA. 4.5 ng (53.3 fmol DNA molecules) each short (130 bp) cytosine, fully 5-methylcytosine (5meC) and fully 5hmC substrates (5hmC), ^{32}P labelled at both ends, were incubated in the presence of 5 mM EDTA at 4°C with an increasing titration (0.33 μM to 1.3 μM) of recombinant Endonuclease G. Reactions were resolved on 6% Native-PAGE.

Supplemental Figure S12. Schematic of the 130 bp substrate. The region suspected to be cleaved from the initial substrate by the liver nuclear extract was cloned into pCR 2.1 Topo. This region was then amplified such that a 130 bp fragment was created with either all cytosine, all 5meC residues or all 5hmC residues. This amplicon was also used to measure cleavage activity in HeLa cells (Schematic Figure 4A and assay shown Figure 4B).

Supplemental Figure S13. Endonuclease G cleaves 5′-GGGGCCAG-3′ sequences leaving a 5′-overhang. Oligonucleotide products from *Hae*III or EndoG digestion were resolved using 20% denaturing PAGE. Reactions with 5hmC modified oligonucleotides at positions 5 and 6 (Figure 3B) yield two primary products when incubated with EndoG – 27 and 14 bases (Lanes 2 and 3). *Hae*III digestion of the same substrates results in a blunt cut at

the 5′-GGCC-3′ sequence resulting in a 29 base fragment and a 16 base fragment (Lane 4). These products suggest that EndoG cleaves the substrate producing a 5′-GGCC overhang.

Supplemental Figure S14. Endonuclease G knockdown over time. HeLa Cells were transfected with control siRNA or EndoG siRNA 1 at a concentration of 30 nM. Knockdown was calculated as percent EndoG RNA remaining compared to untreated HeLa cells.

Supplemental Figure S15. Properties of stably transfected HeLa cell lines. Expression of Expression of Tet2 CD and EndoG (A), Growth curves (B) and doubling times (C) of HeLa cells transfected with control plasmids (control), a plasmid overexpressing the Tet2 catalytic domain (Tet2 CD), a plasmid with an shRNA targeting EndoG and a HeLa cells stably expressing the Tet2 CD and shRNA targeting EndoG.

Supplemental Figure S16. γ-H2AX foci form in an EndoG and 5hmC dependent manner. Representative images of γ-H2AX foci observed in HeLa cells stably transfected with control plasmids, overexpression of Tet2 Catalytic Domain (Tet2 CD), EndoG shRNA or a combination of Tet2 CD overexpression and EndoG shRNA (A). γ-H2AX foci were counted and grouped according to the number of foci per cell in each of the four stably transfected HeLa cell lines, in duplicate (B).

Supplemental Figure S17. γ-H2AX and 53BP1 foci form in response to ionizing radiation. Representative images of γ-H2AX and 53BP1 foci observed in HeLa cells exposed to 1 and 3 Gy of ionizing radiation (Left). γ-H2AX and 53BP1 foci were counted and grouped according to the number of foci per cell (Right).

Supplemental Figure S18. Properties of stable cell lines from Figure 4C.

Quantification of 5hmC content (A), Tet2 expression (B), and EndoG expression – siRNA 1 is identical to the siRNA used Supplemental Figure S14 (C).

Supplemental Figure S19. The stably transfected HeLa cell lines are not more prone to undergo apoptosis. Representative images of TUNEL assays performed on the HeLa cells transfected with control plasmids (control), a plasmid overexpressing the Tet2 catalytic domain (Tet2 CD), a plasmid with an shRNA targeting EndoG and a HeLa cells stably expressing the Tet2 CD and shRNA targeting EndoG (A). Quantification of TUNEL positive cells as a percent of the total cells (B).

Supplemental Figure S20. The EndoG inhibitor from *Drosophila melanogaster* can efficiently inhibit mouse Endonuclease G. The inhibitor of EndoG from *Drosophila melanogaster* was cloned and purified to near homogeneity from *E. coli* (A). Cytosine or 5hmC-modified substrates were treated with EndoG or EndoG the *Drosophlia* inhibitor of EndoG (dEndoG) (B).

Supplemental Figure S21. Recombination enhanced by EndoG generates error free products. Recombinant amplicons from Figure 4B were cloned and sequenced, representative sequences are shown aligned to the expected sequence for a conservative recombinant molecule (top sequence), the relevant sequence of substrate A (sequence second from top) and the relevant sequence of substrate B (sequence third from top).

Supplemental Tables

Supplemental Table S1. Substrate Design

Supplemental Table S2. Oligonucleotides Used

Supplemental Table S3. Proteins Co-purifying with the Endonuclease Activity Observed in Figure 2

Supplemental Figure S1

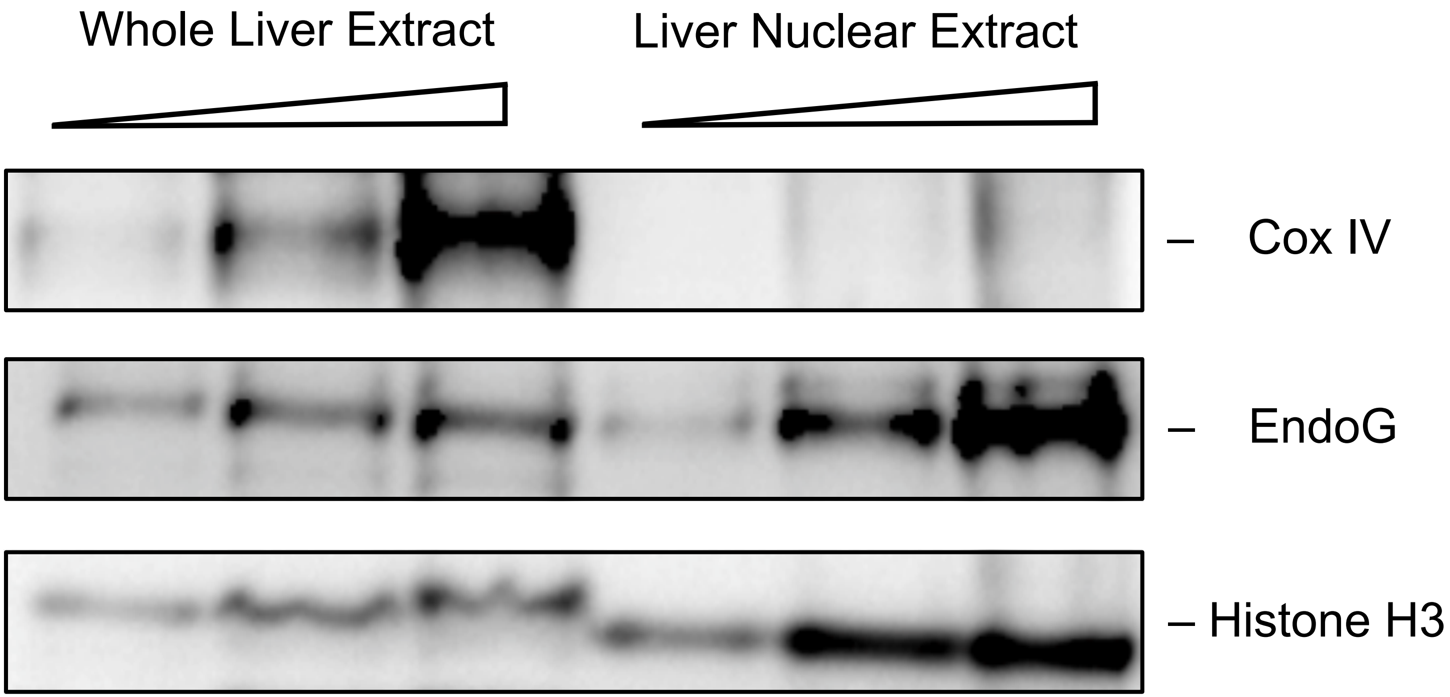

Supplemental Figure S2

A

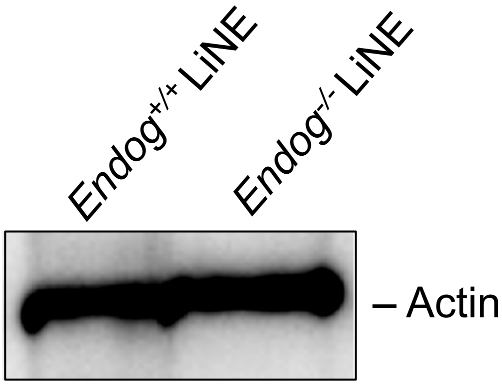

B

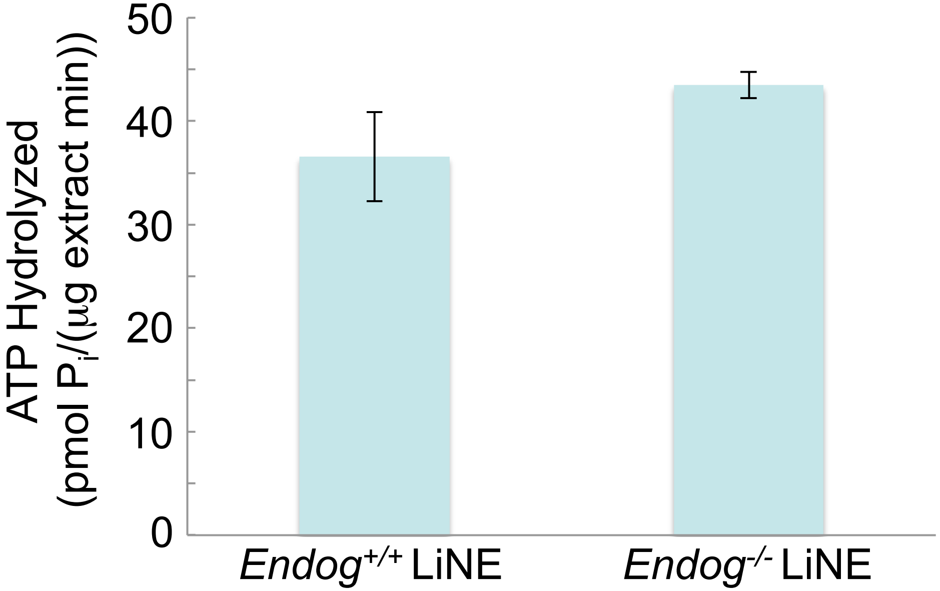

Supplemental Figure S3

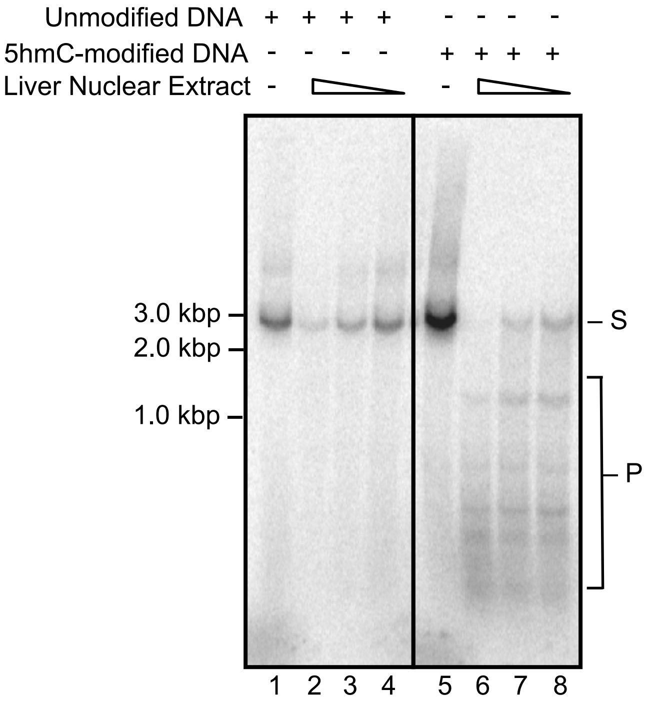

Supplemental Figure S4

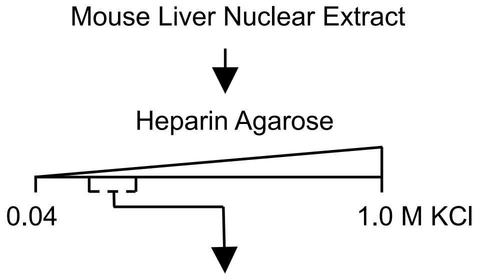

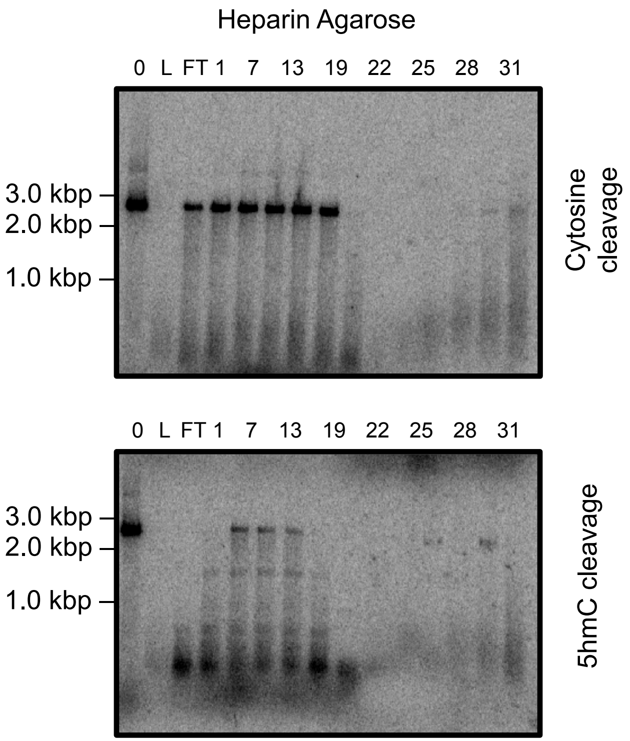

Supplemental Figure S5

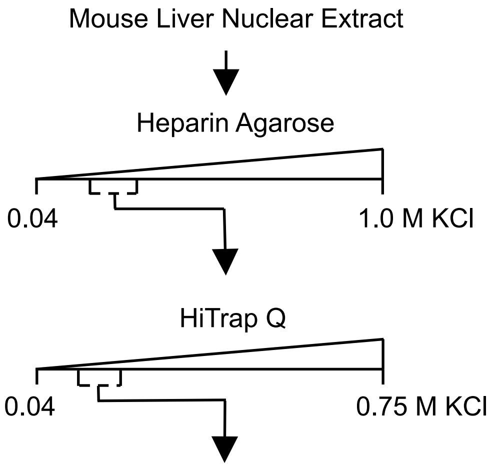

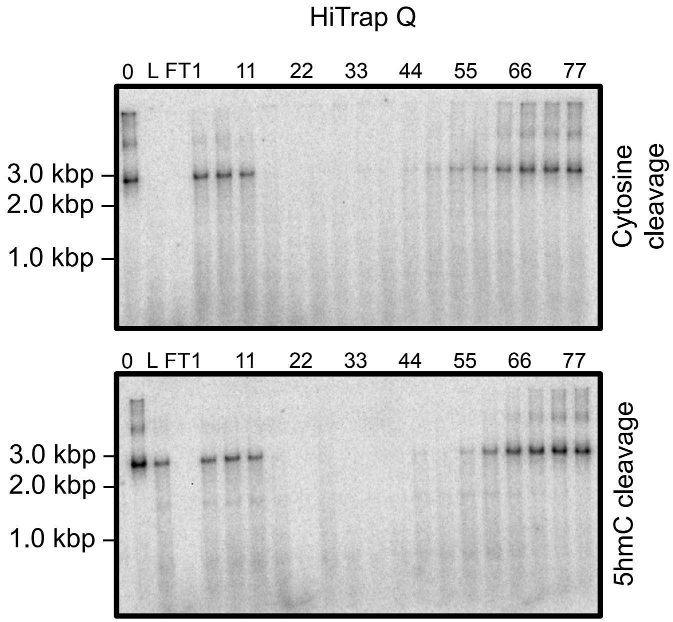

Supplemental Figure S6

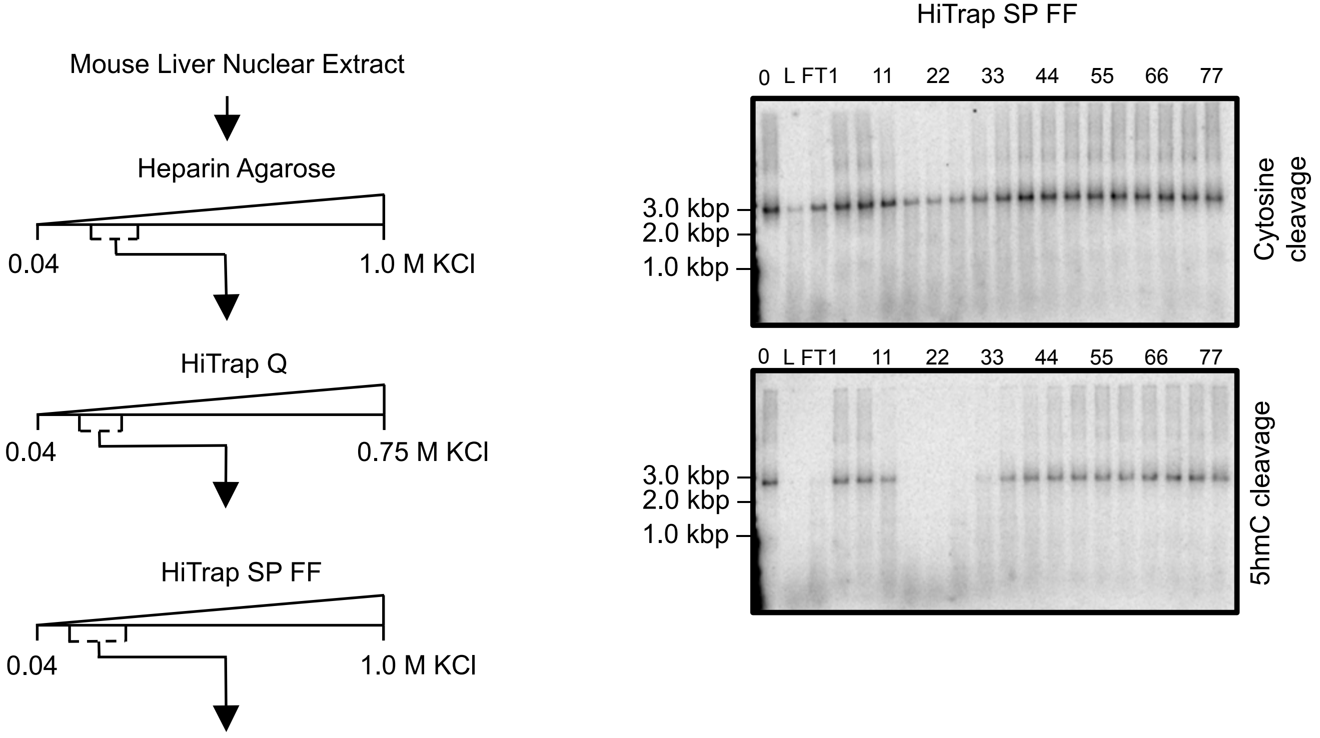

Supplemental Figure S7

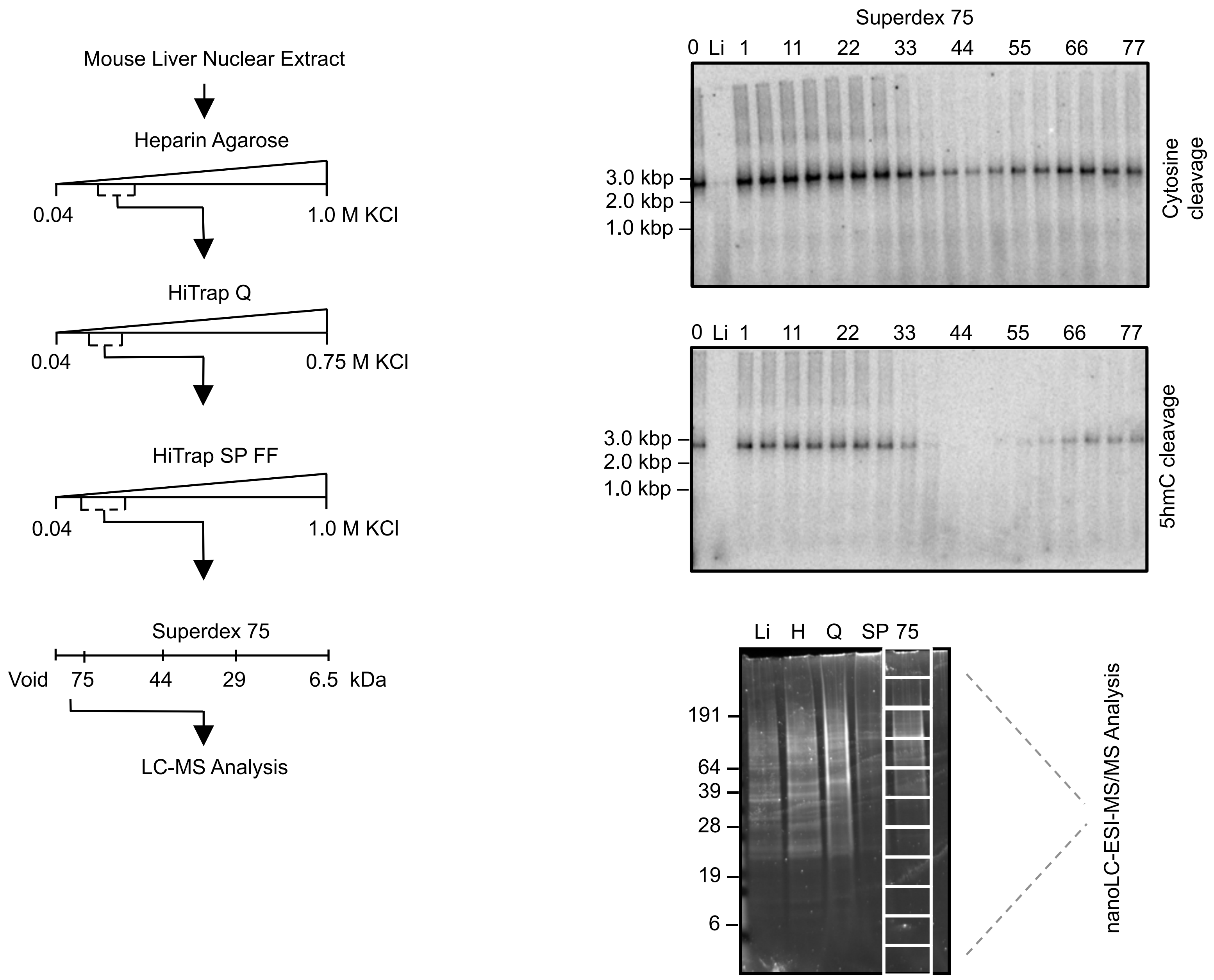

Supplemental Figure S8

```
MRALRAGLTL ALGAGLGAAA EHWRRREGKA PGLLGRVPLL
PVVAADLPAL PGGPAGGTGE LAKYGLPGVA QLRSRESYVL
SYDPRTRGAL WVLEQLRPER LRGDGDRSAC DFREDDSVHA
YHRATNADYR GSGFDRGHLA AAANHRWSQR AMDDTFYLSN
VAPQVPHLNQ NAWNNLERYS RSLTRTYQNV YVCTGPLFLP
RTEADGKSYV KYQVIGKNHV AVPTHFFKVL ILEAAGGQIE
LRSYVMPNAP VDETIPLERF LVPIESIERA SGLLFVPNIL
ARAGNLKAIT AGSK
```

Supplemental Figure S9

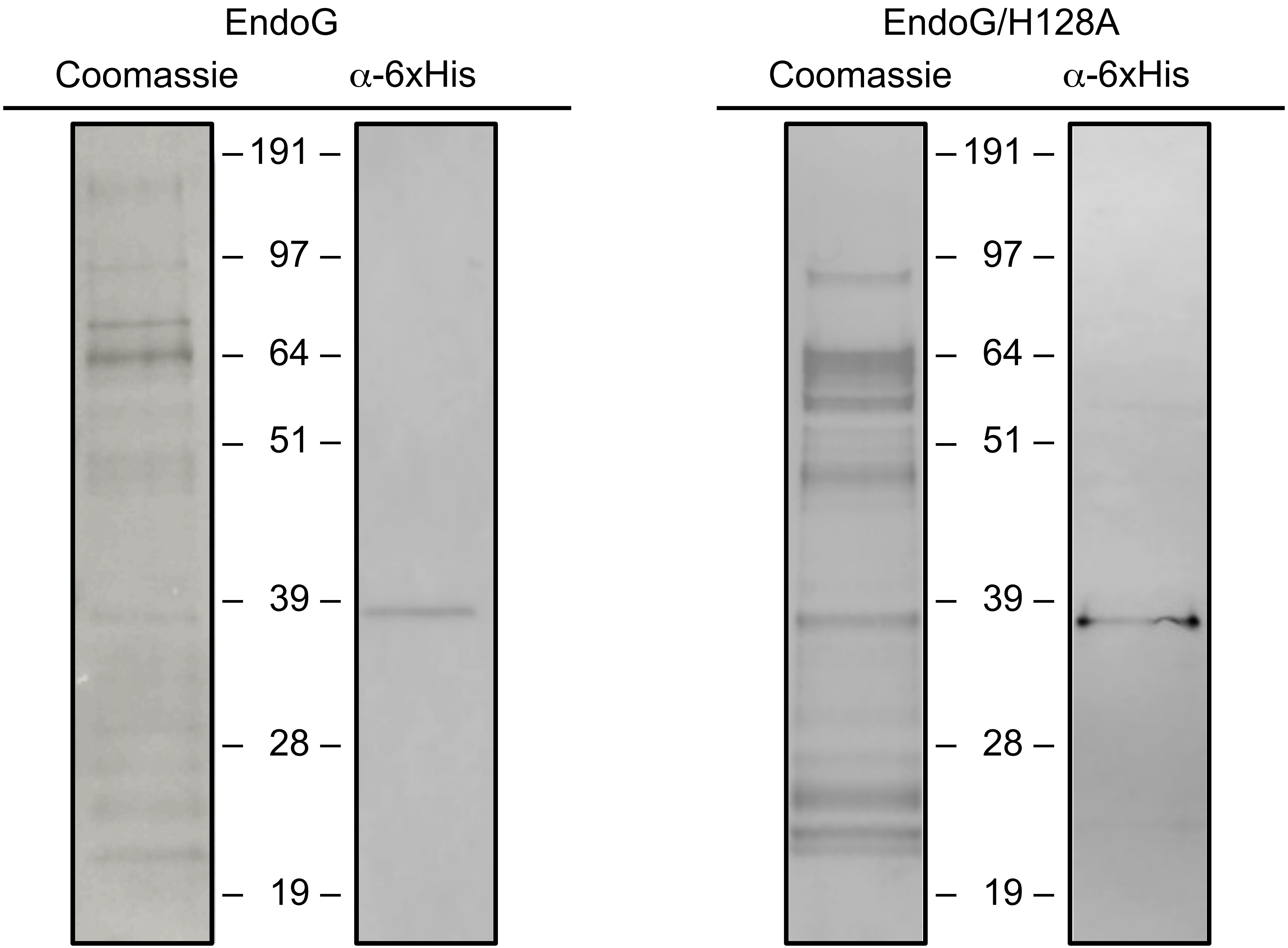

Supplemental Figure S10

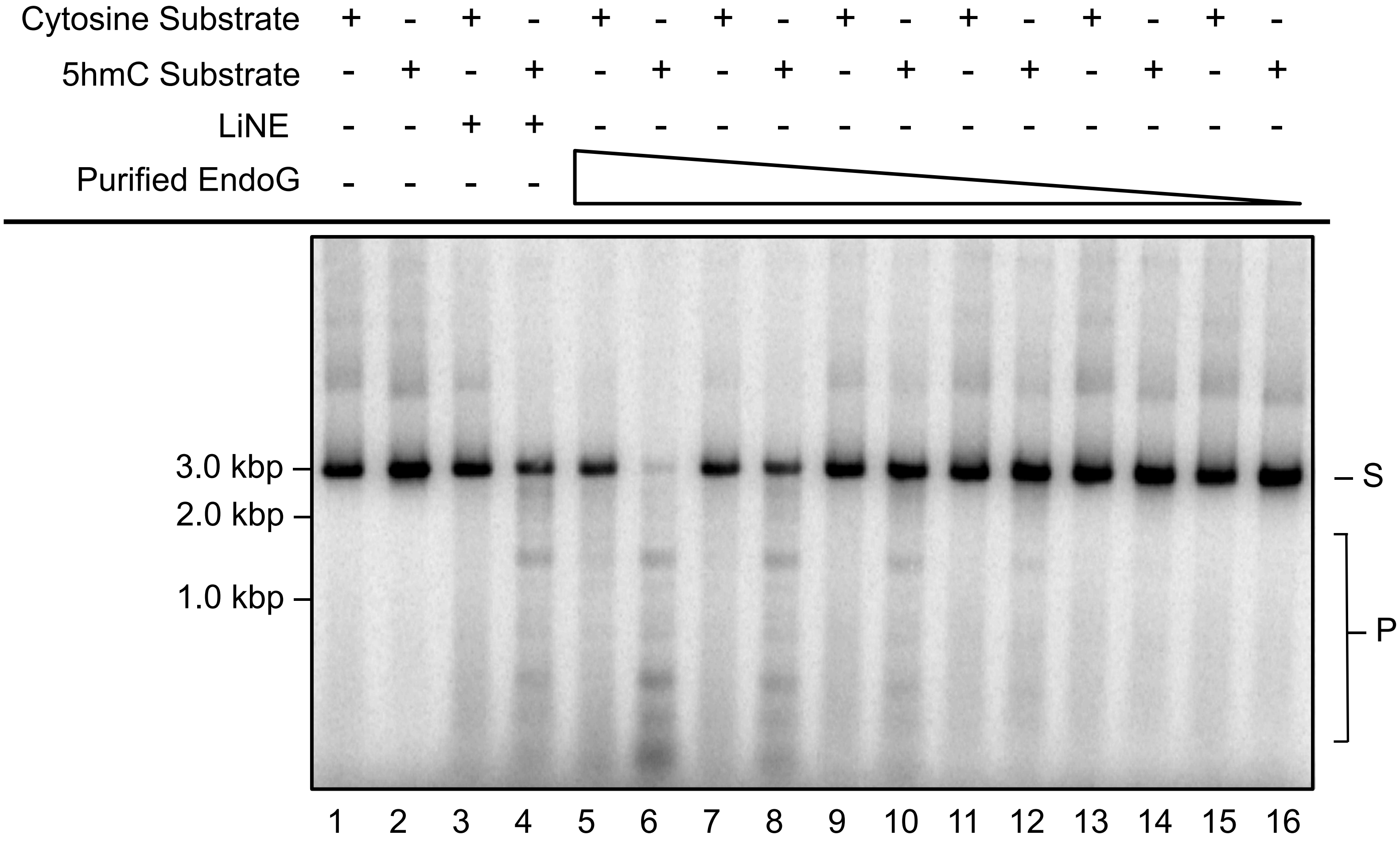

Supplemental Figure S11

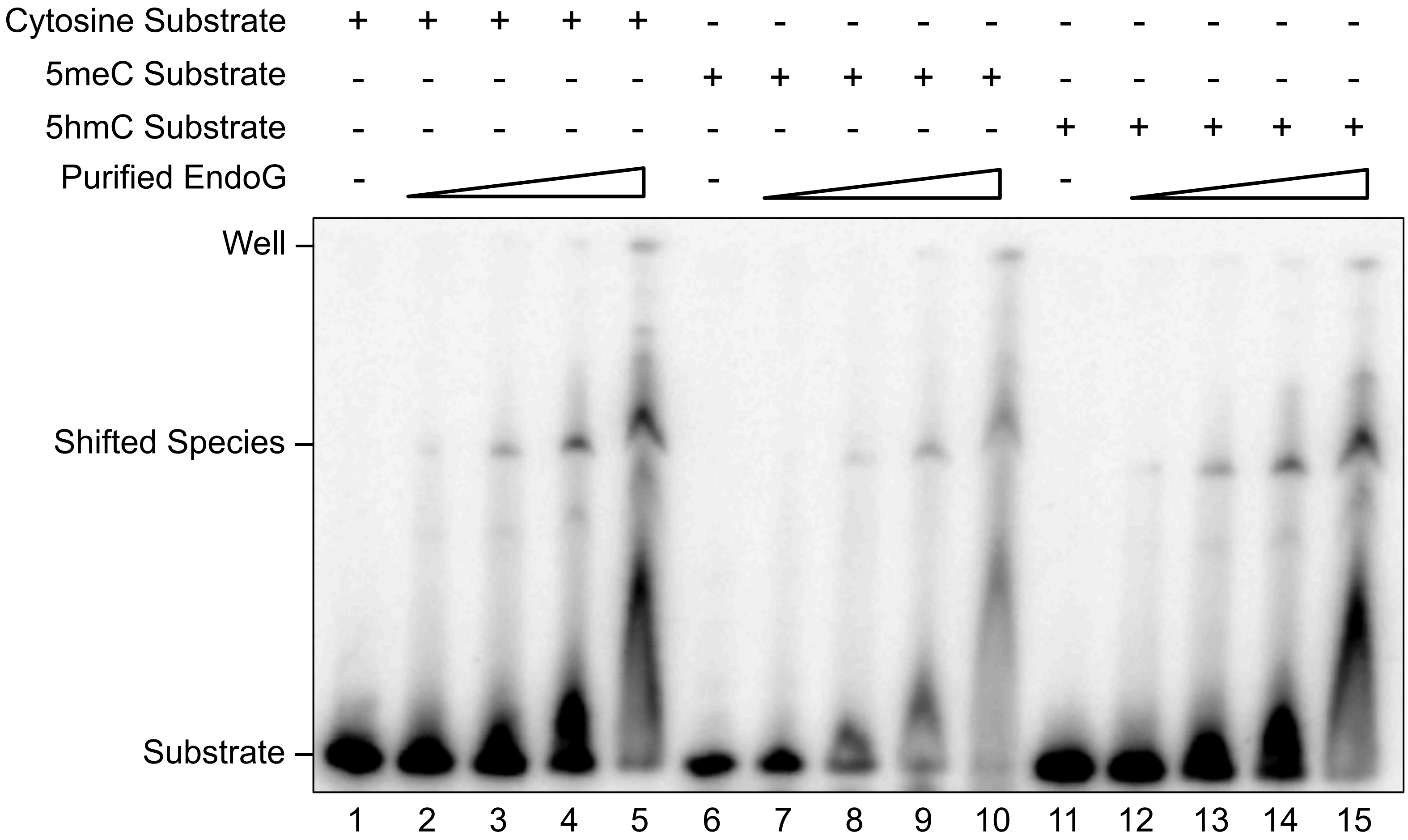

Supplemental Figure S12

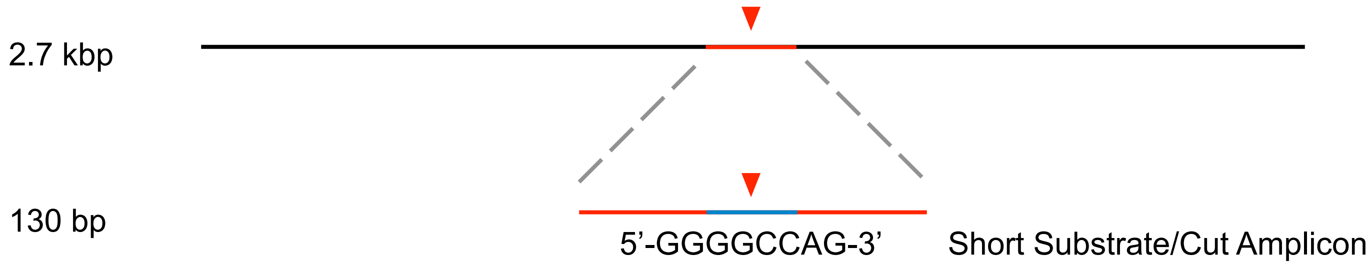

Supplemental Figure S13

1	2	3	4	
+	-	-	+	Unmodified dsOligonucleotide
-	+	-	-	5hmC dsOligonucleotide 5
-	-	+	-	5hmC dsOligonucleotide 6
-	+	+	-	EndoG
-	-	-	+	*Hae*III

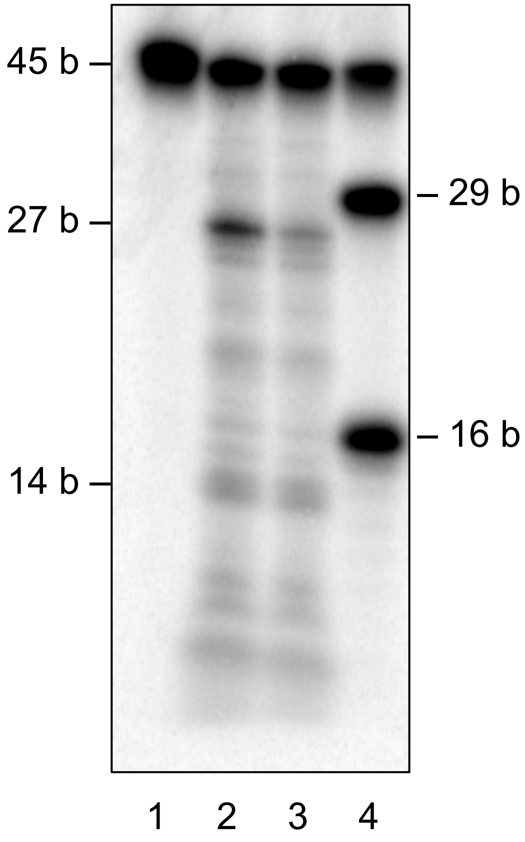

Unmodified dsOligonucleotide digested with *Hae*III

29 b

5′-*GGTCTCGCGGTATCATTGCAGCACTGGGG↓CCAGATGGTAAGCCCT -3′

3′- CCAGAGCGCCATAGTAACGTCGTGACCCC↑GGTCTACCATTCGGGA*-5′

16 b

5hmC dsOligonucleotide 5 digested with EndoG

27 b

5′-*GGTCTCGCGGTATCATTGCAGCACTGG↓GG^{5hm}CC AGATGGTAAGCCCT -3′

3′- CCAGAGCGCCATAGTAACGTCGTGACC CC GG↑TCTACCATTCGGGA*-5′

14 b

5hmC dsOligonucleotide 6 Digested with EndoG

27 b

5′-*GGTCTCGCGGTATCATTGCAGCACTGG↓GGC^{5hm}CAGATGGTAAGCCCT -3′

3′- CCAGAGCGCCATAGTAACGTCGTGACC CCG G↑TCTACCATTCGGGA*-5′

14 b

Supplemental Figure S14

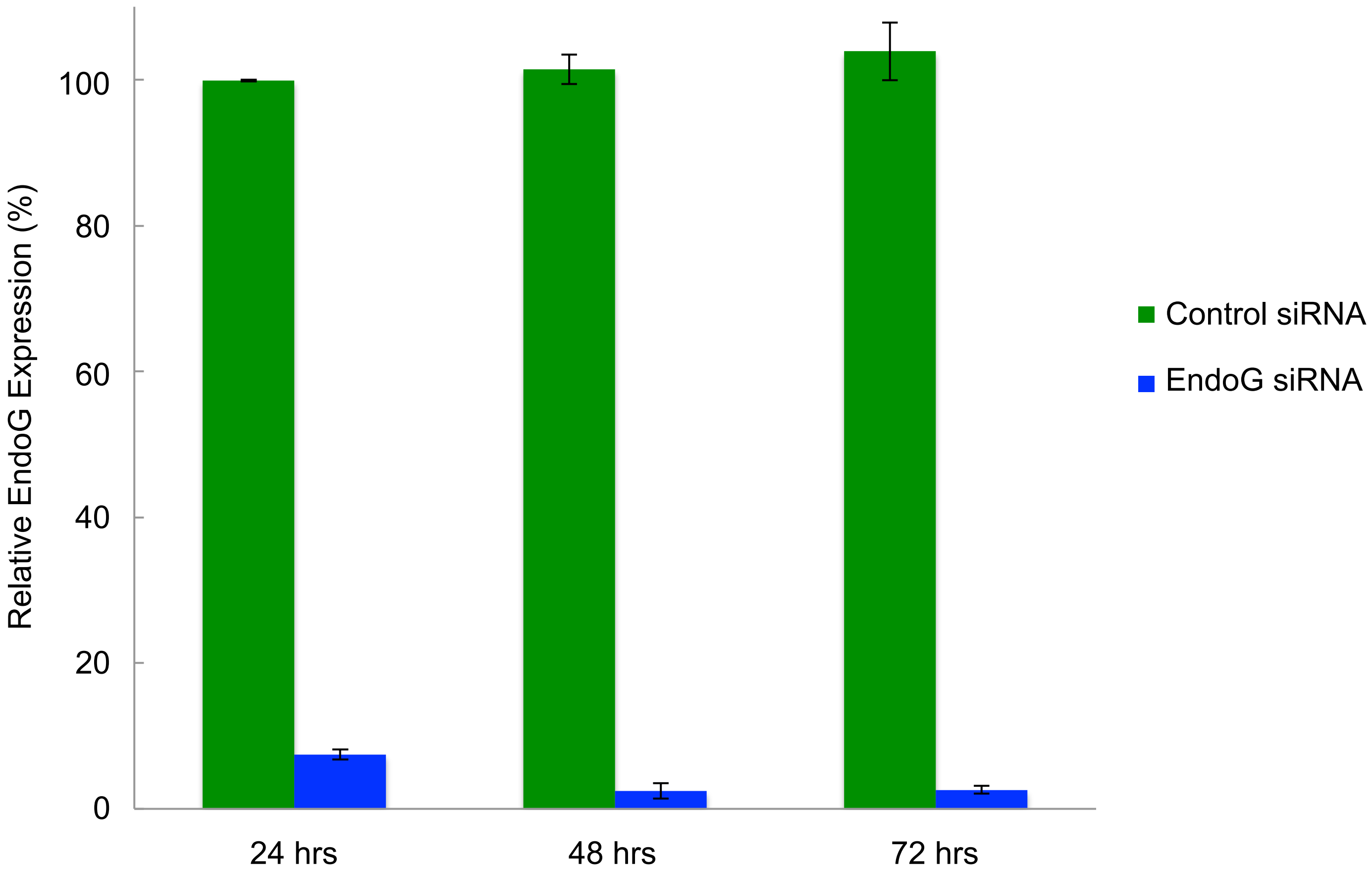

Supplemental Figure S15

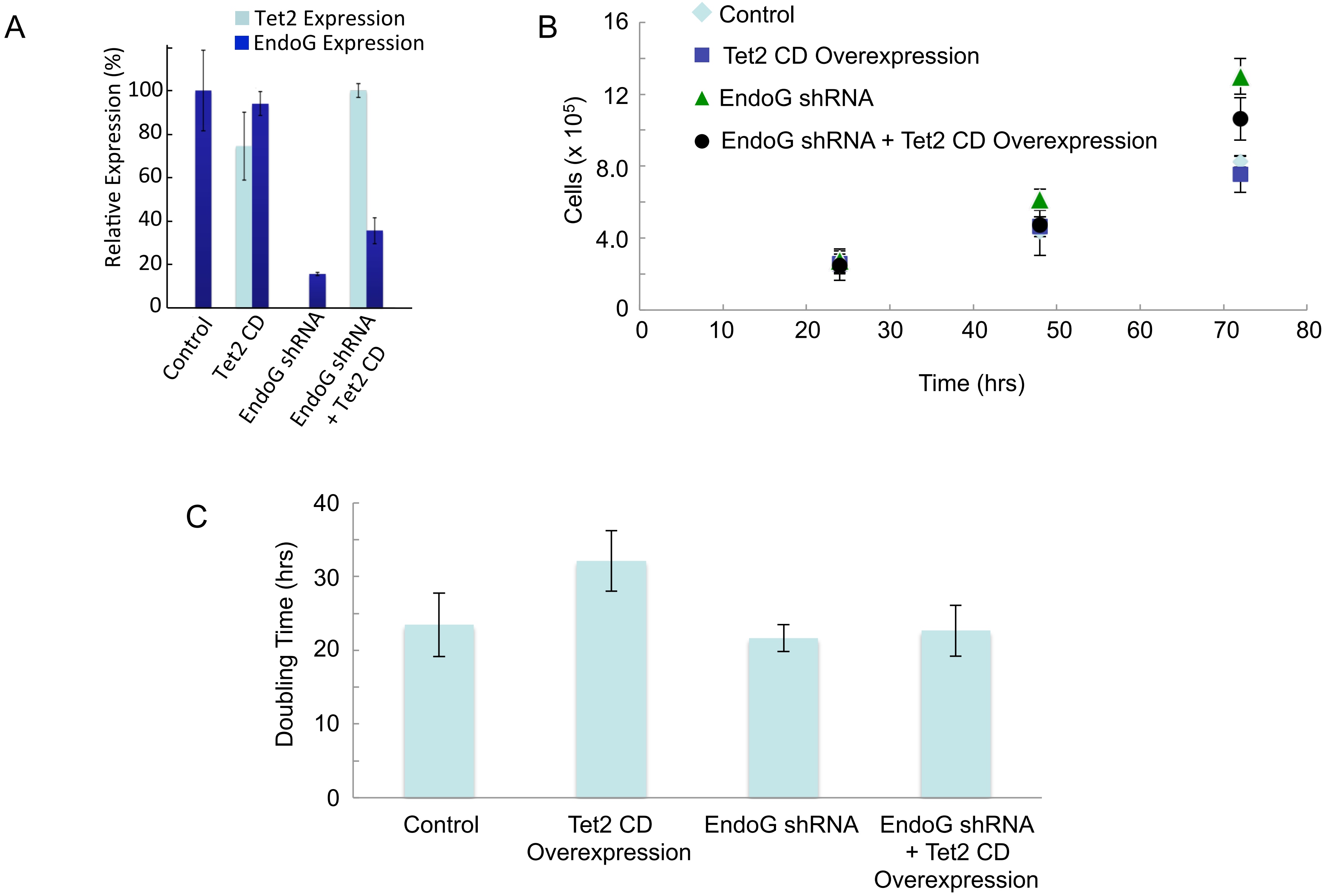

Supplemental Figure S16

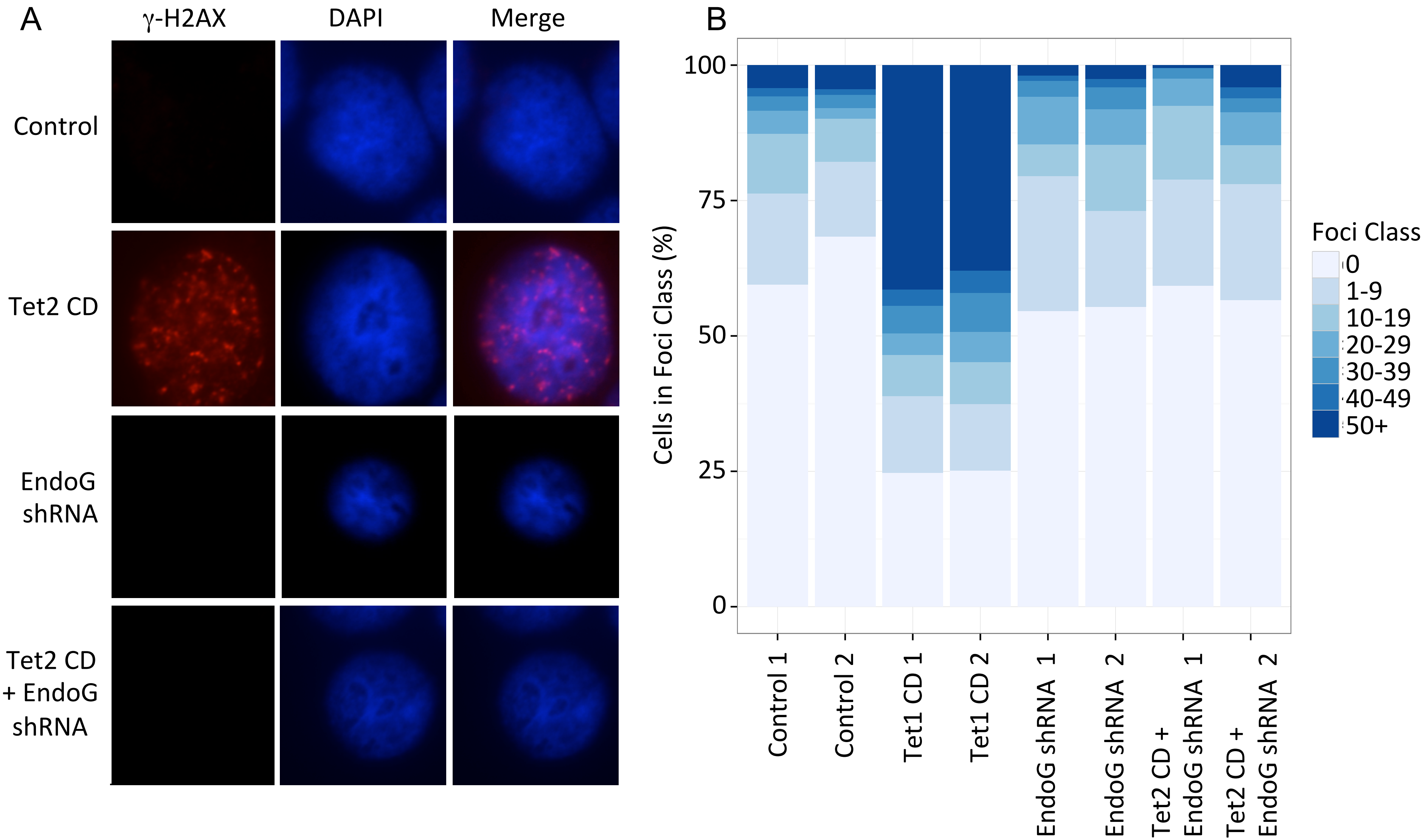

Supplemental Figure S17

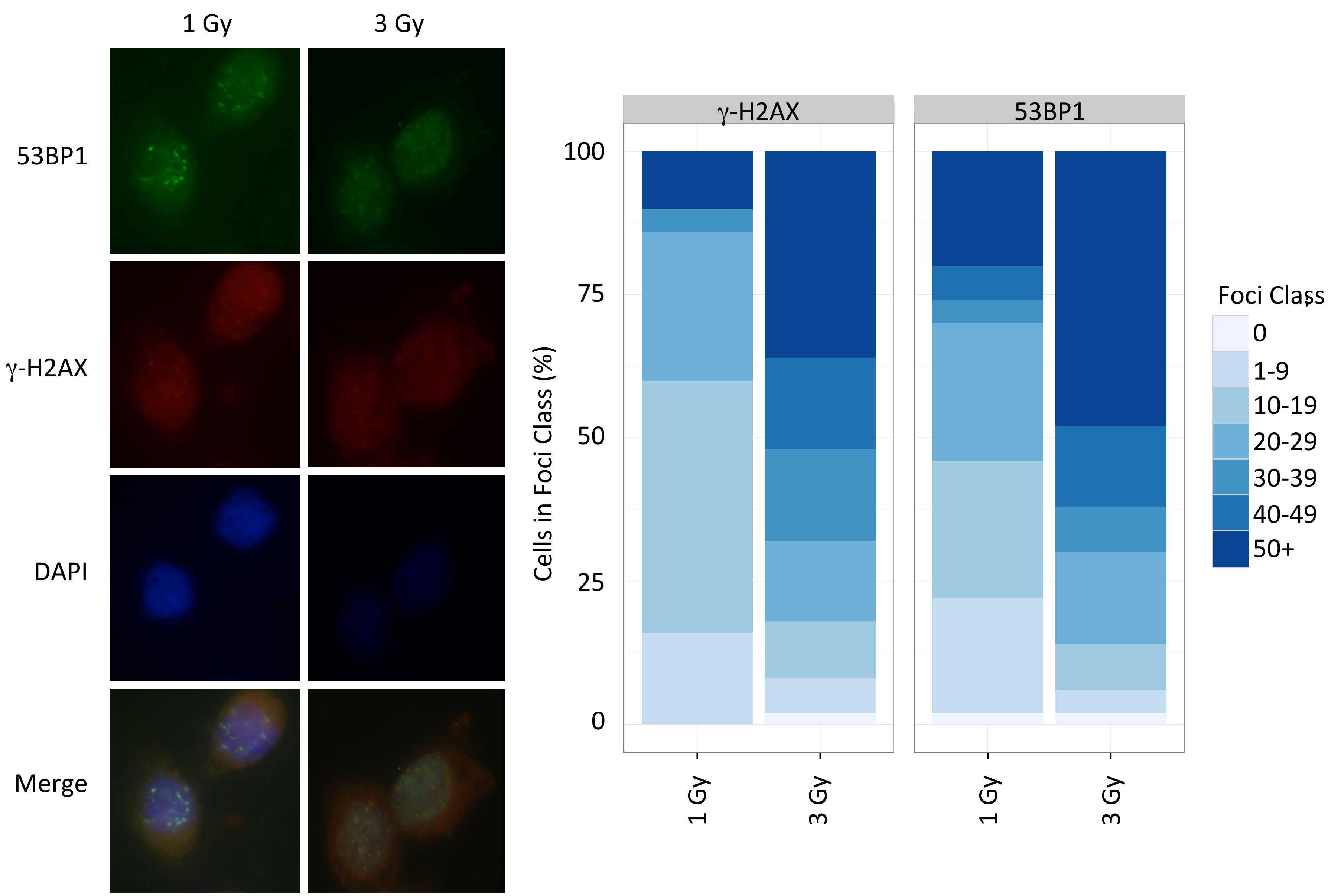

Supplemental Figure S18

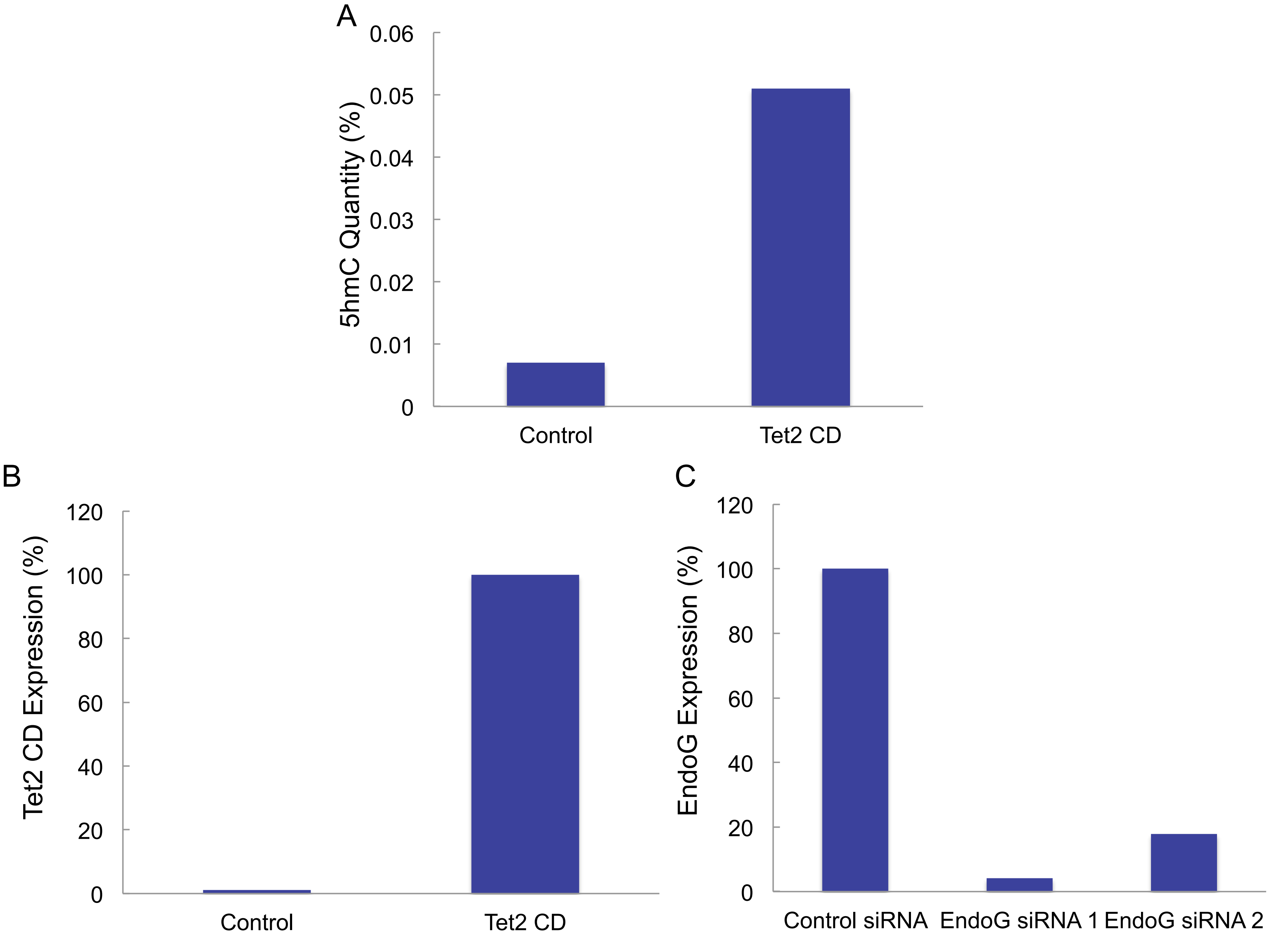

Supplemental Figure S19

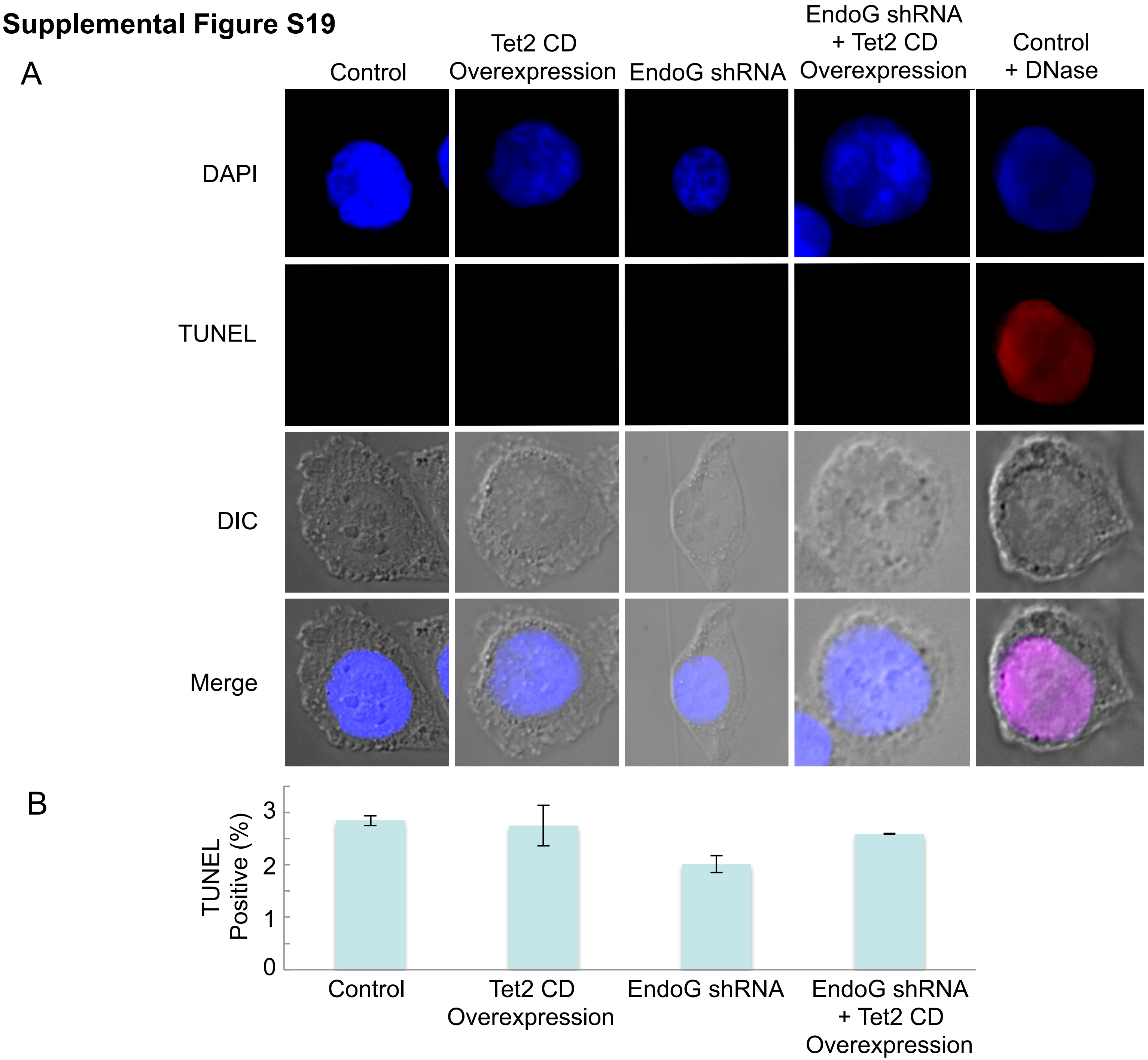

Supplemental Figure S20

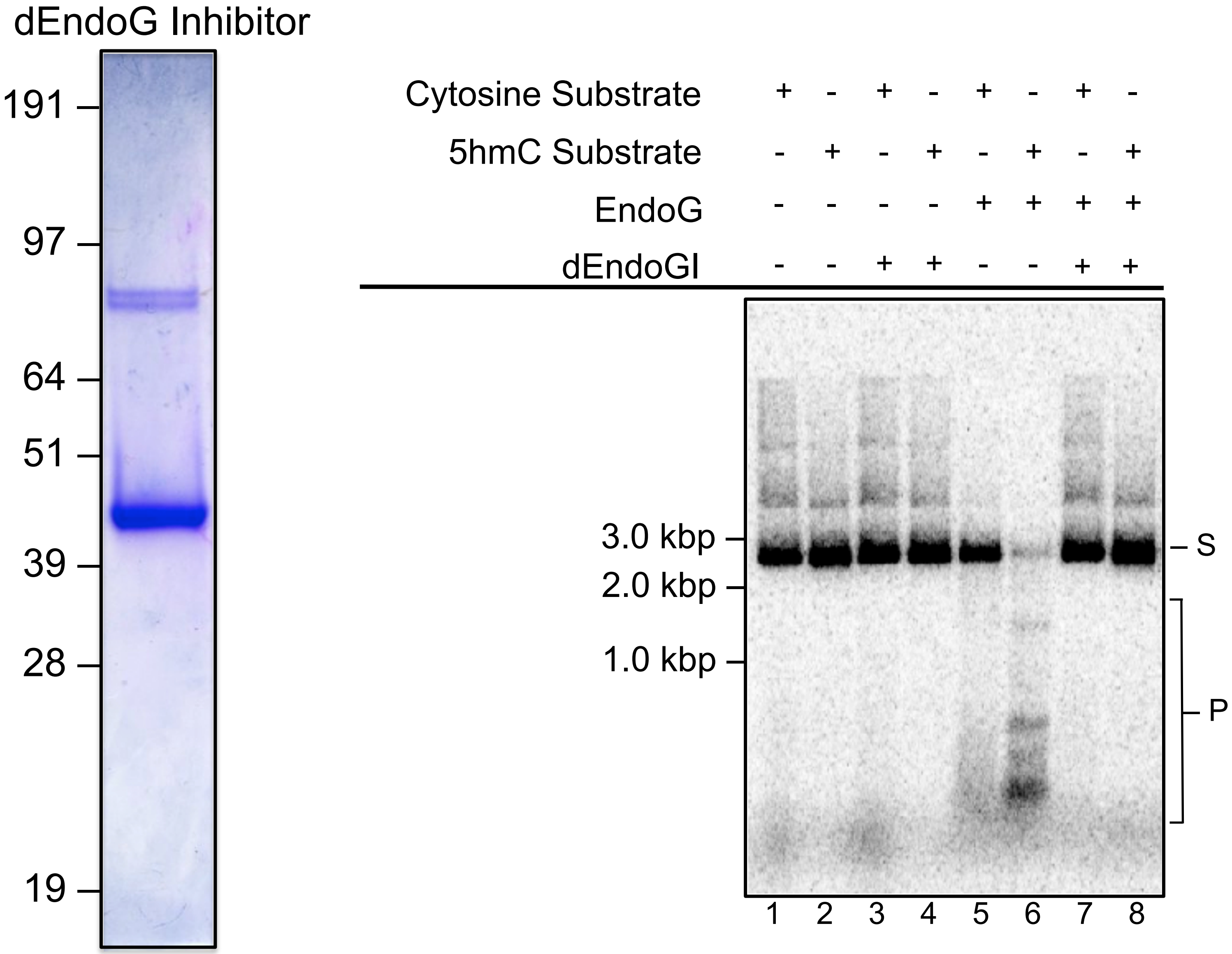

Supplemental Figure S21

```
Recombinaiton           CGGTGAAAACCTCTGACACATGCAGCTCCCGGAGACGGTCACAGCTTGTCTGTAAGCGGATGC  63
Substrate A             CGGTGAAAACCTCTGACACATGCAGCTCCCGGAGACGGTCACAGCTTGTCTGTAAGCGGATGC  63
Substrate B             ---------------------------------------------------------------  63
LiNE + Cyt              CGGTGAAAACCTCTGACACATGCAGCTCCCGGAGACGGTCACAGCTTGTCTGTAAGCGGATGC  63
LiNE + 5hmC             CGGTGAAAACCTCTGACACATGCAGCTCCCGGAGACGGTCACAGCTTGTCTGTAAGCGGATGC  63
LiNE + EndoGI + Cyt     CGGTGAAAACCTCTGACACATGCAGCTCCCGGAGACGGTCACAGCTTGTCTGTAAGCGGATGC  63
LiNE + EndoGI + 5hmC    CGGTGAAAACCTCTGACACATGCAGCTCCCGGAGACGGTCACAGCTTGTCTGTAAGCGGATGC  63

CGGGAGCAGACAAGCCCGTCAGGGCGCGTCAGCGGGTGTTGGCGGGTGTCGGGGCTGGCTTAACTATGCGGCATCAGAGCAGATTGTACT 153
CGGGAGCAGACAAGCCCGTCAGGGCGCGTCAGCGGGTGTTGGCGGGTGTCGGGGCTGGCTTAACTATGCGGCATCAGAGCAGATTGTACT 153
------------------------------------------------------------------------------------------ 153
CGGGAGCAGACAAGCCCGTCAGGGCGCGTCAGCGGGTGTTGGCGGGTGTCGGGGCTGGCTTAACTATGCGGCATCAGAGCAGATTGTACT 153
CGGGAGCAGACAAGCCCGTCAGGGCGCGTCAGCGGGTGTTGGCGGGTGTCGGGGCTGGCTTAACTATGCGGCATCAGAGCAGATTGTACT 153
CGGGAGCAGACAAGCCCGTCAGGGCGCGTCAGCGGGTGTTGGCGGGTGTCGGGGCTGGCTTAACTATGCGGCATCAGAGCAGATTGTACT 153
CGGGAGCAGACAAGCCCGTCAGGGCGCGTCAGCGGGTGTTGGCGGGTGTCGGGGCTGGCTTAACTATGCGGCATCAGAGCAGATTGTACT 153

GAGAGTGCACCATATGCGGTGTGAAATACCGCACAGATGCGTAAGGAGAAAATACCGCATCAGGCGCCATTCGCCATTCAGGCTGCGCAA 243
GAGAGTGCACCATATGCGGTGTGAAATACCGCACAGATGCGTAAGGAGAAAATACCGCATCAGGCGCCATTCGCCATTCAGGCTGCGCAA 243
-----------------------------------------------------------------GCCATTCGCCATTCAGGCTGCGCAA 243
GAGAGTGCACCATATGCGGTGTGAAATACCGCACAGATGCGTAAGGAGAAAATACCGCATCAGGCGCCATTCGCCATTCAGGCTGCGCAA 243
GAGAGTGCACCATATGCGGTGTGAAATACCGCACAGATGCGTAAGGAGAAAATACCGCATCAGGCGCCATTCGCCATTCAGGCTGCGCAA 243
GAGAGTGCACCATATGCGGTGTGAAATACCGCACAGATGCGTAAGGAGAAAATACCGCATCAGGCGCCATTCGCCATTCAGGCTGCGCAA 243
GAGAGTGCACCATATGCGGTGTGAAATACCGCACAGATGCGTAAGGAGAAAATACCGCATCAGGCGCCATTCGCCATTCAGGCTGCGCAA 243

CTGTTGGGAAGGGCGATCGGTGCGGGCCTCTTCGCTATTACGCCAGCTGGCGAAAGGGGGATGTGCTGCAAGGCGATTAAGTTGGGTAAC 333
CTGTTGGGAAGGGCGATCGGTGCGGGCCTCTTCGCTATTACGCCAGCTGGCGAAAGGGGGATGTGCTGCAAGGCGATTAAGTTGGGTAAC 333
CTGTTGGGAAGGGCGATCGGTGCGGGCCTCTTCGCTATTACGCCAGCTGGCGAAAGGGGGATGTGCTGCAAGGCGATTAAGTTGGGTAAC 333
CTGTTGGGAAGGGCGATCGGTGCGGGCCTCTTCGCTATTACGCCAGCTGGCGAAAGGGGGATGTGCTGCAAGGCGATTAAGTTGGGTAAC 333
CTGTTGGGAAGGGCGATCGGTGCGGGCCTCTTCGCTATTACGCCAGCTGGCGAAAGGGGGATGTGCTGCAAGGCGATTAAGTTGGGTAAC 333
CTGTTGGGAAGGGCGATCGGTGCGGGCCTCTTCGCTATTACGCCAGCTGGCGAAAGGGGGATGTGCTGCAAGGCGATTAAGTTGGGTAAC 333
CTGTTGGGAAGGGCGATCGGTGCGGGCCTCTTCGCTATTACGCCAGCTGGCGAAAGGGGGATGTGCTGCAAGGCGATTAAGTTGGGTAAC 333

GCCAGGGTTTTCCCAGTCACGACGTTGTAAAACGACGGCCAGTGAATTCGAGCTCGGTACCCGGGGATCCTCTAGAGTCGACCTGCA    420
GCCAGGGTTTTCCCAGTCACGACGTTGTAAAACGACGGCCAGTGAATTCGAGCTCGGTACCC-------------------------    420
GCCAGGGTTTTCCCAGTCACGACGTTGTAAAACGACGGCCAGTGAATTCGAGCTCGGTACCCGGGGATCCTCTAGAGTCGACCTGCA    420
GCCAGGGTTTTCCCAGTCACGACGTTGTAAAACGACGGCCAGTGAATTCGAGCTCGGTACCCGGGGATCCTCTAGAGTCGACCTGCA    420
GCCAGGGTTTTCCCAGTCACGACGTTGTAAAACGACGGCCAGTGAATTCGAGCTCGGTACCCGGGGATCCTCTAGAGTCGACCTGCA    420
GCCAGGGTTTTCCCAGTCACGACGTTGTAAAACGACGGCCAGTGAATTCGAGCTCGGTACCCGGGGATCCTCTAGAGTCGACCTGCA    420
GCCAGGGTTTTCCCAGTCACGACGTTGTAAAACGACGGCCAGTGAATTCGAGCTCGGTACCCGGGGATCCTCTAGAGTCGACCTGCA    420
```

Supplemental Table S1

2.7 kbp Substrate sequence

GGTACCCGGGGATCCTCTAGAGTCGACCTGCAGGCATGCAAGCTTGGCGT
AATCATGGTCATAGCTGTTTCCTGTGTGAAATTGTTATCCGCTCACAATTC
CACACAACATACGAGCCGGAAGCATAAAGTGTAAAGCCTGGGGTGCCTA
ATGAGTGAGCTAACTCACATTAATTGCGTTGCGCTCACTGCCCGCTTTCCA
GTCGGGAAACCTGTCGTGCCAGCTGCATTAATGAATCGGCCAACGCGCGG
GGAGAGGCGGTTTGCGTATTGGGCGCTCTTCCGCTTCCTCGCTCACTGACT
CGCTGCGCTCGGTCGTTCGGCTGCGGCGAGCGGTATCAGCTCACTCAAAG
GCGGTAATACGGTTATCCACAGAATCAGGGGATAACGCAGGAAAGAACA
TGTGAGCAAAAGGCCAGCAAAAGGCCAGGAACCGTAAAAAGGCCGCGTT
GCTGGCGTTTTTCCATAGGCTCCGCCCCCCTGACGAGCATCACAAAAATC
GACGCTCAAGTCAGAGGTGGCGAAACCCGACAGGACTATAAAGATACCA
GGGTTTCCCCCTGGAAGCTCCCTCGTGCGCTCTCCTGTTCCGACCCTGCCG
CTTACCGGATACCTGTCCGCCTTTCTCCCTTCGGGAAGCGTGGCGCTTTCT
CATAGCTCACGCTGTAGGTATCTCAGTTCGGTGTAGGTCGTTCGCTCCAAG
CTGGGCTGTGTGCACGAACCCCCCGTTCAGCCCGACCGCTGCGCCTTATCC
GGTAACTATCGTCTTGAGTCCAACCCGGTAAGACACGACTTATCGCCACT
GGCAGCAGCCACTGGTAACAGGATTAGCAGAGCGAGGTATGTAGGCGGT
GCTACAGAGTTCTTGAAGTGGTGGCCTAACTACGGCTACACTAGAAGAAC
AGTATTTGGTATCTGCGCTCTGCTGAAGCCAGTTACCTTCGGAAAAAGAG
TTGGTAGCTCTTGATCCGGCAAACAAACCACCGCTGGTAGCGGTGGTTTTT
TTGTTTGCAAGCAGCAGATTACGCGCAGAAAAAAAGGATCTCAAGAAGAT
CCTTTGATCTTTTCTACGGGGTCTGACGCTCAGTGGAACGAAAACTCACGT
TAAGGGATTTTGGTCATGAGATTATCAAAAAGGATCTTCACCTAGATCCTT
TTAAATTAAAAATGAAGTTTTAAATCAATCTAAAGTATATATGAGTAAAC
TTGGTCTGACAGTTACCAATGCTTAATCAGTGAGGCACCTATCTCAGCGAT
CTGTCTATTTCGTTCATCCATAGTTGCCTGACTCCCCGTCGTGTAGATAAC
TACGATACGGGAGGGCTTACCATCTGGCCCCAGTGCTGCAATGATACCGC
GAGACCCACGCTCACCGGCTCCAGATTTATCAGCAATAAACCAGCCAGCC
GGAAGGGCCGAGCGCAGAAGTGGTCCTGCAACTTTATCCGCCTCCATCCA
GTCTATTAATTGTTGCCGGGAAGCTAGAGTAAGTAGTTCGCCAGTTAATA
GTTTGCGCAACGTTGTTGCCATTGCTACAGGCATCGTGGTGTCACGCTCGT
CGTTTGGTATGGCTTCATTCAGCTCCGGTTCCCAACGATCAAGGCGAGTTA
CATGATCCCCCATGTTGTGCAAAAAAGCGGTTAGCTCCTTCGGTCCTCCGA
TCGTTGTCAGAAGTAAGTTGGCCGCAGTGTTATCACTCATGGTTATGGCAG
CACTGCATAATTCTCTTACTGTCATGCCATCCGTAAGATGCTTTTCTGTGA
CTGGTGAGTACTCAACCAAGTCATTCTGAGAATAGTGTATGCGGCGACCG
AGTTGCTCTTGCCCGGCGTCAATACGGGATAATACCGCGCCACATAGCAG
AACTTTAAAAGTGCTCATCATTGGAAAACGTTCTTCGGGGCGAAAACTCT
CAAGGATCTTACCGCTGTTGAGATCCAGTTCGATGTAACCCACTCGTGCAC
CCAACTGATCTTCAGCATCTTTTACTTTCACCAGCGTTTCTGGGTGAGCAA
AAACAGGAAGGCAAAATGCCGCAAAAAAGGGAATAAGGGCGACACGGA
AATGTTGAATACTCATACTCTTCCTTTTTCAATATTATTGAAGCATTTATCA
GGGTTATTGTCTCATGAGCGGATACATATTTGAATGTATTTAGAAAAATA
AACAAATAGGGGTTCCGCGCACATTTCCCCGAAAAGTGCCACCTGACGTC
TAAGAAACCATTATTATCATGACATTAACCTATAAAAATAGGCGTATCAC
GAGGCCCTTTCGTCTCGCGCGTTTCGGTGATGACGGTGAAAACCTCTGAC

ACATGCAGCTCCCGGAGACGGTCACAGCTTGTCTGTAAGCGGATGCCGGG
AGCAGACAAGCCCGTCAGGGCGCGTCAGCGGGTGTTGGCGGGTGTCGGG
GCTGGCTTAACTATGCGGCATCAGAGCAGATTGTACTGAGAGTGCACCAT
ATGCGGTGTGAAATACCGCACAGATGCGTAAGGAGAAAATACCGCATCA
GGCGCCATTCGCCATTCAGGCTGCGCAACTGTTGGGAAGGGCGATCGGTG
CGGGCCTCTTCGCTATTACGCCAGCTGGCGAAAGGGGGATGTGCTGCAAG
GCGATTAAGTTGGGTAACGCCAGGGTTTTCCCAGTCACGACGTTGTAAAA
CGACGGCCAGTGAATTCGAGCTCGGTACCCGGGGATCCTCTAGAGT

pUC19 For and pUC19 Rev primers (Sequences in Supplemental Table S2) were used to amplify the 2.7 kbp substrate

CUT ASSAY SUBSTRATE DESIGN

Core Substrate Insert Sequence

Top pUC-WT

5′-CCCGTCGTGTAGATAACTACGATACGGGAGGGCTTACCATCTGGCCCCA
GTGCTGCAATGATACCGCGAGACCCACGCTCACCA-3′

Bottom pUC-WT

5′-GGTGAGCGTGGGTCTCGCGGTATCATTGCAGCACTGGGGCCAGATGGT
AAGCCCTCCCGTATCGTAGTTATCTACACGACGGGA-3′

Mutated Core Insert Sequence

Top pUC-Mut

5′-CCCGTCGTGTAGATAACTACGATACGGGAGGGCTTACCATCTAATTTTA
GTGCTGCAATGATACCGCGAGACCCACGCTCACCA-3′

Bot pUC-Mut

5′-GGTGAGCGTGGGTCTCGCGGTATCATTGCAGCACTAAAATTAGATGGTA
AGCCCTCCCGTATCGTAGTTATCTACACGACGGGA-3′

Each Sequence was annealed to its complement and cloned into pCR2.1-Topo.
The sequence was amplified from the vector using pCR2.1 For and pCR2.1 Rev creating the 130 bp substrate shown utilized in Figure 3A. These inserts after ligation into pCR2.1-Topo created the Core Substrate and the Core Mutated Substrate used in Figure 4.

RECOMBINATION ASSAY SUBSTRATE DESIGN

Substrate A sequence

TCGCGCGTTTCGGTGATGACGGTGAAAACCTCTGACACATGCAGCTCCCG
GAGACGGTCACAGCTTGTCTGTAAGCGGATGCCGGGAGCAGACAAGCCC

GTCAGGGCGCGTCAGCGGGTGTTGGCGGGTGTCGGGGCTGGCTTAACTAT
GCGGCATCAGAGCAGATTGTACTGAGAGTGCACCATATGCGGTGTGAAAT
ACCGCACAGATGCGTAAGGAGAAAATACCGCATCAGGCGCCATTCGCCAT
TCAGGCTGCGCAACTGTTGGGAAGGGCGATCGGTGCGGGCCTCTTCGCTT
TACGCCAGCTGGCGAAAGGGGGATGTGCTGCAAGGCGATTAAGTTGGGTA
ACGCCAGGGTTTTCCCAGTCACGACGTTGTAAAACGACGGCCAGTGAATT
CGAGCTCGGTACCCGACCTGCAGGCATGCAAGCTTGGCGTAATCATGGCA
TAGCTGTTTCCTGTGTGAAATTGTTATCCGCTCACAATTCCACACAACATA
CGAGCCGGAAGCATAAAGTGTAAAGCCTGGGGTGCCTAATGAGTGAGCT
AACTCACATTAATTGCGTTGCGCTCACTGCCCGCTTTCCAGTCGGGAAACC
TGTCGTGCCAGCTGCATTAATGAATCGGCCAACGCGCGGGGAGAGGCGGT
TTGCGTATTGGGCGCTCTTCCGCTTCCTCGCTCACTGACTCGCTGCGCTCG
GTCGTTCGGCTGCGGCGAGCGGTATCAGCTCACTCAAAGGCGGTAATACG
GTTATCCACAGAATCAGGGGATAACGCAGGAAAGAACATGTGAGCAAAA
GGCCAGCAAAAGGCCAGGAACCGTAAAAAGGCCGCGTTGCTGGCGTTTTT
CCATAGGCTCCGCCCCCCTGACGAGCATCACAAAAATCGACGCTCAAGTC
AGAGGTGGCGAAACCCGACAGGACTATAAAGATACCAGGCGTTTCCCCCT
GGAAGCTCCCTCGTGCGCTCTCCTGTTCCGACCCTGCCGCTTACCGGATAC
CTGTCCGCCTTTCTCCCTTCGGGAAGCGTGGCGCTTTCTCATAGCTCACGC
TGTAGGTATCTCAGTTCGGTGTAGGTCGTTCGCTCCAAGCTGGGCTGTGTG
CACGAACCCCCCGTTCAGCCCGACCGCTGCGCCTTATCCGGTAACTATCGT
CTTGAGTCCAACCCGGTAAGACACGACTTATCGCCACTGGCAGCAGCCAC
TGGTAACAGGATTAGCAGAGCGAGGTATGTAGGCGGTGCTACAGAGTTCT
TGAAGTGGTGGCCTAACTACGGCTACACTAGAAGAACAGTATTTGGTATC
TGCGCTCTGCTGAAGCCAGTTACCTTCGGAAAAAGAGTTGGTAGCTCTTG
ATCCGGCAAACAAACCACCGCTGGTAGCGGTGGTTTTTTTGTTTGCAAGC
AGCAGATTACGCGCAGAAAAAAAGGATCTCAAGAAGATCCTTTGATCTTT
TCTACGGGGTCTGACGCTCAGTGGAACGAAAACTCACGTTAAGGGATTTT
GGTCATGAGATTATCAAAAAGGATCTTCACCTAGATCCTTTTAAATTAAA
AATGAAGTTTTAAATCAATCTAAAGTATATATGAGTAAACTTGGTCTGAC
AGTTACCAATGCTTAATCAGTGAGGCACCTATCTCAGCGATCTGTCTATTT
CGTTCATCCATAGTTGCCTGACTCCCCGTCGTGTAGATAACTACGATACGG
GAGGGCTTACCATCTGGCCCCAGTGCTGCAATGATACCGCGAGACCCACG
CTCACCGGCTCCAGATTTATCAGCAATAAACCAGCCAGCCGGAAGGGCCG
AGCGCAGAAGTGGTCCTGCAACTTTATCCGCCTCCATCCAGTCTATTAATT
GTTGCCGGGAAGCTAGAGTAAGTAGTTCGCCAGTTAATAGTTTGCGCAAC
GTTGTTGCCATTGCTACAGGCATCGTGGTGTCACGCTCGTCGTTTGGTATG
GCTTCATTCAGCTCCGGTTCCCAACGATCAAGGCGAGTTACATGATCCCCC
ATGTTGTGCAAAAAAGCGGTTAGCTCCTTCGGTCCTCCGATCGTTGTCAGA
AGTAAGTTGGCCGCAGTGTTATCACTCATGGTTATGGCAGCACTGCATAA
TTCTCTTACTGTCATGCCATCCGTAAGATGCTTTTCTGTGACTGGTGAGTA
CTCAACCAAGTCATTCTGAGAATAGTGTATGCGGCGACCGAGTTGCTCTT
GCCCGGCGTCAATACGGGATAATACCGCGCCACATAGCAGAACTTTAAAA
GTGCTCATCATTGGAAAACGTTCTTCGGGGCGAAAACTCTCAAGGATCTT
ACCGCTGTTGAGATCCAGTTCGATGTAACCCACTCGTGCACCCAACTGATC
TTCAGCATCTTTTACTTTCACCAGCGTTTCTGGGTGAGCAAAAACAGGAAG
GCAAAATGCCGCAAAAAAGGGAATAAGGGCGACACGGAAATGTTGAATA
CTCATACTCTTCCTTTTTCAATATTATTGAAGCATTTATCAGGGTTATTGTC
TCATGAGCGGATACATATTTGAATGTATTTAGAAAAATAAACAAATAGGG
GTTCCGCGCACATTTCCCCGAAAAGTGCCACCTGACGTCTAAGAAACCAT

TATTATCATGACATTAACCTATAAAAATAGGCGTATCACGAGGCCCTTTCG
TC

Substrate B sequence

GCCATTCGCCATTCAGGCTGCGCAACTGTTGGGAAGGGCGATCGGTGCGG
GCCTCTTCGCTATTACGCCAGCTGGCGAAAGGGGGATGTGCTGCAAGGCG
ATTAAGTTGGGTAACGCCAGGGTTTTCCCAGTCACGACGTTGTAAAACGA
CGGCCAGTGAATTCGAGCTCGGTACCCGGGGATCCTCTAGAGTCGACCTG
CAGGCATGCAAGCTTGGCGTAATCATGGTCATAGCTGTTTCCTGTGTGAA
ATTGTTATCCGCTCACAATTCCACACAACATACGAGCCGGAAGCATAAAG
TGTAAAGCCTGGGGTGCCTAATGAGTGAGCTAACTCACATTAATTGCGTT
GCGCTCACTGCCCGCTTTCCAGTCGGGAAACCTGTCGTGCCAGCTGCATTA
ATGAATCGGCCAACGCGCGGGGAGAGGCGGTTTGCGTATTGGGCGCTCTT
CCGCTTCCTCGCTCACTGACTCGCTGCGCTCGGTCGTTCGGCTGCGGCGAG
CGGTATCAGCTCACTCAAAGGCGGTAATACGGTTATCCACAGAATCAGGG
GATAACGCAGGAAAGAACATGTGAGCAAAAGGCCAGCAAAAGGCCAGGA
ACCGTAAAAAGGCCGCGTTGCTGGCGTTTTTCCATAGGCTCCGCCCCCCTG
ACGAGCATCACAAAAATCGACGCTCAAGTCAGAGGTGGCGAAACCCGAC
AGGACTATAAAGATACCAGGCGTTTCCCCCTGGAAGCTCCCTCGTGCGCT
CTCCTGTTCCGACCCTGCCGCTTACCGGATACCTGTCCGCCTTTCTCCCTTC
GGGAAGCGTGGCGCTTTCTCATAGCTCACGCTGTAGGTATCTCAGTTCGGT
GTAGGTCGTTCGCTCCAAGCTGGGCTGTGTGCACGAACCCCCCGTTCAGC
CCGACCGCTGCGCCTTATCCGGTAACTATCGTCTTGAGTCCAACCCGGTAA
GACACGACTTATCGCCACTGGCAGCAGCCACTGGTAACAGGATTAGCAGA
GCGAGGTATGTAGGCGGTGCTACAGAGTTCTTGAAGTGGTGGCCTAACTA
CGGCTACACTAGAAGAACAGTATTTGGTATCTGCGCTCTGCTGAAGCCAG
TTACCTTCGGAAAAAGAGTTGGTAGCTCTTGATCCGGCAAACAAACCACC
GCTGGTAGCGGTGGTTTTTTTGTTTGCAAGCAGCAGATTACGCGCAGAAA
AAAAGGATCTCAAGAAGATCCTTTGATCTTTTCTACGGGGTCTGACGCTCA
GTGGAACGAAAACTCACGTTAAGGGATTTTGGTCATGAGATTATCAAAAA
GGATCTTCACCTAGATCCTTTTAAATTAAAAATGAAGTTTTAAATCAATCT
AAAGTATATATGAGTAAACTTGGTCTGACAGTTACCAATGCTTAATCAGT
GAGGCACCTATCTCAGCGATCTGTCTATTTCGTTCATCCATAGTTGCCTGA
CTCCCCGTCGTGTAGATAACTACGATACGGGAGGGCTTACCATCTGGCCC
CAGTGCTGCAATGATACCGCGAGACCCACGCTCACCGGCTCCAGATTTAT
CAGCAATAAACCAGCCAGCCGGAAGGGCCGAGCGCAGAAGTGGTCCTGC
AACTTTATCCGCCTCCATCCAGTCTATTAATTGTTGCCGGGAAGCTAGAGT
AAGTAGTTCGCCAGTTAATAGTTTGCGCAACGTTGTTGCCATTGCTACAGG
CATCGTGGTGTCACGCTCGTCGTTTGGTATGGCTTCATTCAGCTCCGGTTC
CCAACGATCAAGGCGAGTTACATGATCCCCCATGTTGTGCAAAAAAGCGG
TTAGCTCCTTCGGTCCTCCGATCGTTGTCAGAAGTAAGTTGGCCGCAGTGT
TATCACTCATGGTTATGGCAGCACTGCATAATTCTCTTACTGTCATGCCAT
CCGTAAGATGCTTTTCTGTGACTGGTGAGTACTCAACCAAGTCATTCTGAG
AATAGTGTATGCGGCGACCGAGTTGCTCTTGCCCGGCGTCAATACGGGAT
AATACCGCGCCACATAGCAGAACTTTAAAAGTGCTCATCATTGGAAAACG
TTCTTCGGGGCGAAAACTCTCAAGGATCTTACCGCTGTTGAGATCCAGTTC
GATGTAACCCACTCGTGCACCCAACTGATCTTCAGCATCTTTTACTTTCAC
CAGCGTTTCTGGGTGAGCAAAAACAGGAAGGCAAAATGCCGCAAAAAAG
GGAATAAGGGCGACACGGAAATGTTGAATACTCATACTCTTCCTTTTTCA

ATATTATTGAAGCATTTATCAGGGTTATTGTCTCATGAGCGGATACATATT
TGAATGTATTTAGAAAAATAAACAAATAGGGGTTCCGCGCACATTTCCCC
GAAAAGTGCCACCTGAC

Substrates were amplified using the Recombination A primer set in Supplemental Table S2

EXPRESSION CONSTRUCTS

pET28a-EndoG

TGGCGAATGGGACGCGCCCTGTAGCGGCGCATTAAGCGCGGCGGGTGTGG
TGGTTACGCGCAGCGTGACCGCTACACTTGCCAGCGCCCTAGCGCCCGCT
CCTTTCGCTTTCTTCCCTTCCTTTCTCGCCACGTTCGCCGGCTTTCCCCGTC
AAGCTCTAAATCGGGGGCTCCCTTTAGGGTTCCGATTTAGTGCTTTACGGC
ACCTCGACCCCAAAAAACTTGATTAGGGTGATGGTTCACGTAGTGGGCCA
TCGCCCTGATAGACGGTTTTTCGCCCTTTGACGTTGGAGTCCACGTTCTTT
AATAGTGGACTCTTGTTCCAAACTGGAACAACACTCAACCCTATCTCGGTC
TATTCTTTTGATTTATAAGGGATTTTGCCGATTTCGGCCTATTGGTTAAAA
AATGAGCTGATTTAACAAAAATTTAACGCGAATTTTAACAAAATATTAAC
GTTTACAATTTCAGGTGGCACTTTTCGGGGAAATGTGCGCGGAACCCCTAT
TTGTTTATTTTTCTAAATACATTCAAATATGTATCCGCTCATGAATTAATTC
TTAGAAAAACTCATCGAGCATCAAATGAAACTGCAATTTATTCATATCAG
GATTATCAATACCATATTTTTGAAAAAGCCGTTTCTGTAATGAAGGAGAA
AACTCACCGAGGCAGTTCCATAGGATGGCAAGATCCTGGTATCGGTCTGC
GATTCCGACTCGTCCAACATCAATACAACCTATTAATTTCCCCTCGTCAAA
AATAAGGTTATCAAGTGAGAAATCACCATGAGTGACGACTGAATCCGGTG
AGAATGGCAAAAGTTTATGCATTTCTTTCCAGACTTGTTCAACAGGCCAGC
CATTACGCTCGTCATCAAAATCACTCGCATCAACCAAACCGTTATTCATTC
GTGATTGCGCCTGAGCGAGACGAAATACGCGATCGCTGTTAAAAGGACAA
TTACAAACAGGAATCGAATGCAACCGGCGCAGGAACACTGCCAGCGCAT
CAACAATATTTTCACCTGAATCAGGATATTCTTCTAATACCTGGAATGCTG
TTTTCCCGGGGATCGCAGTGGTGAGTAACCATGCATCATCAGGAGTACGG
ATAAAATGCTTGATGGTCGGAAGAGGCATAAATTCCGTCAGCCAGTTTAG
TCTGACCATCTCATCTGTAACATCATTGGCAACGCTACCTTTGCCATGTTT
CAGAAACAACTCTGGCGCATCGGGCTTCCCATACAATCGATAGATTGTCG
CACCTGATTGCCCGACATTATCGCGAGCCCATTTATACCCATATAAATCAG
CATCCATGTTGGAATTTAATCGCGGCCTAGAGCAAGACGTTTCCCGTTGA
ATATGGCTCATAACACCCCTTGTATTACTGTTTATGTAAGCAGACAGTTTT
ATTGTTCATGACCAAAATCCCTTAACGTGAGTTTTCGTTCCACTGAGCGTC
AGACCCCGTAGAAAAGATCAAAGGATCTTCTTGAGATCCTTTTTTTCTGCG
CGTAATCTGCTGCTTGCAAACAAAAAAACCACCGCTACCAGCGGTGGTTT
GTTTGCCGGATCAAGAGCTACCAACTCTTTTTCCGAAGGTAACTGGCTTCA
GCAGAGCGCAGATACCAAATACTGTCCTTCTAGTGTAGCCGTAGTTAGGC
CACCACTTCAAGAACTCTGTAGCACCGCCTACATACCTCGCTCTGCTAATC
CTGTTACCAGTGGCTGCTGCCAGTGGCGATAAGTCGTGTCTTACCGGGTTG
GACTCAAGACGATAGTTACCGGATAAGGCGCAGCGGTCGGGCTGAACGG
GGGGTTCGTGCACACAGCCCAGCTTGGAGCGAACGACCTACACCGAACTG
AGATACCTACAGCGTGAGCTATGAGAAAGCGCCACGCTTCCCGAAGGGA
GAAAGGCGGACAGGTATCCGGTAAGCGGCAGGGTCGGAACAGGAGAGCG

CACGAGGGAGCTTCCAGGGGGAAACGCCTGGTATCTTTATAGTCCTGTCG
GGTTTCGCCACCTCTGACTTGAGCGTCGATTTTTGTGATGCTCGTCAGGGG
GGCGGAGCCTATGGAAAAACGCCAGCAACGCGGCCTTTTTACGGTTCCTG
GCCTTTTGCTGGCCTTTTGCTCACATGTTCTTTCCTGCGTTATCCCCTGATT
CTGTGGATAACCGTATTACCGCCTTTGAGTGAGCTGATACCGCTCGCCGCA
GCCGAACGACCGAGCGCAGCGAGTCAGTGAGCGAGGAAGCGGAAGAGCG
CCTGATGCGGTATTTTCTCCTTACGCATCTGTGCGGTATTTCACACCGCAT
ATATGGTGCACTCTCAGTACAATCTGCTCTGATGCCGCATAGTTAAGCCAG
TATACACTCCGCTATCGCTACGTGACTGGGTCATGGCTGCGCCCCGACACC
CGCCAACACCCGCTGACGCGCCCTGACGGGCTTGTCTGCTCCCGGCATCC
GCTTACAGACAAGCTGTGACCGTCTCCGGGAGCTGCATGTGTCAGAGGTT
TTCACCGTCATCACCGAAACGCGCGAGGCAGCTGCGGTAAAGCTCATCAG
CGTGGTCGTGAAGCGATTCACAGATGTCTGCCTGTTCATCCGCGTCCAGCT
CGTTGAGTTTCTCCAGAAGCGTTAATGTCTGGCTTCTGATAAAGCGGGCCA
TGTTAAGGGCGGTTTTTTCCTGTTTGGTCACTGATGCCTCCGTGTAAGGGG
GATTTCTGTTCATGGGGGTAATGATACCGATGAAACGAGAGAGGATGCTC
ACGATACGGGTTACTGATGATGAACATGCCCGGTTACTGGAACGTTGTGA
GGGTAAACAACTGGCGGTATGGATGCGGCGGGACCAGAGAAAAATCACT
CAGGGTCAATGCCAGCGCTTCGTTAATACAGATGTAGGTGTTCCACAGGG
TAGCCAGCAGCATCCTGCGATGCAGATCCGGAACATAATGGTGCAGGGCG
CTGACTTCCGCGTTTCCAGACTTTACGAAACACGGAAACCGAAGACCATT
CATGTTGTTGCTCAGGTCGCAGACGTTTTGCAGCAGCAGTCGCTTCACGTT
CGCTCGCGTATCGGTGATTCATTCTGCTAACCAGTAAGGCAACCCCGCCA
GCCTAGCCGGGTCCTCAACGACAGGAGCACGATCATGCGCACCCGTGGGG
CCGCCATGCCGGCGATAATGGCCTGCTTCTCGCCGAAACGTTTGGTGGCG
GGACCAGTGACGAAGGCTTGAGCGAGGGCGTGCAAGATTCCGAATACCG
CAAGCGACAGGCCGATCATCGTCGCGCTCCAGCGAAAGCGGTCCTCGCCG
AAAATGACCCAGAGCGCTGCCGGCACCTGTCCTACGAGTTGCATGATAAA
GAAGACAGTCATAAGTGCGGCGACGATAGTCATGCCCCGCGCCCACCGGA
AGGAGCTGACTGGGTTGAAGGCTCTCAAGGGCATCGGTCGAGATCCCGGT
GCCTAATGAGTGAGCTAACTTACATTAATTGCGTTGCGCTCACTGCCCGCT
TTCCAGTCGGGAAACCTGTCGTGCCAGCTGCATTAATGAATCGGCCAACG
CGCGGGGAGAGGCGGTTTGCGTATTGGGCGCCAGGGTGGTTTTTCTTTTCA
CCAGTGAGACGGGCAACAGCTGATTGCCCTTCACCGCCTGGCCCTGAGAG
AGTTGCAGCAAGCGGTCCACGCTGGTTTGCCCCAGCAGGCGAAAATCCTG
TTTGATGGTGGTTAACGGCGGGATATAACATGAGCTGTCTTCGGTATCGTC
GTATCCCACTACCGAGATATCCGCACCAACGCGCAGCCCGGACTCGGTAA
TGGCGCGCATTGCGCCCAGCGCCATCTGATCGTTGGCAACCAGCATCGCA
GTGGGAACGATGCCCTCATTCAGCATTTGCATGGTTTGTTGAAAACCGGA
CATGGCACTCCAGTCGCCTTCCCGTTCCGCTATCGGCTGAATTTGATTGCG
AGTGAGATATTTATGCCAGCCAGCCAGACGCAGACGCGCCGAGACAGAA
CTTAATGGGCCCGCTAACAGCGCGATTTGCTGGTGACCCAATGCGACCAG
ATGCTCCACGCCCAGTCGCGTACCGTCTTCATGGGAGAAAATAATACTGT
TGATGGGTGTCTGGTCAGAGACATCAAGAAATAACGCCGGAACATTAGTG
CAGGCAGCTTCCACAGCAATGGCATCCTGGTCATCCAGCGGATAGTTAAT
GATCAGCCCACTGACGCGTTGCGCGAGAAGATTGTGCACCGCCGCTTTAC
AGGCTTCGACGCCGCTTCGTTCTACCATCGACACCACCACGCTGGCACCC
AGTTGATCGGCGCGAGATTTAATCGCCGCGACAATTTGCGACGGCGCGTG
CAGGGCCAGACTGGAGGTGGCAACGCCAATCAGCAACGACTGTTTGCCCG
CCAGTTGTTGTGCCACGCGGTTGGGAATGTAATTCAGCTCCGCCATCGCCG

CTTCCACTTTTTCCCGCGTTTTCGCAGAAACGTGGCTGGCCTGGTTCACCA
CGCGGGAAACGGTCTGATAAGAGACACCGGCATACTCTGCGACATCGTAT
AACGTTACTGGTTTCACATTCACCACCCTGAATTGACTCTCTTCCGGGCGC
TATCATGCCATACCGCGAAAGGTTTTGCGCCATTCGATGGTGTCCGGGATC
TCGACGCTCTCCCTTATGCGACTCCTGCATTAGGAAGCAGCCCAGTAGTA
GGTTGAGGCCGTTGAGCACCGCCGCCGCAAGGAATGGTGCATGCAAGGA
GATGGCGCCCAACAGTCCCCCGGCCACGGGGCCTGCCACCATACCCACGC
CGAAACAAGCGCTCATGAGCCCGAAGTGGCGAGCCCGATCTTCCCCATCG
GTGATGTCGGCGATATAGGCGCCAGCAACCGCACCTGTGGCGCCGGTGAT
GCCGGCCACGATGCGTCCGGCGTAGAGGATCGAGATCTCGATCCCGCGAA
ATTAATACGACTCACTATAGGGGAATTGTGAGCGGATAACAATTCCCCTC
TAGAAATAATTTTGTTTAACTTTAAGAAGGAGATATACCATGGGCAGCAG
CCATCATCATCATCATCACAGCAGCGGCCTGGTGCCGCGCGGCAGCCAT
ATGCGCGCGCTGCGGGCCGGCCTGACCCTAGCGCTGGGCGCGGGGCTGGG
CGCCGCGGCAGAGCATTGGCGGCGGCGGGAGGGCAAAGCGCCGGGGCTG
CTGGGCCGAGTGCCATTGTTGCCGGTGGTCGCGGCCGATCTTCCCGCGCTG
CCGGGGGGACCGGCGGGCGGCACCGGGGAACTGGCCAAGTACGGGCTGC
CCGGCGTGGCGCAGCTCCGGAGCCGCGAGTCCTACGTGCTTAGCTACGAC
CCGCGCACGCGCGGTGCGCTCTGGGTGTTGGAGCAGCTGAGGCCAGAGCG
GCTCCGTGGCGACGGGGACCGTAGCGCCTGCGACTTCCGCGAGGATGACT
CTGTGCACGCGTACCACCGCGCCACCAATGCGGACTACCGCGGCAGTGGC
TTTGACCGCGGCCATTTGGCCGCCGCCGCCAACCACCGCTGGAGTCAGCG
GGCCATGGACGACACCTTCTACCTGAGCAACGTAGCGCCTCAGGTGCCAC
ACCTCAACCAGAATGCCTGGAACAACCTTGAGAGGTACAGCCGCAGCTTG
ACGCGAACTTACCAAAATGTCTATGTCTGCACGGGGCCGCTTTTCCTGCCC
AGGACCGAGGCTGATGGGAAGTCCTATGTGAAGTACCAGGTTATTGGGAA
GAACCACGTGGCAGTGCCCACACACTTCTTCAAGGTGCTGATCCTGGAGG
CAGCCGGTGGGCAGATCGAGCTACGTTCCTACGTGATGCCCAATGCCCCC
GTGGATGAGACCATCCCTCTGGAGCGGTTCCTGGTGCCCATCGAGAGCAT
CGAGCGGGCCTCGGGATTGCTCTTCGTGCCCAATATTCTGGCTCGAGCTGG
AAACCTCAAGGCTATCACTGCTGGCAGCAAGTGAGCGGCCGCACTCGAGC
ACCACCACCACCACCACTGAGATCCGGCTGCTAACAAAGCCCGAAAGGA
AGCTGAGTTGGCTGCTGCCACCGCTGAGCAATAACTAGCATAACCCCTTG
GGGCCTCTAAACGGGTCTTGAGGGGTTTTTTGCTGAAAGGAGGAACTATA
TCCGGAT

pET28a-EndoG H128A

TGGCGAATGGGACGCGCCCTGTAGCGGCGCATTAAGCGCGGCGGGTGTGG
TGGTTACGCGCAGCGTGACCGCTACACTTGCCAGCGCCCTAGCGCCCGCT
CCTTTCGCTTTCTTCCCTTCCTTTCTCGCCACGTTCGCCGGCTTTCCCCGTC
AAGCTCTAAATCGGGGGCTCCCTTTAGGGTTCCGATTTAGTGCTTTACGGC
ACCTCGACCCCAAAAAACTTGATTAGGGTGATGGTTCACGTAGTGGGCCA
TCGCCCTGATAGACGGTTTTTCGCCCTTTGACGTTGGAGTCCACGTTCTTT
AATAGTGGACTCTTGTTCCAAACTGGAACAACACTCAACCCTATCTCGGTC
TATTCTTTTGATTTATAAGGGATTTTGCCGATTTCGGCCTATTGGTTAAAA
AATGAGCTGATTTAACAAAAATTTAACGCGAATTTTAACAAAATATTAAC
GTTTACAATTTCAGGTGGCACTTTTCGGGGAAATGTGCGCGGAACCCCTAT

TTGTTTATTTTTCTAAATACATTCAAATATGTATCCGCTCATGAATTAATTC
TTAGAAAAACTCATCGAGCATCAAATGAAACTGCAATTTATTCATATCAG
GATTATCAATACCATATTTTTGAAAAAGCCGTTTCTGTAATGAAGGAGAA
AACTCACCGAGGCAGTTCCATAGGATGGCAAGATCCTGGTATCGGTCTGC
GATTCCGACTCGTCCAACATCAATACAACCTATTAATTTCCCCTCGTCAAA
AATAAGGTTATCAAGTGAGAAATCACCATGAGTGACGACTGAATCCGGTG
AGAATGGCAAAAGTTTATGCATTTCTTTCCAGACTTGTTCAACAGGCCAGC
CATTACGCTCGTCATCAAAATCACTCGCATCAACCAAACCGTTATTCATTC
GTGATTGCGCCTGAGCGAGACGAAATACGCGATCGCTGTTAAAAGGACAA
TTACAAACAGGAATCGAATGCAACCGGCGCAGGAACACTGCCAGCGCAT
CAACAATATTTTCACCTGAATCAGGATATTCTTCTAATACCTGGAATGCTG
TTTTCCCGGGGATCGCAGTGGTGAGTAACCATGCATCATCAGGAGTACGG
ATAAAATGCTTGATGGTCGGAAGAGGCATAAATTCCGTCAGCCAGTTTAG
TCTGACCATCTCATCTGTAACATCATTGGCAACGCTACCTTTGCCATGTTT
CAGAAACAACTCTGGCGCATCGGGCTTCCCATACAATCGATAGATTGTCG
CACCTGATTGCCCGACATTATCGCGAGCCCATTTATACCCATATAAATCAG
CATCCATGTTGGAATTTAATCGCGGCCTAGAGCAAGACGTTTCCCGTTGA
ATATGGCTCATAACACCCCTTGTATTACTGTTTATGTAAGCAGACAGTTTT
ATTGTTCATGACCAAAATCCCTTAACGTGAGTTTTCGTTCCACTGAGCGTC
AGACCCCGTAGAAAAGATCAAAGGATCTTCTTGAGATCCTTTTTTTCTGCG
CGTAATCTGCTGCTTGCAAACAAAAAAACCACCGCTACCAGCGGTGGTTT
GTTTGCCGGATCAAGAGCTACCAACTCTTTTTCCGAAGGTAACTGGCTTCA
GCAGAGCGCAGATACCAAATACTGTCCTTCTAGTGTAGCCGTAGTTAGGC
CACCACTTCAAGAACTCTGTAGCACCGCCTACATACCTCGCTCTGCTAATC
CTGTTACCAGTGGCTGCTGCCAGTGGCGATAAGTCGTGTCTTACCGGGTTG
GACTCAAGACGATAGTTACCGGATAAGGCGCAGCGGTCGGGCTGAACGG
GGGGTTCGTGCACACAGCCCAGCTTGGAGCGAACGACCTACACCGAACTG
AGATACCTACAGCGTGAGCTATGAGAAAGCGCCACGCTTCCCGAAGGGA
GAAAGGCGGACAGGTATCCGGTAAGCGGCAGGGTCGGAACAGGAGAGCG
CACGAGGGAGCTTCCAGGGGGAAACGCCTGGTATCTTTATAGTCCTGTCG
GGTTTCGCCACCTCTGACTTGAGCGTCGATTTTTGTGATGCTCGTCAGGGG
GGCGGAGCCTATGGAAAAACGCCAGCAACGCGGCCTTTTTACGGTTCCTG
GCCTTTTGCTGGCCTTTTGCTCACATGTTCTTTCCTGCGTTATCCCCTGATT
CTGTGGATAACCGTATTACCGCCTTTGAGTGAGCTGATACCGCTCGCCGCA
GCCGAACGACCGAGCGCAGCGAGTCAGTGAGCGAGGAAGCGGAAGAGCG
CCTGATGCGGTATTTTCTCCTTACGCATCTGTGCGGTATTTCACACCGCAT
ATATGGTGCACTCTCAGTACAATCTGCTCTGATGCCGCATAGTTAAGCCAG
TATACACTCCGCTATCGCTACGTGACTGGGTCATGGCTGCGCCCCGACACC
CGCCAACACCCGCTGACGCGCCCTGACGGGCTTGTCTGCTCCCGGCATCC
GCTTACAGACAAGCTGTGACCGTCTCCGGGAGCTGCATGTGTCAGAGGTT
TTCACCGTCATCACCGAAACGCGCGAGGCAGCTGCGGTAAAGCTCATCAG
CGTGGTCGTGAAGCGATTCACAGATGTCTGCCTGTTCATCCGCGTCCAGCT
CGTTGAGTTTCTCCAGAAGCGTTAATGTCTGGCTTCTGATAAAGCGGGCCA
TGTTAAGGGCGGTTTTTTCCTGTTTGGTCACTGATGCCTCCGTGTAAGGGG
GATTTCTGTTCATGGGGGTAATGATACCGATGAAACGAGAGAGGATGCTC
ACGATACGGGTTACTGATGATGAACATGCCCGGTTACTGGAACGTTGTGA
GGGTAAACAACTGGCGGTATGGATGCGGCGGGACCAGAGAAAAATCACT
CAGGGTCAATGCCAGCGCTTCGTTAATACAGATGTAGGTGTTCCACAGGG
TAGCCAGCAGCATCCTGCGATGCAGATCCGGAACATAATGGTGCAGGGCG
CTGACTTCCGCGTTTCCAGACTTTACGAAACACGGAAACCGAAGACCATT

CATGTTGTTGCTCAGGTCGCAGACGTTTTGCAGCAGCAGTCGCTTCACGTT
CGCTCGCGTATCGGTGATTCATTCTGCTAACCAGTAAGGCAACCCCGCCA
GCCTAGCCGGGTCCTCAACGACAGGAGCACGATCATGCGCACCCGTGGGG
CCGCCATGCCGGCGATAATGGCCTGCTTCTCGCCGAAACGTTTGGTGGCG
GGACCAGTGACGAAGGCTTGAGCGAGGGCGTGCAAGATTCCGAATACCG
CAAGCGACAGGCCGATCATCGTCGCGCTCCAGCGAAAGCGGTCCTCGCCG
AAAATGACCCAGAGCGCTGCCGGCACCTGTCCTACGAGTTGCATGATAAA
GAAGACAGTCATAAGTGCGGCGACGATAGTCATGCCCCGCGCCCACCGGA
AGGAGCTGACTGGGTTGAAGGCTCTCAAGGGCATCGGTCGAGATCCCGGT
GCCTAATGAGTGAGCTAACTTACATTAATTGCGTTGCGCTCACTGCCCGCT
TTCCAGTCGGGAAACCTGTCGTGCCAGCTGCATTAATGAATCGGCCAACG
CGCGGGGAGAGGCGGTTTGCGTATTGGGCGCCAGGGTGGTTTTTCTTTTCA
CCAGTGAGACGGGCAACAGCTGATTGCCCTTCACCGCCTGGCCCTGAGAG
AGTTGCAGCAAGCGGTCCACGCTGGTTTGCCCCAGCAGGCGAAAATCCTG
TTTGATGGTGGTTAACGGCGGGATATAACATGAGCTGTCTTCGGTATCGTC
GTATCCCACTACCGAGATATCCGCACCAACGCGCAGCCCGGACTCGGTAA
TGGCGCGCATTGCGCCCAGCGCCATCTGATCGTTGGCAACCAGCATCGCA
GTGGGAACGATGCCCTCATTCAGCATTTGCATGGTTTGTTGAAAACCGGA
CATGGCACTCCAGTCGCCTTCCCGTTCCGCTATCGGCTGAATTTGATTGCG
AGTGAGATATTTATGCCAGCCAGCCAGACGCAGACGCGCCGAGACAGAA
CTTAATGGGCCCGCTAACAGCGCGATTTGCTGGTGACCCAATGCGACCAG
ATGCTCCACGCCCAGTCGCGTACCGTCTTCATGGGAGAAAATAATACTGT
TGATGGGTGTCTGGTCAGAGACATCAAGAAATAACGCCGGAACATTAGTG
CAGGCAGCTTCCACAGCAATGGCATCCTGGTCATCCAGCGGATAGTTAAT
GATCAGCCCACTGACGCGTTGCGCGAGAAGATTGTGCACCGCCGCTTTAC
AGGCTTCGACGCCGCTTCGTTCTACCATCGACACCACCACGCTGGCACCC
AGTTGATCGGCGCGAGATTTAATCGCCGCGACAATTTGCGACGGCGCGTG
CAGGGCCAGACTGGAGGTGGCAACGCCAATCAGCAACGACTGTTTGCCCG
CCAGTTGTTGTGCCACGCGGTTGGGAATGTAATTCAGCTCCGCCATCGCCG
CTTCCACTTTTTCCCGCGTTTTCGCAGAAACGTGGCTGGCCTGGTTCACCA
CGCGGGAAACGGTCTGATAAGAGACACCGGCATACTCTGCGACATCGTAT
AACGTTACTGGTTTCACATTCACCACCCTGAATTGACTCTCTTCCGGGCGC
TATCATGCCATACCGCGAAAGGTTTTGCGCCATTCGATGGTGTCCGGGATC
TCGACGCTCTCCCTTATGCGACTCCTGCATTAGGAAGCAGCCCAGTAGTA
GGTTGAGGCCGTTGAGCACCGCCGCCGCAAGGAATGGTGCATGCAAGGA
GATGGCGCCCAACAGTCCCCCGGCCACGGGGCCTGCCACCATACCCACGC
CGAAACAAGCGCTCATGAGCCCGAAGTGGCGAGCCCGATCTTCCCCATCG
GTGATGTCGGCGATATAGGCGCCAGCAACCGCACCTGTGGCGCCGGTGAT
GCCGGCCACGATGCGTCCGGCGTAGAGGATCGAGATCTCGATCCCGCGAA
ATTAATACGACTCACTATAGGGGAATTGTGAGCGGATAACAATTCCCCTC
TAGAAATAATTTTGTTTAACTTTAAGAAGGAGATATACCATGGGCAGCAG
CCATCATCATCATCATCACAGCAGCGGCCTGGTGCCGCGCGGCAGCCAT
ATGCGCGCGCTGCGGGCCGGCCTGACCCTAGCGCTGGGCGCGGGGCTGGG
CGCCGCGGCAGAGCATTGGCGGCGGCGGGAGGGCAAAGCGCCGGGGCTG
CTGGGCCGAGTGCCATTGTTGCCGGTGGTCGCGGCCGATCTTCCCGCGCTG
CCGGGGGGACCGGCGGGCGGCACCGGGGAACTGGCCAAGTACGGGCTGC
CCGGCGTGGCGCAGCTCCGGAGCCGCGAGTCCTACGTGCTTAGCTACGAC
CCGCGCACGCGCGGTGCGCTCTGGGTGTTGGAGCAGCTGAGGCCAGAGCG
GCTCCGTGGCGACGGGGACCGTAGCGCCTGCGACTTCCGCGAGGATGACT
CTGTGCACGCGTACCACCGCGCCACCAATGCGGACTACCGCGGCAGTGGC

TTTGACCGCGGCGCTTTGGCCGCCGCCGCCAACCACCGCTGGAGTCAGCG
GGCCATGGACGACACCTTCTACCTGAGCAACGTAGCGCCTCAGGTGCCAC
ACCTCAACCAGAATGCCTGGAACAACCTTGAGAGGTACAGCCGCAGCTTG
ACGCGAACTTACCAAAATGTCTATGTCTGCACGGGGCCGCTTTTCCTGCCC
AGGACCGAGGCTGATGGGAAGTCCTATGTGAAGTACCAGGTTATTGGGAA
GAACCACGTGGCAGTGCCCACACACTTCTTCAAGGTGCTGATCCTGGAGG
CAGCCGGTGGGCAGATCGAGCTACGTTCCTACGTGATGCCCAATGCCCCC
GTGGATGAGACCATCCCTCTGGAGCGGTTCCTGGTGCCCATCGAGAGCAT
CGAGCGGGCCTCGGGATTGCTCTTCGTGCCCAATATTCTGGCTCGAGCTGG
AAACCTCAAGGCTATCACTGCTGGCAGCAAGTGAGCGGCCGCACTCGAGC
ACCACCACCACCACCACTGAGATCCGGCTGCTAACAAAGCCCGAAAGGA
AGCTGAGTTGGCTGCTGCCACCGCTGAGCAATAACTAGCATAACCCCTTG
GGGCCTCTAAACGGGTCTTGAGGGGTTTTTTGCTGAAAGGAGGAACTATA
TCCGGAT

pET28a-EndoGI

ATCCGGATATAGTTCCTCCTTTCAGCAAAAAACCCCTCAAGACCCGTTTAG
AGGCCCCAAGGGGTTATGCTAGTTATTGCTCAGCGGTGGCAGCAGCCAAC
TCAGCTTCCTTTCGGGCTTTGTTAGCAGCCGGATCTCAGTGGTGGTGGTGG
TGGTGCTCGAGTGCGGCCGCCTAATCATCATTTACAGGTCCTTCAAAGGG
AGCCTTGCAACCAGCCTTTTCGTAAAACTCGAATGGCAGCGGATCCTCGTT
GTCGTAATAGTCGGAATCGCGGGCGCAACTGGGACCAAGTTCCATTTTCA
CCATCTTGTCGCGCCAAAAGTCGAGAAGCTCTTTGGTGAGAACCTCATCC
CTCAGATTGAAAATCCAACTGTTGTAGTCGTCGGGCCCATAGAAACCAGC
ATCACCCTTGTACTCCTGCAGCAGCTCGGCGGCCTTGTTTTGATCCTCCGC
TGAGGGTGTGCCATCGAAGAGCAGCTTCCAGATTATCTCCCTGGCGGCTG
CCGCTATCTTCTTCACGCTGGCCTGGTGAACGCTGTCCGGAACGATGATTT
TGGTCAGAACAAAGCGCTTCATTAGAGTTCGGTATTCCTCGAGCGGATTTT
CCTTGGTCAGGGTGGCCTCAAATTCGCCAGACAGAGCGGCGGCATCATCG
GCACTCAGTTCACCCGGAGTTGTAGGCGTCGCTGGCCCATCCTGATCCAG
CGAAGCCTGCTCCTCGAGTGCTGCGCGCACCTTTAAGCTGGCTGCAAATG
GAGCGGTACAACCGGCATGGGCATAGAACTCCAATGGCGGGGTGTCGTCG
CTGTCAAAGAGATCACTGTCGCGTGACCAACAGGGACCGAGTTGCTTCTT
CACCAGCACATCGCGCCAAAAGTCGAGGAGTTCCTTCTTTAGAACCTCAT
CCCTCAGCTTGACAATCCATTCGTTGTACGGCGTCGGGTCGTAGAAGCAG
GCGTCACCACGGTATTCCTCCAGCAATTGGCTGGCCTTCTCTGTGTTCGAT
TGCGAGGTACCGGCCTCGTCGAATAACAGCCGCCAGATGACGTCACGCGC
CGCTGCAGCCGTTCGATCGAAATTCTCCTTGGTGACATCATCATCCACGAT
TTCGTCTACCTGCACGCGTGTTTCGATAAGCTTTTGGTAATCCTTAACGGG
ATCCACCAGGCCAATGGTGTAATCGTTCTGTGCTACTTTCTCGGCCGTTGC
CATTTTGTCGGATTGTGTGTCCTCGGCCTTGCGCTTGGACATATGGCTGCC
GCGCGGCACCAGGCCGCTGCTGTGATGATGATGATGATGGCTGCTGCCCA
TGGTATATCTCCTTCTTAAAGTTAAACAAAATTATTTCTAGAGGGGAATTG
TTATCCGCTCACAATTCCCCTATAGTGAGTCGTATTAATTTCGCGGGATCG
AGATCTCGATCCTCTACGCCGGACGCATCGTGGCCGGCATCACCGGCGCC
ACAGGTGCGGTTGCTGGCGCCTATATCGCCGACATCACCGATGGGGAAGA
TCGGGCTCGCCACTTCGGGCTCATGAGCGCTTGTTTCGGCGTGGGTATGGT
GGCAGGCCCCGTGGCCGGGGGACTGTTGGGCGCCATCTCCTTGCATGCAC
CATTCCTTGCGGCGGCGGTGCTCAACGGCCTCAACCTACTACTGGGCTGCT

TCCTAATGCAGGAGTCGCATAAGGGAGAGCGTCGAGATCCCGGACACCAT
CGAATGGCGCAAAACCTTTCGCGGTATGGCATGATAGCGCCCGGAAGAGA
GTCAATTCAGGGTGGTGAATGTGAAACCAGTAACGTTATACGATGTCGCA
GAGTATGCCGGTGTCTCTTATCAGACCGTTTCCCGCGTGGTGAACCAGGCC
AGCCACGTTTCTGCGAAAACGCGGGAAAAAGTGGAAGCGGCGATGGCGG
AGCTGAATTACATTCCCAACCGCGTGGCACAACAACTGGCGGGCAAACAG
TCGTTGCTGATTGGCGTTGCCACCTCCAGTCTGGCCCTGCACGCGCCGTCG
CAAATTGTCGCGGCGATTAAATCTCGCGCCGATCAACTGGGTGCCAGCGT
GGTGGTGTCGATGGTAGAACGAAGCGGCGTCGAAGCCTGTAAAGCGGCG
GTGCACAATCTTCTCGCGCAACGCGTCAGTGGGCTGATCATTAACTATCCG
CTGGATGACCAGGATGCCATTGCTGTGGAAGCTGCCTGCACTAATGTTCC
GGCGTTATTTCTTGATGTCTCTGACCAGACACCCATCAACAGTATTATTTT
CTCCCATGAAGACGGTACGCGACTGGGCGTGGAGCATCTGGTCGCATTGG
GTCACCAGCAAATCGCGCTGTTAGCGGGCCCATTAAGTTCTGTCTCGGCG
CGTCTGCGTCTGGCTGGCTGGCATAAATATCTCACTCGCAATCAAATTCAG
CCGATAGCGGAACGGGAAGGCGACTGGAGTGCCATGTCCGGTTTTCAACA
AACCATGCAAATGCTGAATGAGGGCATCGTTCCCACTGCGATGCTGGTTG
CCAACGATCAGATGGCGCTGGGCGCAATGCGCGCCATTACCGAGTCCGGG
CTGCGCGTTGGTGCGGATATCTCGGTAGTGGGATACGACGATACCGAAGA
CAGCTCATGTTATATCCCGCCGTTAACCACCATCAAACAGGATTTTCGCCT
GCTGGGGCAAACCAGCGTGGACCGCTTGCTGCAACTCTCTCAGGGCCAGG
CGGTGAAGGGCAATCAGCTGTTGCCCGTCTCACTGGTGAAAAGAAAAACC
ACCCTGGCGCCCAATACGCAAACCGCCTCTCCCCGCGCGTTGGCCGATTC
ATTAATGCAGCTGGCACGACAGGTTTCCCGACTGGAAAGCGGGCAGTGAG
CGCAACGCAATTAATGTAAGTTAGCTCACTCATTAGGCACCGGGATCTCG
ACCGATGCCCTTGAGAGCCTTCAACCCAGTCAGCTCCTTCCGGTGGGCGC
GGGGCATGACTATCGTCGCCGCACTTATGACTGTCTTCTTTATCATGCAAC
TCGTAGGACAGGTGCCGGCAGCGCTCTGGGTCATTTTCGGCGAGGACCGC
TTTCGCTGGAGCGCGACGATGATCGGCCTGTCGCTTGCGGTATTCGGAATC
TTGCACGCCCTCGCTCAAGCCTTCGTCACTGGTCCCGCCACCAAACGTTTC
GGCGAGAAGCAGGCCATTATCGCCGGCATGGCGGCCCCACGGGTGCGCAT
GATCGTGCTCCTGTCGTTGAGGACCCGGCTAGGCTGGCGGGGTTGCCTTA
CTGGTTAGCAGAATGAATCACCGATACGCGAGCGAACGTGAAGCGACTGC
TGCTGCAAAACGTCTGCGACCTGAGCAACAACATGAATGGTCTTCGGTTT
CCGTGTTTCGTAAAGTCTGGAAACGCGGAAGTCAGCGCCCTGCACCATTA
TGTTCCGGATCTGCATCGCAGGATGCTGCTGGCTACCCTGTGGAACACCTA
CATCTGTATTAACGAAGCGCTGGCATTGACCCTGAGTGATTTTTCTCTGGT
CCCGCCGCATCCATACCGCCAGTTGTTTACCCTCACAACGTTCCAGTAACC
GGGCATGTTCATCATCAGTAACCCGTATCGTGAGCATCCTCTCTCGTTTCA
TCGGTATCATTACCCCCATGAACAGAAATCCCCCTTACACGGAGGCATCA
GTGACCAAACAGGAAAAAACCGCCCTTAACATGGCCCGCTTTATCAGAAG
CCAGACATTAACGCTTCTGGAGAAACTCAACGAGCTGGACGCGGATGAAC
AGGCAGACATCTGTGAATCGCTTCACGACCACGCTGATGAGCTTTACCGC
AGCTGCCTCGCGCGTTTCGGTGATGACGGTGAAAACCTCTGACACATGCA
GCTCCCGGAGACGGTCACAGCTTGTCTGTAAGCGGATGCCGGGAGCAGAC
AAGCCCGTCAGGGCGCGTCAGCGGGTGTTGGCGGGTGTCGGGGCGCAGCC
ATGACCCAGTCACGTAGCGATAGCGGAGTGTATACTGGCTTAACTATGCG
GCATCAGAGCAGATTGTACTGAGAGTGCACCATATATGCGGTGTGAAATA
CCGCACAGATGCGTAAGGAGAAAATACCGCATCAGGCGCTCTTCCGCTTC
CTCGCTCACTGACTCGCTGCGCTCGGTCGTTCGGCTGCGGCGAGCGGTATC

AGCTCACTCAAAGGCGGTAATACGGTTATCCACAGAATCAGGGGATAACG
CAGGAAAGAACATGTGAGCAAAAGGCCAGCAAAAGGCCAGGAACCGTAA
AAAGGCCGCGTTGCTGGCGTTTTTCCATAGGCTCCGCCCCCCTGACGAGC
ATCACAAAAATCGACGCTCAAGTCAGAGGTGGCGAAACCCGACAGGACT
ATAAAGATACCAGGCGTTTCCCCCTGGAAGCTCCCTCGTGCGCTCTCCTGT
TCCGACCCTGCCGCTTACCGGATACCTGTCCGCCTTTCTCCCTTCGGGAAG
CGTGGCGCTTTCTCATAGCTCACGCTGTAGGTATCTCAGTTCGGTGTAGGT
CGTTCGCTCCAAGCTGGGCTGTGTGCACGAACCCCCCGTTCAGCCCGACC
GCTGCGCCTTATCCGGTAACTATCGTCTTGAGTCCAACCCGGTAAGACAC
GACTTATCGCCACTGGCAGCAGCCACTGGTAACAGGATTAGCAGAGCGAG
GTATGTAGGCGGTGCTACAGAGTTCTTGAAGTGGTGGCCTAACTACGGCT
ACACTAGAAGGACAGTATTTGGTATCTGCGCTCTGCTGAAGCCAGTTACC
TTCGGAAAAAGAGTTGGTAGCTCTTGATCCGGCAAACAAACCACCGCTGG
TAGCGGTGGTTTTTTTGTTTGCAAGCAGCAGATTACGCGCAGAAAAAAAG
GATCTCAAGAAGATCCTTTGATCTTTTCTACGGGGTCTGACGCTCAGTGGA
ACGAAAACTCACGTTAAGGGATTTTGGTCATGAACAATAAAACTGTCTGC
TTACATAAACAGTAATACAAGGGGTGTTATGAGCCATATTCAACGGGAAA
CGTCTTGCTCTAGGCCGCGATTAAATTCCAACATGGATGCTGATTTATATG
GGTATAAATGGGCTCGCGATAATGTCGGGCAATCAGGTGCGACAATCTAT
CGATTGTATGGGAAGCCCGATGCGCCAGAGTTGTTTCTGAAACATGGCAA
AGGTAGCGTTGCCAATGATGTTACAGATGAGATGGTCAGACTAAACTGGC
TGACGGAATTTATGCCTCTTCCGACCATCAAGCATTTTATCCGTACTCCTG
ATGATGCATGGTTACTCACCACTGCGATCCCCGGGAAAACAGCATTCCAG
GTATTAGAAGAATATCCTGATTCAGGTGAAAATATTGTTGATGCGCTGGC
AGTGTTCCTGCGCCGGTTGCATTCGATTCCTGTTTGTAATTGTCCTTTTAAC
AGCGATCGCGTATTTCGTCTCGCTCAGGCGCAATCACGAATGAATAACGG
TTTGGTTGATGCGAGTGATTTTGATGACGAGCGTAATGGCTGGCCTGTTGA
ACAAGTCTGGAAAGAAATGCATAAACTTTTGCCATTCTCACCGGATTCAG
TCGTCACTCATGGTGATTTCTCACTTGATAACCTTATTTTTGACGAGGGGA
AATTAATAGGTTGTATTGATGTTGGACGAGTCGGAATCGCAGACCGATAC
CAGGATCTTGCCATCCTATGGAACTGCCTCGGTGAGTTTTCTCCTTCATTA
CAGAAACGGCTTTTTCAAAAATATGGTATTGATAATCCTGATATGAATAA
ATTGCAGTTTCATTTGATGCTCGATGAGTTTTTCTAAGAATTAATTCATGA
GCGGATACATATTTGAATGTATTTAGAAAAATAAACAAATAGGGGTTCCG
CGCACATTTCCCCGAAAAGTGCCACCTGAAATTGTAAACGTTAATATTTTG
TTAAAATTCGCGTTAAATTTTTGTTAAATCAGCTCATTTTTTAACCAATAG
GCCGAAATCGGCAAAATCCCTTATAAATCAAAAGAATAGACCGAGATAG
GGTTGAGTGTTGTTCCAGTTTGGAACAAGAGTCCACTATTAAAGAACGTG
GACTCCAACGTCAAAGGGCGAAAAACCGTCTATCAGGGCGATGGCCCACT
ACGTGAACCATCACCCTAATCAAGTTTTTTGGGGTCGAGGTGCCGTAAAG
CACTAAATCGGAACCCTAAAGGGAGCCCCCGATTTAGAGCTTGACGGGGA
AAGCCGGCGAACGTGGCGAGAAAGGAAGGGAAGAAAGCGAAAGGAGCG
GGCGCTAGGGCGCTGGCAAGTGTAGCGGTCACGCTGCGCGTAACCACCAC
ACCCGCCGCGCTTAATGCGCCGCTACAGGGCGCGTCCCATTCGCCA

Supplemental Table S2

Primers to create substrates		
	Amplify pUC19	
	pUC19 For	5′-GGTACCCGGGGATCCTCTAGAGT-3′
	pUC19 Rev	5′-ACTCTAGAGGATCCCCGGGTACC-3′
	Amplify pCR2.1	
	pCR2.1 Amp For	5'-TTGTCAGAAGTAAGTTGGTCGCAGTGTTATC-3'
	pCR2.1 Amp Rev	5'-GATAACACTGCGACCAACTTACTTCTGACAA-3'
	Recombination A For	5'-GATAACACTGCGACCAACTTACTTCTGACAA-3'
	Recombiantion A Rev	5'-TTGTCAGAAGTAAGTTGGTCGCAGTGTTATC-3'
qPCR Primers		
	Amplify Cut Region of pCR2.1 (Amplicon I)	
	Amplicon I For	5'-GCTATGACCATGATTACGCCAAGC-3'
	Amplicon I Rev	5'-TACGACTCACTATAGGGCGAATTGGG-3'
	Uncut Control Region of pCR2.1 (Amplicon II)	
	Ampicon II For	5′-ATGGATATCTGCAGAATTCGCC-3′
	Amplicon II Rev	5′-AGTGTGCTGGAATTCGCC-3′
	Recombiantion Assay Control Amplicon	
	Recombination Control For	5'-AGCTTGGCGTAATCATGGTCA-3'
	Recombination Control Rev	5'-GGGGGAAACGCCTGGTATCTT-3'
	Recombination Assay Recombined Region	
	Recombined For	5'-CGGTGAAAACCTCTGACACA-3'
	Recombined Rev	5'-TGCAGGTCGACTCTAGAGGAT-3'
Oligonucleotides for assays		
	Top Unmodified	5'-AGGGCTTACCATCTGGCCCCAGTGCTGCAATGATACCGCGAGACC-3'
	Bottom Unmodified	5'-GGTCTCGCGGTATCATTGCAGCACTGGGGCCAGATGGTAAGCCCT-3'
	Top 5hmC pos 1	5'-AGGGCTTACCATCTGG5hmCCCCAGTGCTGCAATGATACCGCGAGACC-3'
	Top 5hmC pos 2	5'-AGGGCTTACCATCTGGC5hmCCCAGTGCTGCAATGATACCGCGAGACC-3'
	Top 5hmC pos 3	5'-AGGGCTTACCATCTGGCC5hmCCAGTGCTGCAATGATACCGCGAGACC-3'
	Top 5hmC pos 4	5'-AGGGCTTACCATCTGGCCC5hmCAGTGCTGCAATGATACCGCGAGACC-3'
	Bottom 5hmC pos 5	5'-GGTCTCGCGGTATCATTGCAGCACTGGGG5hmCCAGATGGTAAGCCCT-3'
	Bottom 5hmC pos 6	5'-GGTCTCGCGGTATCATTGCAGCACTGGGGC5hmCAGATGGTAAGCCCT-3'
Site Directed Mutagenesis Primers		
	EndoGH128AFor	5'-CTTTGACCGCGGCGCTTTGGCCGCCGCCGCC-3'
	EndoGH128ARev	5'-GGCGGCGGCGGCCAAAGCGCCGCGGTCAAAG-3'

Supplemental Table S3

Gene ID	Protein Name
gi:49864	alpha-actin (aa 40-375) [Mus musculus]
gi:50797	unnamed protein product [Mus musculus]
gi:50814	unnamed protein product [Mus musculus]
gi:50881	ezrin [Mus musculus]
gi:51263	p68 RNA helicase [Mus musculus]
gi:52785	unnamed protein product [Mus musculus]
gi:52787	unnamed protein product [Mus musculus]
gi:52789	unnamed protein product [Mus musculus]
gi:52865	unnamed protein product [Mus musculus]
gi:52867	lamin B2 [Mus musculus]
gi:52869	unnamed protein product [Mus musculus]
gi:54827	immunoglobulin gamma 2b heavy chain [Mus musculus]
gi:110434	Ig kappa chain V region (G2a) - mouse
gi:116132	CFAH_MOUSE RecName: Full=Complement factor H; AltName: Full=Protein beta-1-H; Flags: Precursor
gi:191765	alpha-fetoprotein, partial [Mus musculus]
gi:192005	apolipoprotein E, partial [Mus musculus]
gi:197102	(V-J). Ig heavy chain V-region [Mus musculus]
gi:201725	t complex polypeptide 1 [Mus musculus]
gi:220349	C4 [Mus musculus]
gi:226165	beta hexosaminidase beta
gi:227527	Ig VH ICH2, anti-angiotensin II
gi:227530	Ig VL ICH2, anti-angiotensin II
gi:228591	lamin B2
gi:284921	Ig light chain V region (clone 17s.83) - mouse (fragment)
gi:309119	C4b-binding protein precursor [Mus musculus]
gi:309319	heat shock protein 70 cognate [Mus musculus]
gi:312005	small nuclear ribonucleoprotein E [Mus musculus]
gi:346859	Ig kappa chain V region - mouse (fragment)

gi:347839	matricin [Mus musculus]
gi:398168	keratin 2 epidermis [Mus musculus]
gi:460317	chaperonin [Mus musculus]
gi:548879	RSU1_MOUSE RecName: Full=Ras suppressor protein 1; Short=RSP-1; Short=Rsu-1
gi:555835	HMG CoA synthase, partial [Mus musculus]
gi:556301	elongation factor Tu [Mus musculus]
gi:575667	unnamed protein product [Mus musculus]
gi:763157	U1RNA-associated 70-kDa protein [Mus musculus]
gi:793931	tryptophan-2,3-dioxygenase [Mus musculus]
gi:904215	cytokeratin 15 [Mus musculus]
gi:1083440	octamer-binding protein NonO - mouse
gi:1125026	3-hydroxyacyl CoA dehydrogenase [Mus musculus]
gi:1150880	phospholipase C beta3 [Mus musculus]
gi:1181242	fibronectin [Mus musculus]
gi:1304155	pokeweed agglutinin-binding protein [Mus musculus]
gi:1438563	SH3P9 [Mus musculus]
gi:1524165	NfiC1B [Mus musculus]
gi:1526541	14-3-3 eta [Mus musculus]
gi:1685271	RXR alpha 2 [Mus musculus]
gi:1772998	C1 inhibitor [Mus musculus]
gi:1816635	SRG3 [Mus musculus]
gi:2392474	A Chain A, Trivalent Antibody Fragment
gi:2465310	DNA helicase II [Mus musculus]
gi:2497642	CLC4F_MOUSE RecName: Full=C-type lectin domain family 4 member F
gi:2644957	SA2 nuclear protein [Mus musculus]
gi:2739450	integrin binding protein kinase [Mus musculus]
gi:2773081	sarcoplasmic reticulum Ca2+-ATPase [Mus musculus]
gi:2961456	RNA helicase A [Mus musculus]
gi:3023934	HDAC2_MOUSE RecName: Full=Histone deacetylase 2; Short=HD2; AltName: Full=YY1 transcription factor-binding protein
gi:3242657	cysteinyl-tRNA synthetase [Mus musculus]

gi:3329496	heterogenous nuclear ribonucleoprotein U [Mus musculus]
gi:3329498	heterogenous nuclear ribonucleoprotein A2/B1 [Mus musculus]
gi:3834675	interleukin enhancer binding factor 3 [Mus musculus]
gi:4001805	BAF53a [Mus musculus]
gi:4159806	type II keratin subunit protein [Mus musculus]
gi:4388775	radixin [Mus musculus]
gi:4506005	serine/threonine-protein phosphatase PP1-beta catalytic subunit isoform 1 [Homo sapiens]
gi:4507131	small nuclear ribonucleoprotein F [Homo sapiens]
gi:4590328	AF087141_1 valyl-tRNA synthetase [Mus musculus]
gi:4759158	small nuclear ribonucleoprotein Sm D2 isoform 1 [Homo sapiens]
gi:4759160	small nuclear ribonucleoprotein Sm D3 [Homo sapiens]
gi:5020213	AF149822_1 mitotic checkpoint protein BUB3 [Mus musculus]
gi:5031595	actin-related protein 2/3 complex subunit 4 isoform a [Homo sapiens]
gi:5263198	GARP45 [Mus musculus]
gi:5295992	chaperonin containing TCP-1 theta subunit [Mus musculus]
gi:5453555	GTP-binding nuclear protein Ran [Homo sapiens]
gi:5689158	Septin6 [Mus musculus]
gi:5902663	elongation factor 1-beta homolog [Mus musculus]
gi:6009521	p100 co-activator [Mus musculus]
gi:6671702	T-complex protein 1 subunit epsilon [Mus musculus]
gi:6678329	protein-glutamine gamma-glutamyltransferase 2 [Mus musculus]
gi:6679647	endonuclease G, mitochondrial precursor [Mus musculus]
gi:6680748	ATP synthase subunit alpha, mitochondrial precursor [Mus musculus]
gi:6753324	T-complex protein 1 subunit zeta [Mus musculus]
gi:6753738	eukaryotic translation initiation factor 2 subunit 3, X-linked [Mus musculus]
gi:6754222	heterogeneous nuclear ribonucleoprotein A/B isoform 2 [Mus musculus]
gi:6754480	keratin, type I cytoskeletal 13 [Mus musculus]
gi:6754816	septin-2 isoform a [Mus musculus]
gi:6754976	peroxiredoxin-1 [Mus musculus]
gi:6755372	40S ribosomal protein S3 [Mus musculus]

gi:6755382	ruvB-like 2 [Mus musculus]
gi:7021537	U2 small nuclear ribonucleoprotein A' [Mus musculus]
gi:7329989	nebulin [Mus musculus]
gi:7638398	AF245658_1 epidermal keratin 10 [Mus musculus]
gi:8393544	heterogeneous nuclear ribonucleoproteins C1/C2 isoform 1 [Mus musculus]
gi:8393784	septin-9 isoform c [Mus musculus]
gi:8567342	retinol dehydrogenase 7 precursor [Mus musculus]
gi:9055218	pre-mRNA-processing factor 40 homolog A [Mus musculus]
gi:9506571	eukaryotic translation initiation factor 2 subunit 1 [Rattus norvegicus]
gi:9717245	cytoplasmic dynein heavy chain [Mus musculus]
gi:9790069	spliceosome RNA helicase Ddx39b [Mus musculus]
gi:9790141	actin-related protein 2/3 complex subunit 3 [Mus musculus]
gi:9910294	keratin, type II cytoskeletal 71 [Mus musculus]
gi:9957546	AF179996_1 Sep2 [Mus musculus]
gi:10181166	SWI/SNF-related matrix-associated actin-dependent regulator of chromatin subfamily E member 1 [Mus musculus]
gi:10442545	PTB-associated splicing factor [Mus musculus]
gi:11907833	AF257474_1 betaine-homocysteine methyltransferase 2 [Mus musculus]
gi:12835914	unnamed protein product [Mus musculus]
gi:12836375	unnamed protein product [Mus musculus]
gi:12841593	unnamed protein product [Mus musculus]
gi:12843914	unnamed protein product [Mus musculus]
gi:12845562	unnamed protein product [Mus musculus]
gi:12845960	unnamed protein product [Mus musculus]
gi:12847801	unnamed protein product [Mus musculus]
gi:12848426	unnamed protein product [Mus musculus]
gi:12851426	unnamed protein product [Mus musculus]
gi:12856949	unnamed protein product [Mus musculus]
gi:12859782	unnamed protein product [Mus musculus]
gi:12860388	unnamed protein product [Mus musculus]
gi:12964610	pre-mRNA processing 8 protein [Mus musculus]

gi:13124192	EF1D_MOUSE RecName: Full=Elongation factor 1-delta; Short=EF-1-delta
gi:13384620	heterogeneous nuclear ribonucleoprotein K [Mus musculus]
gi:13385872	interleukin enhancer-binding factor 2 [Mus musculus]
gi:13386106	cleavage and polyadenylation specificity factor subunit 5 [Mus musculus]
gi:13435498	Tars protein, partial [Mus musculus]
gi:13435984	Serine hydroxymethyltransferase 2 (mitochondrial) [Mus musculus]
gi:13442965	AF247132_1 putative chromatin remodeling factor [Mus musculus]
gi:13445784	AF340028_1 Rab6-interacting protein 2 isoform A [Mus musculus]
gi:13959396	IQGA1_MOUSE RecName: Full=Ras GTPase-activating-like protein IQGAP1
gi:14250408	Aspartyl-tRNA synthetase [Mus musculus]
gi:15077863	AF396878_1 bullous pemphigoid antigen 1-a [Mus musculus]
gi:15214281	SF3B1_MOUSE RecName: Full=Splicing factor 3B subunit 1; AltName: Full=Pre-mRNA-splicing factor SF3b 155 kDa subunit
gi:15488701	Unknown (protein for IMAGE:4219618), partial [Mus musculus]
gi:15723268	AF403565_1 fructose-bisphosphate aldolase B [Mus musculus]
gi:16303309	type II keratin 5 [Mus musculus]
gi:16506251	elongation factor-like protein [Mus musculus]
gi:19482166	glycine N-acyltransferase-like protein Keg1 [Mus musculus]
gi:19527078	fibrinogen gamma chain precursor [Mus musculus]
gi:19527174	splicing factor 3B subunit 3 [Mus musculus]
gi:19527358	pre-mRNA-processing factor 19 isoform 2 [Mus musculus]
gi:20070408	glycine dehydrogenase [decarboxylating], mitochondrial precursor [Mus musculus]
gi:20071242	BC026782 protein [Mus musculus]
gi:20072624	Heterogeneous nuclear ribonucleoprotein L [Mus musculus]
gi:20810027	Acetyl-Coenzyme A acyltransferase 2 (mitochondrial 3-oxoacyl-Coenzyme A thiolase) [Mus musculus]
gi:21314854	transcriptional repressor p66-beta [Mus musculus]
gi:21704042	cleavage stimulation factor subunit 3 isoform 1 [Mus musculus]
gi:22087520	AF513713_1 memory-related protein [Mus musculus]
gi:22095003	splicing factor 3A subunit 3 [Mus musculus]
gi:22122795	cytoplasmic dynein 1 light intermediate chain 1 [Mus musculus]
gi:22164776	keratin, type II cytoskeletal 79 [Mus musculus]

gi:23272966	Atp5b protein [Mus musculus]
gi:23510313	wiskott-Aldrich syndrome protein family member 2 [Mus musculus]
gi:23822106	RRBP1_MOUSE RecName: Full=Ribosome-binding protein 1; AltName: Full=Ribosome receptor protein; Short=RRp; Short=mRRp
gi:23956214	splicing factor, proline- and glutamine-rich [Mus musculus]
gi:24657723	Threonyl-tRNA synthetase-like 2 [Mus musculus]
gi:24943086	TPA_exp: nuclear pore complex-associated intranuclear coiled-coil protein TPR [Mus musculus]
gi:25137573	rootletin [Mus musculus]
gi:26324430	unnamed protein product [Mus musculus]
gi:26324732	unnamed protein product [Mus musculus]
gi:26324776	unnamed protein product [Mus musculus]
gi:26327587	unnamed protein product [Mus musculus]
gi:26337387	unnamed protein product [Mus musculus]
gi:26344926	unnamed protein product [Mus musculus]
gi:26345348	unnamed protein product [Mus musculus]
gi:26345990	unnamed protein product [Mus musculus]
gi:26346400	unnamed protein product [Mus musculus]
gi:26346949	unnamed protein product [Mus musculus]
gi:26349515	unnamed protein product [Mus musculus]
gi:26350305	unnamed protein product [Mus musculus]
gi:26352267	unnamed protein product [Mus musculus]
gi:26354124	unnamed protein product [Mus musculus]
gi:26383339	unnamed protein product [Mus musculus]
gi:27369537	glypican-2 precursor [Mus musculus]
gi:28144914	GTPase, IMAP family member 9 [Mus musculus]
gi:28972155	mKIAA0325 protein [Mus musculus]
gi:29467640	ankyrin repeat hooked to a zinc finger motif long form [Mus musculus]
gi:31321923	androgen-induced prostate proliferative shutoff associated protein AS3 [Mus musculus]
gi:31559916	heterogeneous nuclear ribonucleoprotein A3 isoform a [Mus musculus]
gi:31982273	peroxisomal multifunctional enzyme type 2 [Mus musculus]
gi:33563252	fibrinogen, alpha polypeptide isoform 2 precursor [Mus musculus]

gi:33563288	cell division cycle and apoptosis regulator protein 1 [Mus musculus]
gi:36031035	structural maintenance of chromosomes protein 3 [Mus musculus]
gi:37360362	mKIAA1398 protein [Mus musculus]
gi:37360612	mKIAA2016 protein [Mus musculus]
gi:38372875	FINC_MOUSE RecName: Full=Fibronectin; Short=FN; Contains: RecName: Full=Anastellin; Flags: Precursor
gi:38372907	ATP-dependent RNA helicase DDX39A [Mus musculus]
gi:39204553	chromodomain-helicase-DNA-binding protein 4 [Mus musculus]
gi:40018610	U5 small nuclear ribonucleoprotein 200 kDa helicase [Mus musculus]
gi:40068493	probable ATP-dependent RNA helicase DDX17 isoform 1 [Mus musculus]
gi:40849918	plectin 6 [Mus musculus]
gi:41946089	Eif4g1 protein [Mus musculus]
gi:46485130	TPA_exp: keratin Kb40 [Mus musculus]
gi:47059013	keratin, type II cytoskeletal 73 [Mus musculus]
gi:49022852	mKIAA0991 protein [Mus musculus]
gi:50401169	PNKP_MOUSE RecName: Full=Bifunctional polynucleotide phosphatase/kinase; AltName: Full=DNA 5'-kinase/3'-phosphatase
gi:55740400	leucine-rich repeat kinase 2 [Mus musculus]
gi:56800176	chromodomain helicase DNA binding protein 3 [Mus musculus]
gi:62738645	A Chain A, Molecular Architecture Of Mammalian Polynucleotide Kinase, A Dna Repair Enzyme
gi:66792896	sorting nexin-32 [Mus musculus]
gi:67846113	U1 small nuclear ribonucleoprotein 70 kDa [Mus musculus]
gi:67906179	fermitin family homolog 2 [Mus musculus]
gi:68059559	NEUA_MOUSE RecName: Full=N-acylneuraminate cytidylyltransferase; AltName: Full=CMP-N-acetylneuraminic acid synthase
gi:70778915	moesin [Mus musculus]
gi:71153505	DHX9_MOUSE RecName: Full=ATP-dependent RNA helicase A; Short=RHA; AltName: Full=DEAH box protein 9; Short=mHEL-5
gi:74137669	unnamed protein product [Mus musculus]
gi:74141789	unnamed protein product [Mus musculus]
gi:74141990	unnamed protein product [Mus musculus]
gi:74143690	unnamed protein product [Mus musculus]
gi:74148166	unnamed protein product [Mus musculus]
gi:74181043	unnamed protein product [Mus musculus]

gi:74181057	unnamed protein product [Mus musculus]
gi:74181154	unnamed protein product [Mus musculus]
gi:74190887	unnamed protein product [Mus musculus]
gi:74193982	unnamed protein product [Mus musculus]
gi:74198568	unnamed protein product [Mus musculus]
gi:74203337	unnamed protein product [Mus musculus]
gi:74209075	unnamed protein product [Mus musculus]
gi:74217870	unnamed protein product [Mus musculus]
gi:74219697	unnamed protein product [Mus musculus]
gi:74219852	unnamed protein product [Mus musculus]
gi:77812697	titin isoform N2-A [Mus musculus]
gi:77812699	titin isoform N2-B [Mus musculus]
gi:81867214	GPR98_MOUSE RecName: Full=G-protein coupled receptor 98
gi:81892832	CENPE_MOUSE RecName: Full=Centromere-associated protein E
gi:93587673	probable ATP-dependent RNA helicase DDX17 isoform 4 [Mus musculus]
gi:110625979	elongation factor 1-gamma [Mus musculus]
gi:112363072	actin-related protein 2/3 complex subunit 2 [Mus musculus]
gi:116256516	heterogeneous nuclear ribonucleoprotein D0 isoform d [Mus musculus]
gi:116283440	Rdx protein [Mus musculus]
gi:122114537	vacuolar protein sorting-associated protein 13C [Mus musculus]
gi:123232325	nebulin [Mus musculus]
gi:123246601	calmodulin binding transcription activator 1 [Mus musculus]
gi:123248483	chromodomain helicase DNA binding protein 5 [Mus musculus]
gi:124248512	carbamoyl-phosphate synthase [ammonia], mitochondrial precursor [Mus musculus]
gi:134152676	tensin-3 [Mus musculus]
gi:145580629	keratin Kb40 [Mus musculus]
gi:145699091	nesprin-2 [Mus musculus]
gi:148222065	nebulin [Mus musculus]
gi:148671336	threonyl-tRNA synthetase, isoform CRA_a [Mus musculus]
gi:148675530	structural maintenance of chromosomes 1A, isoform CRA_b [Mus musculus]

gi:148685279	RIKEN cDNA E030013G06, isoform CRA_d [Mus musculus]
gi:148686927	mCG21601 [Mus musculus]
gi:148687563	mCG12425, isoform CRA_a [Mus musculus]
gi:148691129	mCG1030 [Mus musculus]
gi:148692621	SWI/SNF related, matrix associated, actin dependent regulator of chromatin, subfamily c, member 2, isoform CRA_a [Mus musculus]
gi:148693016	mCG142052, isoform CRA_b [Mus musculus]
gi:148694957	mCG9866 [Mus musculus]
gi:148695270	titin [Mus musculus]
gi:148695542	nucleoporin 160, isoform CRA_a [Mus musculus]
gi:148697229	mCG119749, isoform CRA_a [Mus musculus]
gi:148697232	mCG1035404 [Mus musculus]
gi:148701638	septin 8, isoform CRA_c [Mus musculus]
gi:156630942	SPEF2_MOUSE RecName: Full=Sperm flagellar protein 2; AltName: Full=Protein KPL2
gi:157879307	L Chain L, Bactericidal Antibody Against Neisseria Meningitidis
gi:160358754	TITIN_MOUSE RecName: Full=Titin; AltName: Full=Connectin
gi:183980004	heterogeneous nuclear ribonucleoprotein L [Mus musculus]
gi:205277432	E3 ubiquitin-protein ligase HECTD1 [Mus musculus]
gi:226443091	heterogeneous nuclear ribonucleoprotein A0 [Mus musculus]
gi:255003678	RING finger protein 169 [Mus musculus]
gi:258613892	structural maintenance of chromosomes protein 1A [Mus musculus]
gi:340708199	A Chain A, Crystal Structure Of A Heterogeneous Nuclear Ribonucleoprotein L (Hnrpl) From Mus Musculus At 2.15 A Resolution
